# Recent Progress in Improving Rate Performance of Cellulose-Derived Carbon Materials for Sodium-Ion Batteries

**DOI:** 10.1007/s40820-024-01351-2

**Published:** 2024-03-11

**Authors:** Fujuan Wang, Tianyun Zhang, Tian Zhang, Tianqi He, Fen Ran

**Affiliations:** 1 State Key Laboratory of Advanced Processing and Recycling of Non-Ferrous Metals, Lanzhou University of Technology, Lanzhou, 730050 People’s Republic of China; 2 School of Materials Science and Engineering, Lanzhou University of Technology, Lanzhou, 730050 People’s Republic of China; 3 School of Mechanical and Electronical Engineering, Lanzhou University of Technology, Lanzhou, 730050 People’s Republic of China

**Keywords:** Cellulose, Hard carbon, Anode materials, Rate performance, Sodium-ion batteries

## Abstract

Cellulose-derived carbon is regarded as one of the most promising candidates for high-performance anode materials in sodium-ion batteries; however, its poor rate performance at higher current density remains a challenge to achieve high power density sodium-ion batteries. The present review comprehensively elucidates the structural characteristics of cellulose-based materials and cellulose-derived carbon materials, explores the limitations in enhancing rate performance arising from ion diffusion and electronic transfer at the level of cellulose-derived carbon materials, and proposes corresponding strategies to improve rate performance targeted at various precursors of cellulose-based materials. This review also presents an update on recent progress in cellulose-based materials and cellulose-derived carbon materials, with particular focuses on their molecular, crystalline, and aggregation structures. Furthermore, the relationship between storage sodium and rate performance the carbon materials is elucidated through theoretical calculations and characterization analyses. Finally, future perspectives regarding challenges and opportunities in the research field of cellulose-derived carbon anodes are briefly highlighted.

## Highlights

- Enhancing rate performance of cellulose-derived hard carbon anodes from the view of cellulose molecular, crystalline, and aggregation structure is explored.
- Relationship of storage sodium and rate performance according to theoretical calculation and characterization analysis is illustrated.
- Cellulose intrinsic microstructure, conversion relationship between the allotropes of cellulose, and the critical influences on cellulose-derived carbon structure are discussed.

## Graphical abstract

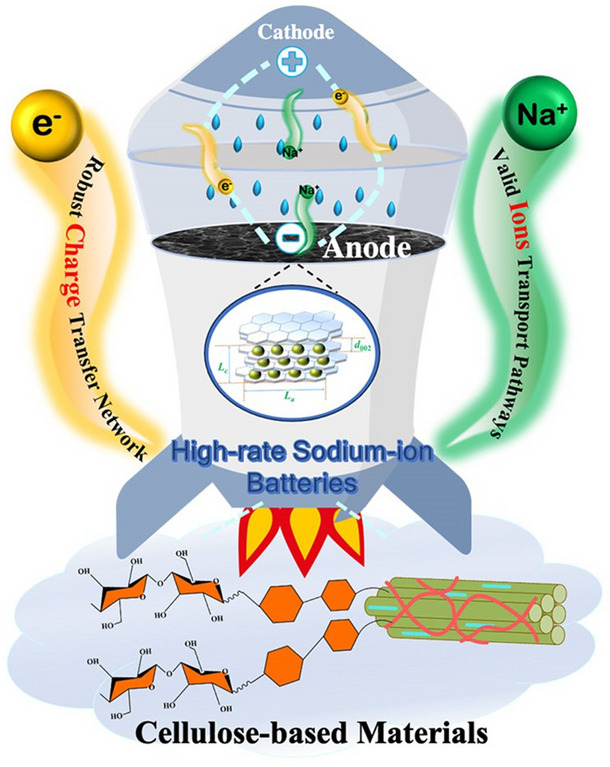

## Introduction

There are alarming consequences that the burning of fossil fuels and biomass and the gaseous emissions can lead to environmental pollution and global warming [1]. Utilizing carbon coupling with other elements, nature creates diverse creatures and provides renewable ways for energy and matter transformation [2]. These carbon-based natural resources, such as cellulose, lignin, and so on, have been used as renewable precursors for the preparation of carbon materials. Moreover, due to the short supply of traditional energy materials, alternative energy sources and their environmental availability have garnered significant interest [3]. In either energy source, the energy carriers are all based on the smart grid and chemical energy devices. As one type of chemical energy device, batteries with outstanding portability and conversion efficiency are considered superstar devices, that can convert chemical energy to electricity directly [4].

Due to their high safety, wide voltage, and good capacity, secondary batteries such as lithium-ion batteries (LIBs) and sodium-ion batteries (SIBs) have been drawing much attention from scientists [5, 6]. SIBs as alternative batteries are now being developed owing to the excessive use and the shortage of lithium resources [7]. Sodium and lithium are located in the same main group and own similar physiochemical properties, and the distribution of sodium resources is homogeneous and abundant. Unfortunately, the heavier mass and larger radius of sodium ion make the electrochemical performance of SIBs incomparable to that of LIBs. The increasing importance of rate performance for practical applications and commercialization of SIBs is often overlooked, especially the specific capacity at a high rate, that is rate performance. Whereas a higher rate performance is a necessary condition for SIBs to achieve fast-charging devices. Besides, commercial graphite, which is typically an anode of LIBs, cannot be used to storage sodium ions due to several thermodynamic reasons. Obviously, the study of anode materials’ rate performance has a far-reaching influence on commercial applications of SIBs.

The progress in discovering appropriate anode materials for SIBs has significantly been accelerated. Carbonaceous materials mainly including graphite, soft carbon, and hard carbon from the view of graphitization degree, have been generally investigated ascribing to their accessibility, non-toxicity, and chemical stability. Graphite possesses a well-structured arrangement [8], wherein carbon layers are composed of *sp*^2^ hybridized carbon atoms. The longitudinal carbon layers are held together by Van der Waals forces, maintaining a layer spacing of 0.335 nm [9]. Soft carbon, which is graphitizable non-graphitic carbon at the higher temperature, have more defects, and the inner carbon layer structure is characteristic of short-range ordered and long-range stacked [10]. Hard carbon, first mentioned by Dahn’s groups in 2000, plays an essential role in studying storage mechanisms of carbon anode materials in SIBs. At present, a good many precursors have been employed for fabricating hard carbons involving polymers like polyacrylonitrile, resinous like epoxy resin and phenolic resin, and biomass materials like roots, stems, and leaves of plants. These kinds of carbons exhibit different electrochemical performances on account of various microstructures [11]. When it comes to the precursors of hard carbon, biomass materials with abundant reserves are of major concerns, especially cellulose-based materials [12].

For the past several decades, cellulose-based materials have been used as the typical precursors to produce hard carbon materials owing to their unique network structures, high flexibility and a low coefficient of thermal expansion. Wu and co-authors investigate the relationship between the structural parameters and surface chemistry of cellulose-derived carbons, as well as their sodium storage performances [13]. It is showed that microcrystalline of cellulose-derived carbon exhibits a larger graphite size, leading to the increase of reversible capacity. The detail strengths over other type of carbon materials are as follows. (i) Cellulose is made up of D-glucose units that is an efficient carbon source. When carbonized at high temperature, cellulose forms a carbon-rich skeleton and conductive network. Due to the natural orientation and crystallinity of cellulose, the well-ordered and oriented graphitic structures with a high *sp*^2^ carbon content is formed. (ii) The pyrolysis process of cellulose mainly involves breaking hydrogen bonds at low carbonization temperature, formation of intermediate cellulose, and depolymerization and ring-opening of pyranoid ring at high temperature [14]. During the carbonization process, the surface-abundant hydroxy groups can be a great advantage for toughening and combining. The resulting abundant C=O groups of cellulose-derived carbon is beneficial to improve rate performance. (iii) The hierarchical structure is constructed through hydrogen bonds and Van der Waals forces of cellulose microfibril. After carbonization, the formed abundant pores and large specific surface area can be utilized as channels for ion diffusion and charge transfer during the battery operation. And (iv) cellulose-based materials generally have a complex composition, which always acts as sacrificial templates during carbonization, that favor increasing the specific surface area and forming hierarchical pore structure [15].

Considering the potential application of hard carbon derived from cellulose and its derivatives in anode materials for SIBs. Many efforts have been devoted to explore structure of cellulose and its derived carbon materials in order to improve reversible capacity and rate performance of carbon anodes [16, 17]. This review focus on expounding the cellulose intrinsic microstructure and conversion relationship between the allotropes of cellulose, and dissecting the critical influences on cellulose-derived carbon structure. The relationship of storage sodium and rate performance according to the theoretical calculation and characterization analysis is illustrated. It classifies the limitations on improving rate performance in terms of ion diffusion and electronic transfer at cellulose-based carbon materials levels. Furthermore, different strategies for improving rate performance at cellulose materials levels based on above-mentioned limitations are sorted. Finally, it provides a summary of challenges and perspectives for future research in the field of cellulose-derived carbon for sodium-ion battery anode.

## Cellulose and Cellulose-Derived Carbon Materials

### Physical and Chemical Structures of Cellulose-Based Materials

Cellulose is unique natural polymers, and mainly photosynthesized in higher plants and synthesized by some bacteria, fungi, algae, and unicellular plants and animals [18, 19]. It is interesting that the tunicate is the only animal species obtained cellulose in the outer tissues [19]. Bacterial cellulose, produced extracellularly by Gram negative bacteria [20], is an important nano-cellulose [21]. Hence, cellulose generally exists in higher plant, such as bryophyte, pteridophyte, and seed plant. Although the cell walls of plants are the main source of cellulose, cellulose is surrounded by portion of lignin, hemicellulose, and ash. The cellulose content of plant is distinct, with those in wood (softwood: 40–50%; and hardwood: 45–50%) being lower than those in other plants (60–75%) [18, 22]. For example, the cellulose content of cotton fiber is from 94 to 95%, and that of kapok fiber is only 64% [23]. The specific proportions contents of cellulose from different sources are shown in Table 1.

**Table 1:** The proportion of cellulose in different raw materials

Raw materials	Cellulose	Hemi-cellulose	Lignin	References
Wood	40–50%	13–32%	27–32%	[24]
Bamboo	40–55%	14–25%	16–34%	[14]
Flax	62.1%	16.7%	1.8%	[25]
Hemp	67%	16.1%	0.8%	
Jute	70–75%	12.0%	0.2%	[23]
Ramie	68.8%	13.1%	1.9%	
Kapok	64.4%	22–45%	19%	
Cotton	92.7%	5.7%	–	[24]
Bacterial cellulose	100%	0	0	[20]

**Fig. 1:**
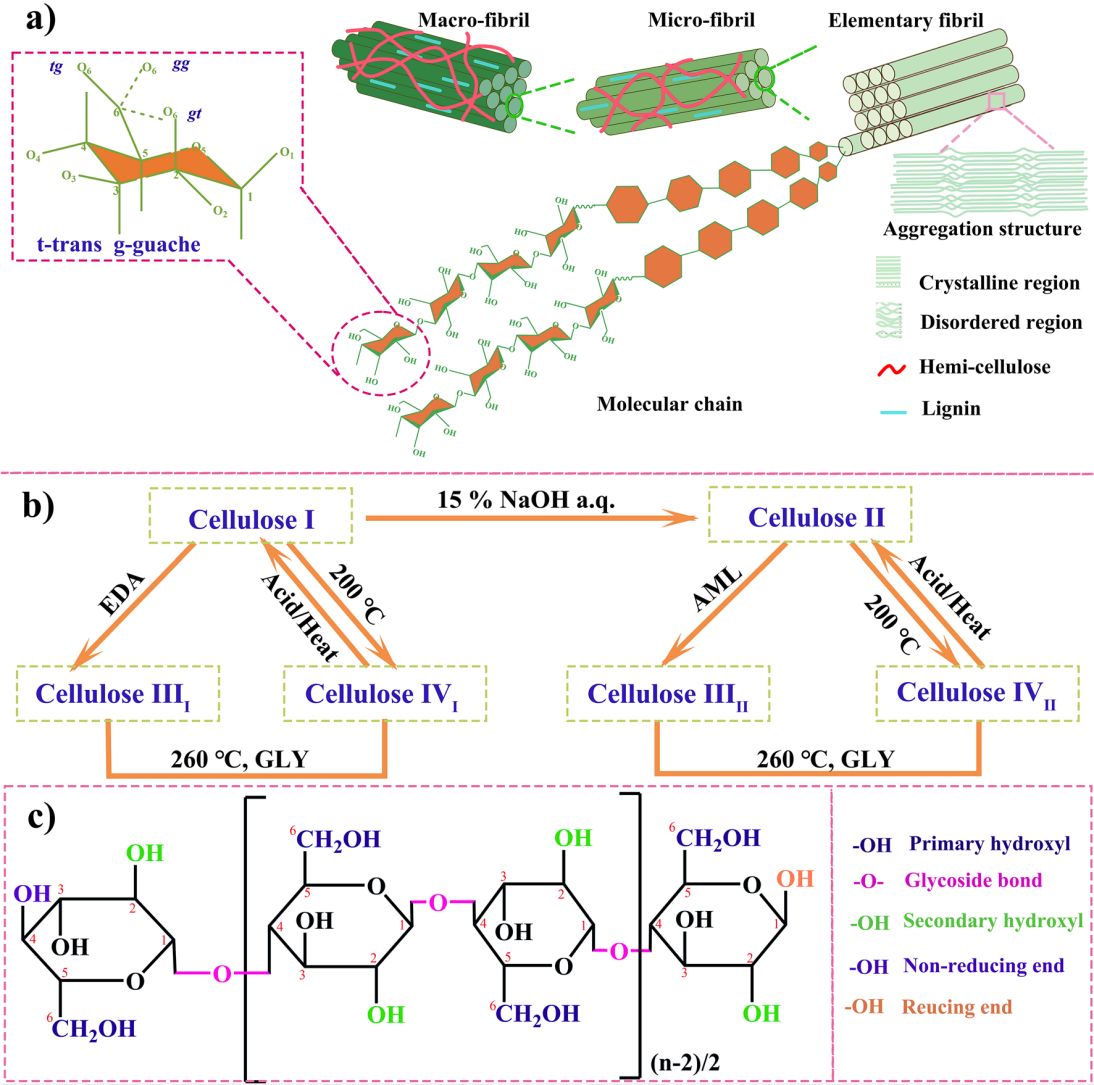
**a** The supra-molecular structure and aggregation structure of cellulose (the primary three conformations of hydroxyl group in the insert Fig.); **b** conversion between homogeneous polycrystalline of cellulose (the abbreviation of EDA, GLY and AML refer to ethanediamine, glycerinum and liquid ammonia; and **c** the reaction active groups of cellulose molecular chains

Clearly, cellulose is composited of repeating cellobiose units linked by *β* − 1, 4 glycosidic bonds. The glucose units are classified into *α*-glucose and *β*-glucose according to the position of hydroxyl group on the six-membered ring (Fig. 1a). Their difference is that the –OH group of *α*-glucose attaches at C-1 and C-4 position above the ring, whereas that of *β*-glucose locates at C-1 below the ring. It is well known that the chair formation of glucose is more stable. This is because the C-1 conformation with –OH group locating near ring plane (*equatorial hydroxyls*) is preferred, and the coordinate perpendicular to ring plane (*axial groups*) tends to instability [26]. The glycoside bond between the glucose units has the property of acetal, is easy to break in the chemical reaction, and the break of the glycoside bond causes degradation of cellulose molecules.

The molecular chain conformation shows various internal rotation heteromorphs. Due to the internal rotation effect, the arrangement of atoms in the molecule structure is constantly changing. In cellulose chains, C–O bond at C-6 has three different conformations, where *g* and *t* refer to guache and trans, respectively. For instance, in the *gt* conformation, C–O bond at C-6 locates in the side of C–O bond at C-5 and at the opposite direction of C–C bond of C5 and C-4. In general, the conformation of natural cellulose is *gt* conformation, while regenerated cellulose is *tg*, *gt*, and *gg* conformation, as seen in the insertion enlarged view of Fig. 1a. According to the molecule structure conformation, cellulose can form a well-ordered structure. On the one hand, the linear configuration of cellulose chain depends on hydrogen bonding stability between hydroxyl groups and oxygens of the adjoining ring molecules. On the other hand, cellulose fibril as the basic unit of building cellulose is a bundle of 10–30 nm in diameter, which assembled roughly by several elementary fibrils or micro-fibrils in parallel.

In order of size and stacking, cellulose fibril involves elementary fibrils, micro-fibrils, fibrils, macro-fibrils, cells and fibers [27], as shown in Fig. 1a. Elementary fibril, also known as whisker, is formed by a number of long-chain molecules bounding together through intermolecular Van der Waals and hydrogen bonds in parallel or spirals, and its diameter is about 1–3 nm [28]. Micro-fibril with the diameter of about 4–8 nm, also named micro-whiskers, formed by several elementary fibrils interacting with intermolecular forces in parallel arrangement [29]. Macro-fibril is structural block consisting of multiple micro-fibrils or fibril pairs with radical dimensions of 0.1–0.6 μm [30]. Cell is directly formed by macro-fibrils or micro-fibrils stacking with distinct cell border. Finally, a relatively stable cellulose fibril is formed and join together with two aggregated regions, including ordered (crystalline or orientation-aligned region and disordered (amorphous or anisotropic-aligned region [31]. It is because of fibrillar structure of cellulose materials, hierarchical porous structure and semi-crystalline structure are formed, and have advantages in anodes of SIBs.

Cellulose is a semi-crystalline material, and the cellulose chains in amorphous region linking the intermolecular hydrogen bonds between at C-2 and C-3, are in a random coil conformation [32]. The size of crystal cell is the furthest distance of crystal planes in a microfiber, determines the exposure degree of cellulose chain in the crystalline region. The degree of crystallinity is the ratio of crystal region, depending on its origin, extraction, and pretreatment. Although the density of amorphous region of cellulose is lower than that of the crystalline region, the amorphous region is more likely to react with other molecular groups, which plays an important role in functional design for cellulose-based carbon materials. Meanwhile, the crystalline structure of cellulose has influence on the physicochemical properties and crystalline structure of cellulose-based carbon materials, and provide a perspective for fabricating cellulose-derived carbon anode materials.

There are four different allomorphs of crystalline cellulose named as cellulose I, II, III, and IV, respectively. Their molecular chain structure and repetition distance are almost the same, but the size of crystal cell and stacking of molecular chain are differences. The detailed structure parameters are listed in Table 2. The natural cellulose I crystals are presented in the form of cellulose *I*_*α*_ with a triclinic structure and I_*β*_ with a monoclinic structure [33, 34]. Cellulose *I*_*α*_ accounts for the major component of bacterial or algal cellulose, and tunicates is based on cellulose *I*_*β*_. In higher plants, both cellulose *I*_*α*_ and *I*_*β*_ are present, locating in the cell wall of primary and secondary layers, respectively. Cellulose *I*_*α*_ has a higher crystallinity than *I*_*β*_, while the stability of cellulose *I*_*β*_ is stronger than that of *I*_*α*_, that is because cellulose *I*_*β*_ is arranged by the reverse parallel molecular chains to form a more stable linear structure. Hence, it is general that cellulose *I*_*β*_ is used as the matrix and reinforcing phases to prepare cellulose composites. In order to achieve composite materials with high crystalline and strength, the different crystalline structure of cellulose should be considered [35]. Here, let’s take flax composed of cellulose I and lyocell derived from cellulose II as exemplars. Although the lyocell fiber with low hemicellulose is significantly weaker strength, it has more effective in accelerating crystallinity and formation of stereo complexed crystallites than flax fibers. Due to its good reinforcing ability and low variability, it is a promising cellulose-based precursors in sustainable application of cellulose-based carbon materials.

**Table 2:** Lattice parameters of the most common cellulose allomorphs

Allomorph	Crystal system	Chain arrangement	*a* (nm)	*b* (nm)	*c* (nm)	*α* (°)	*β* (°)	*γ* (°)	References
Cellulose *I*_*α*_	One chain triclinic	Parallel	0.674	0.593	1.036	117	113	81	[36]
Cellulose *I*_*β*_	Two chain monoclinic	Parallel	0.801	0.817	1.036	90	90	97.3	
Cellulose II	Two chain monoclinic	Antiparallel	0.81	0.903	1.031	90	90	117.1	[37]
Cellulose III_I_	One chain monoclinic	Parallel	0.448	0.785	1.031	90	90	106.96	[38]
Cellulose III_II_	Two chain monoclinic	Antiparallel	1.025	0.778	1.034	90	90	122.4	[39]
Cellulose IV_I_	Two chains								
Orthorhombic	Parallel	0.803	0.813	1.034	90	90	90	[40]	
Cellulose IV_II_	Two chains								
Orthorhombic	Antiparallel	0.799	0.810	1.034	90	90	90		

The transformation relationship of various cellulose homogeneous polycrystalline is exhibited in Fig. 1b. Based on these, cellulose II is a more favorable thermodynamical structure than cellulose I. Because of weaker stacking interactions, cellulose II possesses a higher reactivity and cellulose III has more accessible channels on crystalline surface. Cellulose IV_I_ was certified by NMR spectra, and the results show that it is lateral disordered of cellulose *I*_*β*_ [41]. The order direction of cellulose IV_I_ chain is retained but its structure is paracrystalline and disorder in a lateral direction that is quite similar to *I*_*β*_. Liu et al. studied the crystalline structure make effects on degree of hydrolysis of the prepared different crystal cellulose [42]. The hydrolysis rates of four different crystalline cellulose allomorphs are in the order of II > III > I > IV. Because cellulose I and IV suffer surface reactions at 478–508 K, while cellulose II and III just swell at that temperature. The hydrolysis reaction occurring in the whole swollen region leads to higher accessibility between the glycosidic bond and the H^+^ catalyst. The various cellulose crystals have different hydroxyl arrangements, which generate molecular chains of diversified polarity. The conversion from parallel chains to antiparallel chains by physicochemical reactions result in the entropy increasing and enthalpy decreasing, whose process is an irreversible reaction.

Apart from the physical structure of cellulose, its chemical composition also plays a pivotal role in determining numerous properties of carbon materials derived from cellulose. The basic molecular format of cellulose is (C_6_H_10_O_5_)_*n*_ and the *n* refers to degree of polymerization (DP), that is the units number of hydro-glucose per polymer [43]. The various cellulose materials have different DP value depending on the source of cellulose. The cellulose materials in lab are designed with a lower DP, while natural cellulose such as cotton has a DP ranging from 3000 to 15,000. In general, the DP value of cellulose is closely linked with its strengthen and solubility due to a higher DP value with more tighten molecular structure, the higher strength with longer chain and more difficulty post-treatment.

In addition, three -OH groups play an important part in forming to the highly crystalline regions because of hydrogen bonding actions. It is also determined how difficult to treat cellulose in post-processing. Cellulose is insoluble in most aqueous solvents, but the abundant hydroxyl functional groups on the surface make it good hydrophilic and easy to chemically modify. The cellulose molecule chains groups involved in the chemical reaction include three hydroxyl groups on the glucose group, glycoside bonds to the glucose group, and two hydroxyl groups on the cellulose end. Figure 1c describes the reacting active groups of cellulose molecular chain.

There are many chemical modifications methods about cellulose to prepare cellulose-derived materials via etherification, esterification, oxidation, nucleophilic substitution, and grafting copolymerization [22]. The hydroxyl functional groups on cellulose chains positions C_2_, C_3_, and C_6_ are the focus of cellulose modification. The primary (OH–C_6_) and secondary (OH–C_2_ and OH–C_3_) hydroxyl groups have different chemical reaction abilities, whose reactivities different in the sequence of OH–C_6_ > OH–C_2_ > OH–C_3_ [22]. For instance, the method of TEMPO selection oxidized cellulose is prior to oxidize OH–C_6_ and OH–C_6_ is easier to occur substitution reactions than other hydroxyl groups [31]. Moreover, the reaction rate of OH–C_6_ is ten times faster than that of OH–C_2_ and OH–C_3_ in the esterification reaction, while the reaction rate of OH–C_2_ is twice as fast as that of OH–C_3_. There are two types of terminal-ends in a cellulose chain. The reducing end, in which the C_1_ hydroxyl is presented in the form of glycoside hydroxyl group has the property of hemiacetal. C_4_ hydroxyl group is free in the non-reducing end, and not involved in any linkage and retains the properties of hydroxyl group [44]. Due to the high DP values of most cellulose and the little proportion of terminal-ends, the effect of terminal-ends on the cellulose could be ignored.

### Pyrolysis Process and Influence Factors of Cellulose-Based Materials

Standing on the cost-efficient perspective, the cost of cellulose-based materials is less than resin and pitch-based materials. More importantly, the cellulose-based materials derived from natural resources have been employed as renewable precursors for the controlled synthesis of carbon materials, thereby significantly reducing reliance on fossil reserves and promoting sustainable development in human society. Pyrolysis carbonization, hydrothermal carbonization, and microwave-assisted are common strategies to generate cellulose-derived carbon materials. Compare with pyrolysis carbonization, hydrothermal carbonization and microwave-assisted carbonization methods required rigid experimental environment, resulting in higher production and consumed cost. Therefore, pyrolysis is one of the most classical and simple methods towards large-scale converting cellulose to carbon anode materials used in SIBs. Taking account of conductivity requirement for carbon materials used as anodes, the carbonization temperature prefers to be above 1000 °C [13]. To better understand of the relationship between cellulose-derived carbon’s structure and performance, it is necessary to illustrate the pyrolysis process of cellulose.

**Fig. 2:**
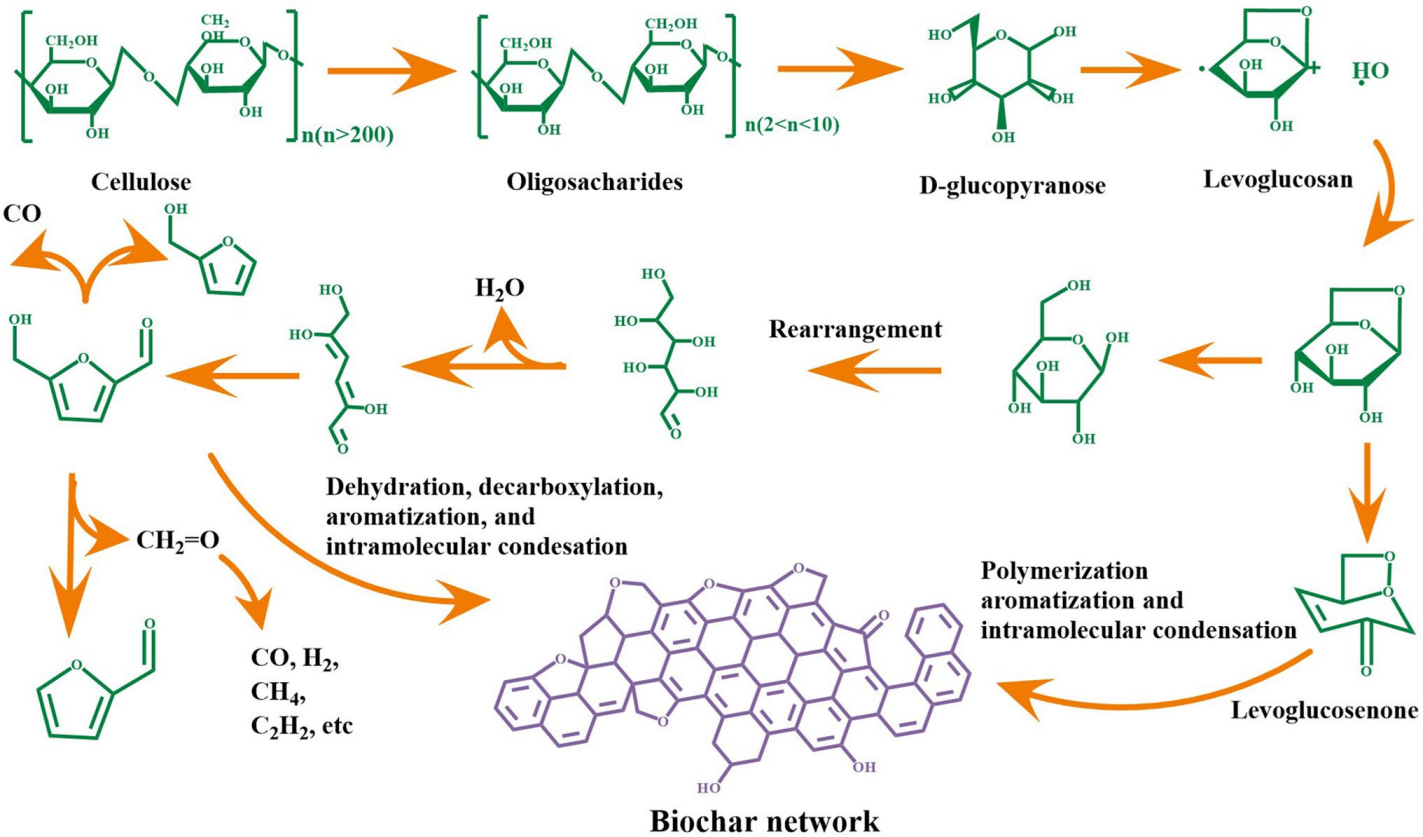
The pyrolysis process and reaction path of cellulose at high temperature [49]. Adapted with permission [49]. Copyright 2015, American Chemical Society

The mechanisms of cellulose pyrolysis and its derived carbon formation can be seen in Fig. 2. Firstly, cellulose is depolymerized to oligosaccharides, and the glycosidic bonds are cleaved to *D*-glucopyranose [45]. Then, it undergoes intramolecular rearrangement to form levoglucosan, which can form the solid carbon material through various pathways, such as dehydration, decarboxylation, aromatization, and intramolecular condensation [46]. The pyrolysis process exhibits a distinct characteristic of reduced degree of polymerization. Levoglucosan dominates the by-products of pyrolyzed cellulose I and II, but its concentration decreases with decreasing crystallinity [47]. The large amount of hydroxyl of cellulose is very easy to degrade into volatile compounds (CO, CO_2_, H_2_O, and some hydrocarbon, etc.) at low temperatures (≤ 400 °C [48]. Meanwhile, these processes usually produce many oxygen-containing heterocyclic rings, which readily transform into aromatic rings through dehydration, decarboxylation, and de-carbonylation reactions as temperature increases, benefitting the formation of cellulose-based derived carbon with interconnected microstructures.

**Fig. 3:**
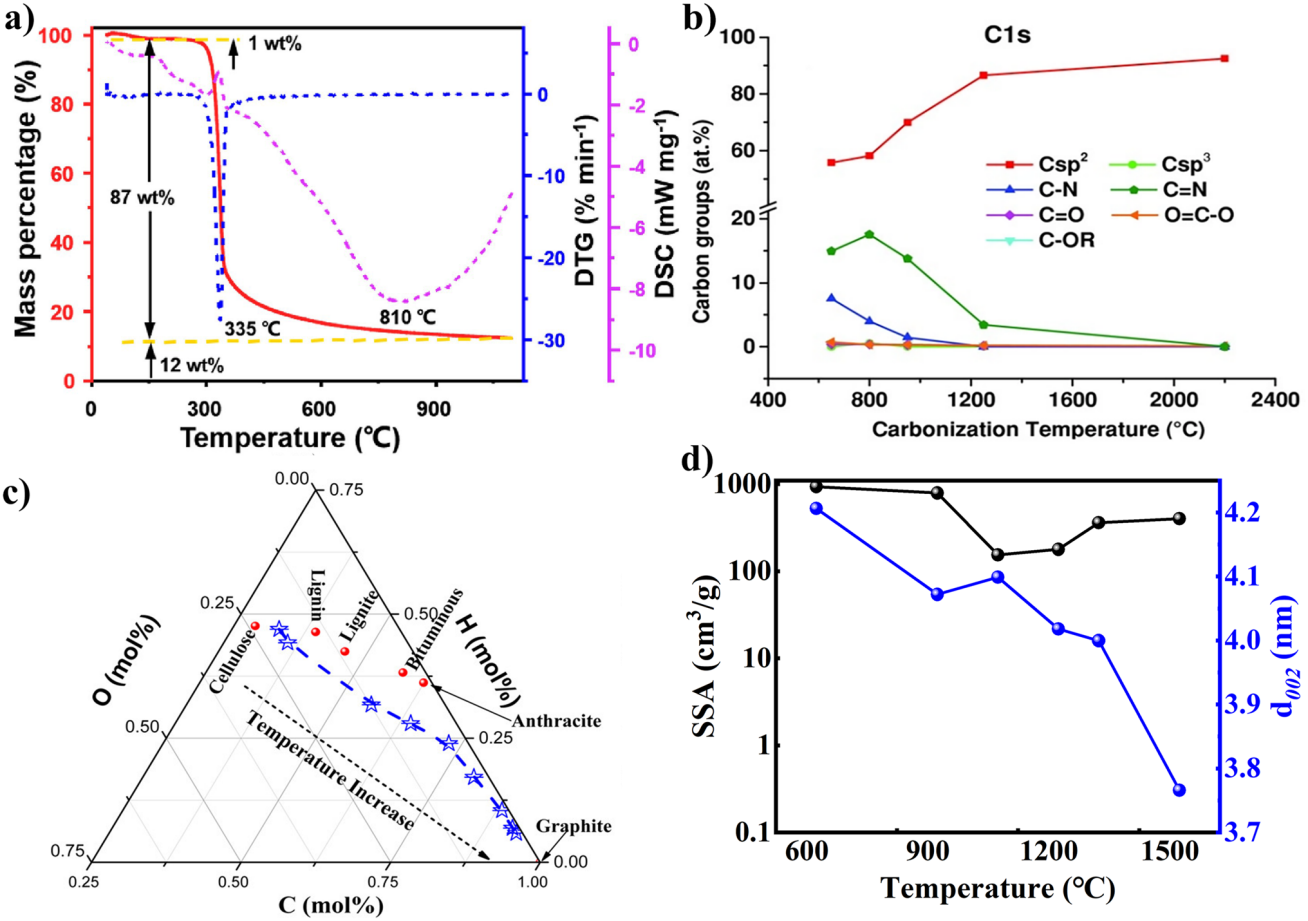
Evolution of cellulose-based carbon as pyrolysis temperature changing: **a** TGA, DTG, and DSC curves of cellulose pyrolysis[13]; **b** the containing different carbon groups [50]; **c** ternary phase diagram of C, H, O during cellulose pyrolysis evolution [14]; **d** SSA and *d*_002_ values [51]; **a** adapted with permission [13], Copyright 2022, Elsevier Limited. **b** adapted with permission [50], Copyright 2015, WILEY‐VCH Verlag GmbH & Co. KGaA, Weinheim; **c** adapted with permission [14], Copyright 2016, American Chemical Society; **d** adapted with permission [51], Copyright 2016, Elsevier B.V. and Science Press

The thermogravimetric analysis (TGA), thermogravimetric differentiation (DTG, and differential scanning calorimetry (DSC curves of cellulose are shown in Fig. 3a, and the pyrolysis process of cellulose is consistent with the above conclusions. According to the C 1*s* XPS in Fig. 3b, removal of O functional groups, including carbonyl (C=O), anhydrides (O–C=O), and ether (C–OR) groups, are the main reaction occurring below 1000 °C [50]. In the cellulose pyrolysis process, the changing functional groups and bond types are following water evaporation and volatile fractions at 200–500 °C. The degradation of organic matter with stronger chemical bonds in cellulose appeared at 500 °C or higher temperature [46]. Figure 3c illustrates the ternary phase diagram of C/H/O hard carbon derived from bamboo [14]. The temperature region of 250–450 °C corresponding conversion with carbon element increasing accord with the cellulose pyrolysis mechanism. In Fig. 3d, it is obvious that the specific surface area (SSA) gradually decreases and distance of graphene planes become closer with the increase in treatment temperature [51]. When the carbonization temperature over 1300 °C, SSA keeps a value of 10 cm^3^ g^−1^. Pyrolysis can cause noticeable changes in both surface functional group and inter/intramolecular bonds of cellulose.

Apart those, cellulose pyrolysis process includes three secondary reactions, that is polycondensation, multiphase interaction, and gaseous phase decomposition. The depolymerization path of cellulose and cleavage reaction of pyran rings play an important role in the cellulose pyrolysis products during secondary reactions [52, 53]. These two reactions happen at stage of higher temperature. Depolymerization is process in which cellulose is converted to dehydrated sugars with low degree of polymerization. Levoglucosan as the most important dehydrated sugar, has the highest yield of more than 60%, and a large amount of works focus on that cellulose produces different ways of depolymerization of levoglucosan [54]. The process of pyranoid ring opening is an important reaction of cellulose pyrolysis, which involves the formation mechanism of most of the small molecular components of tar except dehydrated sugars [52]. Since levoglucosan is the main product of cellulose pyrolysis, and secondary decomposition can produce small molecular products similar to cellulose pyrolysis, many scholars believe that levoglucosan is the primary product of cellulose pyrolysis, while other small molecules of organic matters come from secondary decomposition [55].

The cellulose crystallinity impacts on pyrolysis process of cellulose-based materials [47]. Antal et al. proposed the crystalline degree of cellulose would make an effect on carbon solid residues formation. It is because that the cellulose pyrolysis product, levoglucosan, can form the liquid intermediate when the temperature reaches at 300–340 °C of boiling points [56]. The liquid intermediate would poly-condense into carbon materials during pyrolysis that is critical for the prepared oligo-saccharides products [57]. Higher crystallinity degree improves original temperature of cellulose decompose and inhibits intermediate state of cellulose formation, due to good thermal stability caused by hydrogen bonds network [58]. However, the substance of non-crystallinity region in intermediate state can also provide an opportunity for dehydration reaction, and boost to generate furans type materials and high carbon residue ratio [58–60]. Wu et al. discussed pyrolysis behavior of amorphous and crystalline regions of cellulose [59]. It is illustrated that the crystalline cellulose with strong hydrogen bonding networks could preserve the sugar ring structure and obtained maximum of ~ 30% on a carbon basis at 250 °C, and that the amorphous cellulose with weak hydrogen bonds allow the liberation of these short glucose chain segments as pyrolysis intermediates, and a maximum of ∼ 3% on a carbon basis at 270 °C. Yu and co-workers used density functional theory (DFT) to investigate the location of hydroxyl groups has a influence on the dehydration of cellulose [61]. Hydroxy group of C-2 is prone to dehydration reactions such as pinanol. The intramolecular hydrogen bonds between C_3_–OH and C_5_–OH is more stable compared with other hydrogen bonds.

In term of the crystallization morphology of cellulose, Donohoe et al. studied different cellulose crystallization morphology (cellulose I refers to natural cellulose, cellulose II and III correspond to natural cellulose I treated by ionic liquids and anhydrous ammonia, respectively), which displayed cellulose pyrolysis behaviors and products during pyrolysis process [47]. Compared to cellulose-I or II, cellulose-III produces large amount of the same products regardless of its de-crystallization and depolymerization and produces as much or more levoglucosan at all crystallinity (different degree of de-crystallization and depolymerization of cellulos**e** levels. Besides, the different cellulose allomorphs also affect the viscoelastic properties of cellulose during pyrolysis. All in all, cellulose pyrolysis is a complex process. Breaking glycosidic linkage and opening pyran rings happen in different conditions, that would generate various carbon structure and of course occasionally certain byproducts.

### Morphology, Crystalline, and Molecular Structures of Cellulose-Derived Carbons

**Fig. 4:**
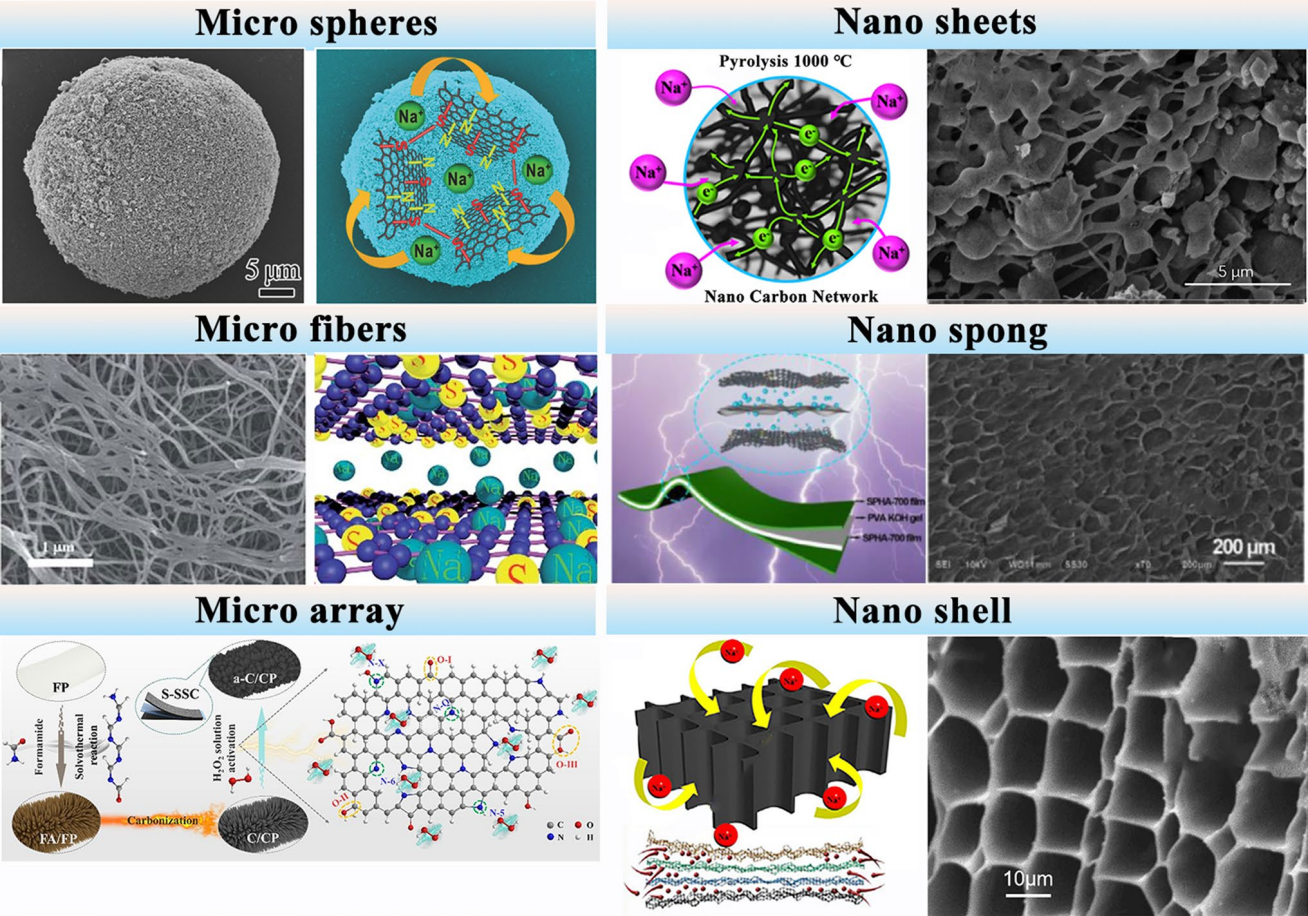
Various structures of cellulose-based carbon materials, including microspheres derived from wood-based cellulose carbon [66], nanosheets derived from cellulose acetate and kraft lignin [67], microfibers derived from bacterial cellulose [63], nanosponge derived from stem pith of helianthus annuus [68], microarray derived from filter paper [69], and nanoshell derived from cellulose [64]. Microspheres: adapted with permission [66], Copyright 2016, WILEY‐VCH Verlag GmbH & Co. KGaA, Weinheim. Nanosheets: adapted with permission [67], Copyright 2018, American Chemical Society. Microfibers: adapted with permission [63], Copyright 2019, the Royal Society of Chemistry. Nanosponge: adapted with permission [68], Copyright 2021, Elsevier Limited on behalf of Chinese Society for Metals. Microarray: adapted with permission [69], Copyright 2022 Elsevier B.V. Nanoshell: adapted with permission [64, 70], Copyright 2018, Elsevier B.V

The structure of cellulose-based carbon materials can be controlled by precursors’ structure of cellulose, carbonization temperature, heating speed, gas flow, and carbonization methods [62]. These various structures such as microspheres, microfibers, microarray hole, nanosheets, nanoshells, and nanosponge are shown in Fig. 4, caused by the diverse cellulose-based materials precursors and unique crystalline linear structure. Cellulose-derived carbon could keep precursors’ original microfiber-like morphology at high temperature pyrolysis following slow heating rate, which facilitates ion migration and adsorption [63]. For example, cotton-based cellulose with hollow structure easily formed hollow structure carbon. Besides, the nanoshells are synthesized using machine assistance, such as electrostatic spinning with coaxial spinnerets to produce core–shell fibers. The resulting fiber electrode consists of a cellulose shell and a CMK-3/S(carbon/sulfur) composite core, where the cellulose shell exhibits excellent ion conductivity and can accommodate volumetric expansion effectively [64]. Bacterial cellulose can be converted to highly conductive graphitic carbon by high-temperature carbonization, and the obtained bacterial cellulose-derived carbon could perfectly inherit the intrinsic interconnected 3D microfibers structure of precursor [65].

Of note that the proportion of cellulose and lignin in cellulose-based plant affects the carbon structure [49, 71]. Lignin, as a significant component in plant, contains fewer oxygen atoms and more aromatic rings. After carbonization, it can be easily converted into carbon materials with fewer micropores. According to the proportion of lignin, cellulose-derived carbon materials include wood-based [62, 72], cotton-based [73], bamboo-based [74], and nanocellulose-based [75, 76]. Wood-based cellulose-derived carbons are of two types-hardwood and softwood. For hardwood, its pristine structure with more crystalline cellulose scaffolds can be preserved in the carbonization process and only a few carbon nanoparticles can be found in the stable cell wall. Softwood always contains more lignin component (25–30%) that is easy to be hydrolyzed. As a result, the cell wall and plant tissue scaffold of hardwood will break down into small pieces, and then some globular porous carbon nanoparticles form [45]. Besides, more lignin would benefit from preparing stable nonporous carbon materials by direct pyrolysis due to its high aromatic ring contents. The cellulose and hemicellulose containing abundant hydroxyl groups would undergo decomposition and dehydration condensation, which would lead to the formation of plentiful micropores during the pyrolysis [48]. Thus, the plant-based cellulose carbon shows both honeycomb and compact structure, ascribed to hemicellulose and other impurity as sacrificial template during pyrolysis.

It is well known that cellulose-based materials precursors with rich oxygen or thermosetting, which tend to be rigid interconnected microstructure and hinder growth of parallel carbon layer. Therefore, the cellulose-based hard carbon has the characteristics of turbulence (turbostratically) and disordered structure. The oxygen groups play a subtle role in the formation of carbon. In general, oxygen mainly exists in the carbon layer, with the functional groups, such as ketone (–C=O), phenol, hydroxyl, and ether (–C–O), and carboxyl groups (–O–C=O–). These oxygen groups could be transferred and degraded to support ion adsorption and diffusion. The mechanism of oxygen groups for sodium ions adsorption is –C=O + Na^+^ + e^−^ = –C=O–Na. When the carbonized temperature exceeds to 1500 °C, the oxygen groups and crosslinking C–C band could induce graphitization of cellulose-based carbon on the condition of enough active energy. Moreover, it is clarified the configuration stability, adsorption capability, and electronic properties of different oxygen groups incorporated the theoretical calculations. Besides, the aggregated structure of cellulose has a powerful influence on structure of cellulose-based carbons. The celluloses extracted from wood, cotton, and bamboo-based plant by chemical pretreatment is cellulose II type, and bacterial cellulose belongs to cellulose I type. As previously mentioned, cellulose I type has two exiting forms of *I*_*α*_ and *I*_*β*_. At 55 °C, the number of hydrogen bonding O_3_–H_5_···O_5_ of cellulose *I*_*α*_ and *I*_*β*_ reduces, and the hydrogen bonds appear in increasing of cellulose *I*_*α*_ and *I*_*β*_ because of O_2_–H_2_···O_6_ conversion [77]. Because the formation of a new intermolecular hydrogen bonding, the cellulose-based carbon materials have the stable hydrogen bonding network even at high temperature pyrolysis.

**Fig. 5:**
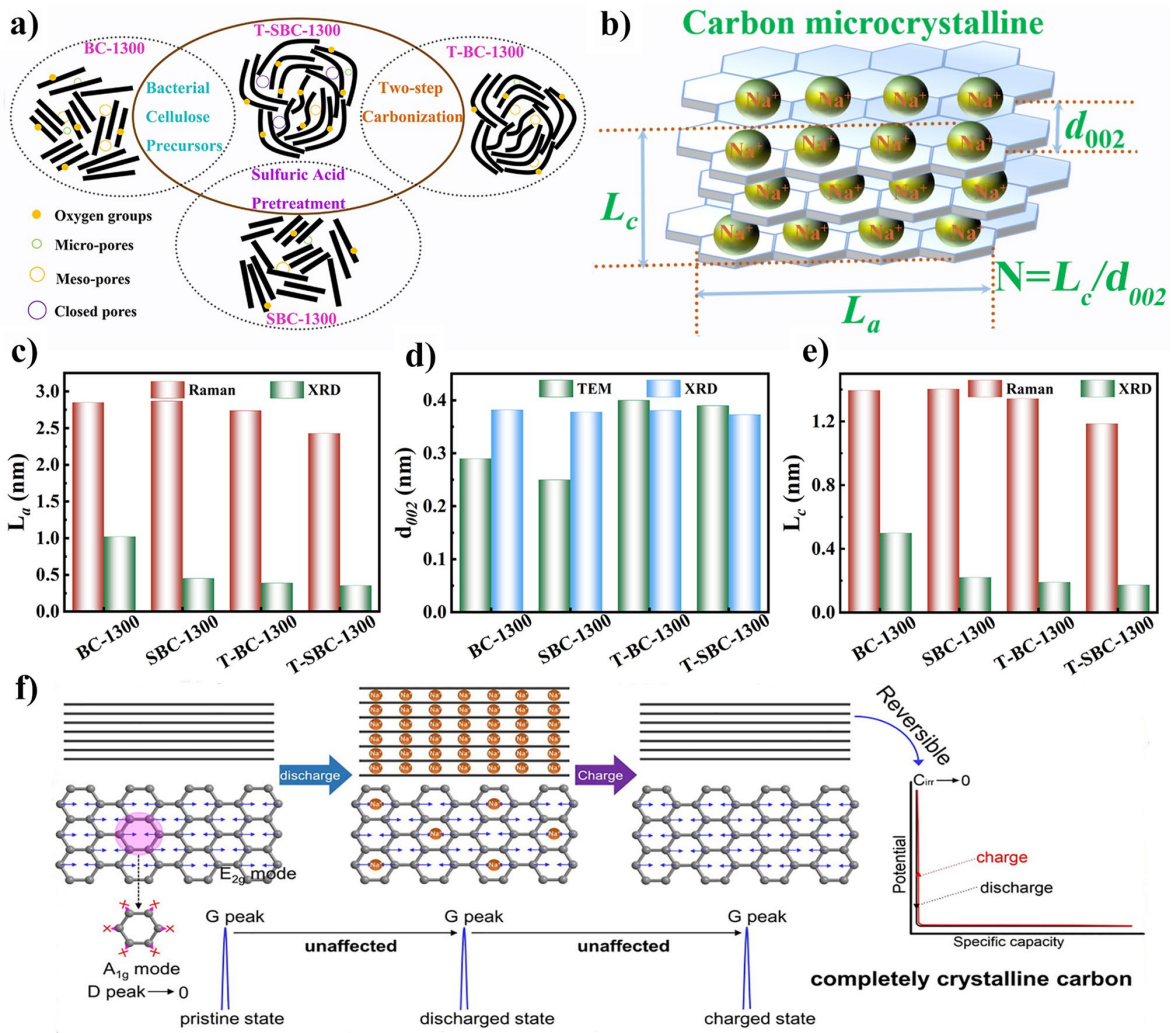
The cellulose-based carbon materials’ structure: **a** microcrystalline morphology [82]; **b** the structure parameters of a carbon microcrystalline cell; **c**–**e** the values of *L*_a_, *d*_002_, and *L*_c_ calculated by characterization methods in our previous work [82]; **f** the sodium storage mechanism for complete crystalline carbon [83]. **a** and **c**–**e**, adapted with permission [82], Copyright 2022, Elsevier Limited. **f** adapted with permission [83]. Copyright 2022, Elsevier Limited

For the pyrolytic parameters, the carbonization temperature, heating speed, carbonized atmosphere, and flow speed have critical influences on the structure of cellulose-derived carbon materials. Cotton-based cellulose own natural twist and hollow micro-structure. With the carbonization temperature increasing, the micro-structure of cotton occurs collapse and/or rearrangement to form a higher graphitized carbon material. The other cotton-based materials such as catkins [78] and kapok [79], maintain the similar structures after carbonization [80, 81]. In our previous work, we selected two-step carbonization method to prepare cellulose-based carbon materials [82]. Compared with the one-step carbonization of cellulose precursor, the two-step carbonization could achieve the carbon structure with completer and stabler. Moreover, the majority of the carbon microcrystalline structure obtained during the carbonization process is characterized by closed pores resulting from the transformation of open nanopores and ordered carbon crystallites into enclosed voids surrounded by short-range carbon structures (Fig. 5a).

Carbon microcrystalline structure with more graphitic nanodomains can provide effective electrons transfer pathways and stable sodium ions storage behaviors. It is worth noting that the carbon microcrystalline structure, that mainly involves the length $L_{{\text{a}}}$, thickness ${L}_{{\text{c}}}$, stack layers *N*, interlayer distance ${d}_{002}$, and graphited degree${I}_{{\text{D}}}/{I}_{{\text{G}}}$, plays an important role in regulating structure of cellulose-based carbon materials (Fig. 5b). The feature of an ideal carbon anode is the shorter ${L}_{{\text{a}}}$, thinner ${L}_{{\text{c}}}$, less stack layer, higher interlayer distance, and suitable graphited degree ${I}_{{\text{D}}}/{I}_{{\text{G}}}$, which favor for upgrading ion diffusion pathways, declining the ion diffusion barrier, and offering the more active sites to ion transport [84, 85]. In a previous work from our group, cellulosed-based carbon with appropriate microcrystalline structure parameters was prepared by tailoring the structure of precursor through pretreatment [82]. The cellulose-based carbon materials show the related structure parameters value of ${d}_{002}$ = 0.39 nm, ${L}_{{\text{a}}}$ = 0.36 nm, ${L}_{{\text{c}}}$ = 0.17 nm, and ${I}_{{\text{D}}}/{I}_{{\text{G}}}$ = 1.81. From crystal face size, the difference in crystal cell structure parameters lies in the carbon layer spacing from 0.33 to 0.37 nm, the thinner thickness and shorter length of the carbon layer and the number of stacked layers. Figure 5c–e shows the values of *L*_a_, *d*_002_, and *L*_c_ of cellulose-based carbon materials with different pretreatment methods. Obviously, the acquisition of these three values is contingent upon the different methods of calculation.

The size and orientation of the graphitic crystallites strongly influences carbon properties and defines the texture, porosity, surface-area, capacitance and electrical conductivity [86]. The pore formation and crystal cell size are closely related to pyrolysis process. Some researchers propose that carbon with complete crystallinity, containing graphite-like crystals and serving as sodium ion anode materials, exhibits exceptional electrochemical performance [83]. They think that the sodium storage in fully crystalline carbon is solely attributed to reversible intercalation/de-intercalation of Na^+^ within perfectly aligned graphene-like layers, rather than absorption at defects, heteroatoms, and functional groups (Fig. 5f). The proximity of the *p*-band center of carbon to the adsorption site is crucial for both *E*_a_ (adsorption energy) and *E*_b_ (diffusion barrier energies). A weaker (stronger) adsorption and a smaller (larger) diffusion barrier are observed when the *p*-band center is farther (closer) from the Fermi level [84]. The microcrystalline structure of carbon also affects the adsorption and diffusion barriers energy of Na^+^. Specifically, the distance between carbon layers () has a significant impact on the adsorption energy of Na^+^ ions, while its effect on diffusion barriers energy is weak. Additionally, shorter graphite layer structures with enhanced *p*-band centers and introduced vacancy defects and bulk oxygen tend to enhance the adsorption of Na^+^ ions.

## Relationship of Sodium Storage and Rate Performance in Carbon Anodes

Rate performance is an indicator to evaluate the discharging/charging speed of electrode materials at a given current density that is a key parameter to enhance power and energy density [87]. It is well-known that sodium-ion batteries have been limited for large-scale application due to their low power density. At present, the rate capacity of some cellulose-derived carbon materials can be about 300–390 and 236–280 mAh g^−1^ at the current densities 0.05 and 1 A g^−1^ [88–91]. In higher current densities of 2–10 A g^−1^, the rate capacity is in the range from 130 to 206 mAh g^−1^ [66, 92]. This is obviously not adequate for developing high power-energy density of SIBs.

In general, rate performance is characterised with two methods of galvanostatic charging/discharging (GCD measure and semi-empirical equation [93, 94]. The former measure is often used to obtain the changing capacity with different current density, while the latter method accurately shows the rate dependence of electrode capacity in views of electrode properties, via the feature time linking to charging/discharging process. At present, GCD curves can obviously obtain the decreasing specific capacity with the increasing current density, but it cannot clearly explain for influence factors that limit the rate performance. Several models have been proposed, and tend to think ion diffusion between electrolyte and electrode materials is the main factor limiting rate performance based on ion transfer time constant [95, 96]. Coleman et al. [93] proposed the semi-empirical equation, which fit the relationship of capacity and rate data to assess the rate performance. The semi-empirical equation is as follows:

1 $$\frac{Q}{M} = Q_{{\text{M}}} \left[ {1 - (R\tau )^{{\text{n}}} \left( {1 - e^{{ - (R\tau )^{ - n} }} } \right)} \right]$$

In Eq. (1), fitting rate and *Q*/*M* data can get three parameters (*Q*_M_, *τ*, *n*) to quantify rate performance, where *Q*/*M* is the measured specific capacity (mA h g^−1^, generally normalized to active mass), and can substitute *Q*/*A* (area capacity) and *Q/V* (volumetric capacity). While *R* is the rate defined via the specific current (*I*/*M*) as *R* = (*I*/*M*)/(*Q*/*M*), where *Q*_M_ is the low-rate specific capacity and *τ* is the characteristic time associated with charging/discharging data [93, 97].

**Fig. 6:**
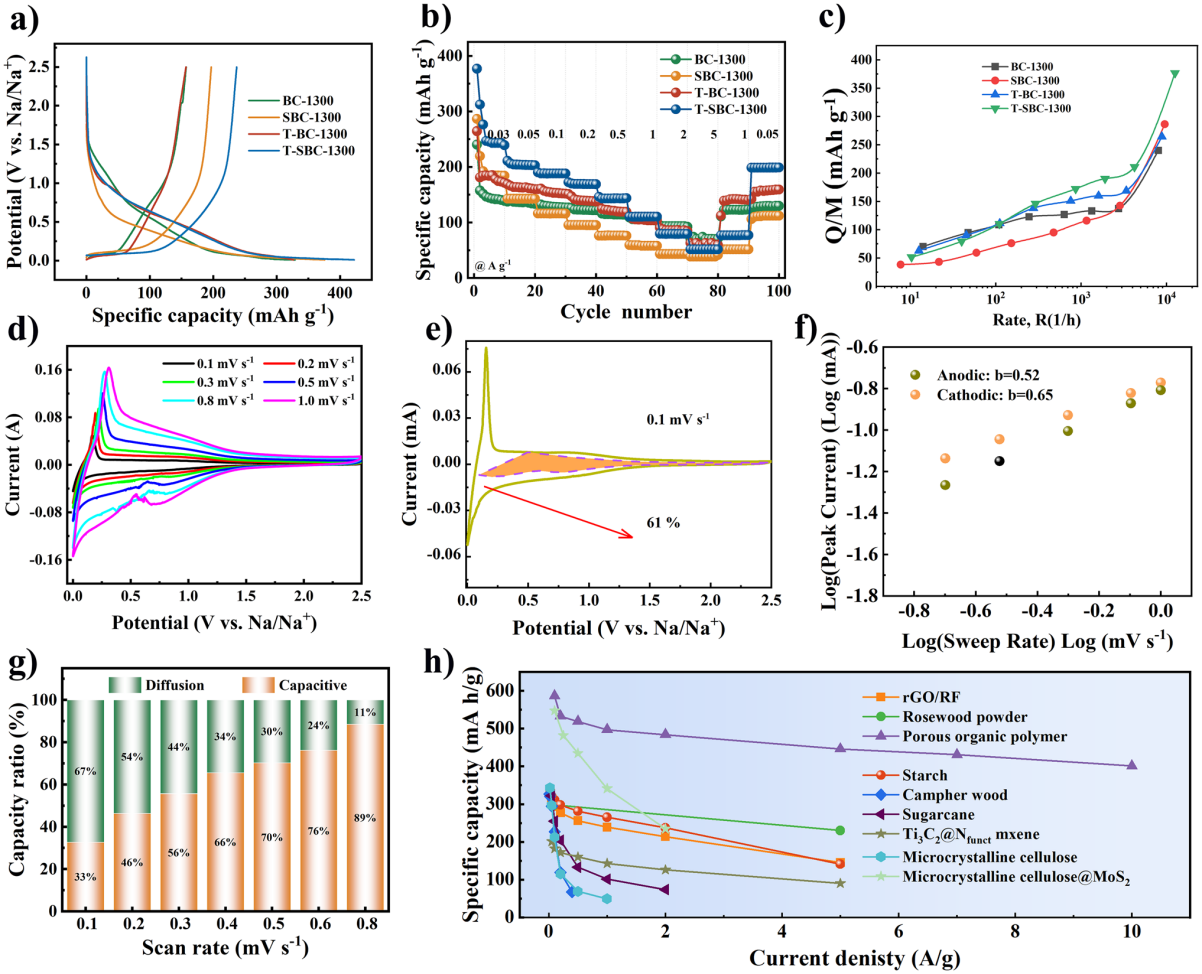
The relationship of sodium storage and rate performance for cellulose-based carbon in our previous work: **a** GCD curves; **b** rate capacity at different current densities [82]; **c** the fitted curves of capacity versus rate data with semi-empirical equation for batteries electrodes; **d** CV curves at different sweeps; **e** capacity ratios at sweep speed 0.1 mV s^−1^; **f** the *b* value & peak current under different sweep rates [76]; **g** capacity ratios at different sweep rates; and **h** the comparing rate capacity in the different cellulose-derived hard carbon materials. **a**, **b** Adapted with permission[82], Copyright 2022, Elsevier Limited. **d**–**f** Adapted with permission [76], Copyright 2022, the Royal Society of Chemistry

Taking a series of bacterial cellulose-derived carbons as an example to illustrate rate performance. Figure 6a, b shows that the electrochemical performance of cellulose-derived carbon anode materials obtained by liquid (SBC-1300) and vapor phase (T-BC-1300) modified cellulose precursors [82]. The GCD curves of Fig. 6a exhibit the discharging capacity of four prepared samples. T-SBC-1300, that suffer from liquid and vapor phase treatment, indicates higher capacity (420.6 mAh g^−1^ at 30 mA g^−1^) than that of other BC-based carbons. The rate performance of these carbons can be seen in Fig. 6b that all display good capacity reversibility when the current density back to 50 mA g^−1^. According to above-mentioned semi-empirical equation, the fitting curves are shown in Fig. 6c, that describe the relationship between electrode capacity and rate; and it is dependent on the diffusion time of ions in the electrode material. It is well-known that the total capacity decreases with current density increasing. That is because the current density of electrons is more than that of ions in the interface between the electrolyte and electrode, resulting the polarization and metallic plating is more likely to happen at high current density.

The total capacity of anode is sum of plateau capacity and slope capacity. In the typical discharging/charging curve, at 0.1 V position is a cut-off point to divide slope (above 0.1 V) and plateau (below 0.1 V) regions. There also is different plateau voltage, like 0.25 V [98] and 1.2 V [63], because some hard carbons have unique microstructure or heteroatomic doping. Based on the “card house” model proposed by Dahn, different views on the specific sodium storage mechanism of hard carbon have emerged one after another. It mainly contains “insertion-filling” [99], “adsorption-insertion” [100], “adsorption-insertion-filling” [101], “adsorption-filling-insertion-deposition” [102], “extended adsorption-insertion” [9], and “adsorption-filling” [50] models. Therefore, three distinct sodium storage mechanisms observed in hard carbons: intercalation within graphite layers, adsorption at surface edges and defects, and filling of nanopores. These various statements about storage sodium mechanisms are closely related to the hard carbon’s precursors.

All these different mechanisms can be subsumed under two broad categories, including the diffusion-controlled intercalation behavior and surface induced capacitive behavior. At present, it is widely acknowledged that hard carbon consists of randomly oriented graphitic layers and a wide range sized voids and pores, forming a turbostratic structure with large interlayer distance [81, 103]. Based on the structural features, the storage sodium ions behavior includes Na^+^ insertion/extraction from microcrystalline graphite interlayer, Na^+^ adsorption on edge defect, vacancy and surface function groups, and Na^+^ filling pore structure with nanoclusters. Na^+^ ions are adsorbed onto defect/edge sites and partial filled in micropore in the slope region above 0.1 V. The storage sodium behaviors of adsorption at edge and defects supply pathways for sodium mobility and sodium storage active sites. The enhancing surface-controlled capacitive is a viable strategy for improving rate capability of carbon-based materials. In the plateau region below 0.1 V, Na^+^ ions intercalate into the graphitic layers while additional adsorption takes place near the cutoff potential within the micropores. Meanwhile, sodium cluster arises from the high concentration of Na^+^ ions present in these micropores below 0.1 V.

The quantitative analysis of capacity-behavior and diffusion control behaviors is proposed by Conway [104–106]. According to the equations of $I(v) = av^{b}$ and $I(v) = k_{1} v + k_{2} v^{1/2}$, the proportion of capacitive and diffusive capacity in anode materials at specific current density is calculated [63, 76]. It is noted that the range of *b* value determines the storage behavior of sodium ions. Here, the scale of *b* value is 0.5–1; when *b* value is 0.5 means that storage sodium behavior is dominated by diffusion control, when *b* value is 1 means that it shows capacitive storage sodium behavior, while when *b* value is between 0.5 and 1 means that two above-mentioned behaviors are co-existence. The values of *k*_1_ and *k*_2_ can be calculated by fitting the plots of ${{i(v)} \mathord{\left/ {\vphantom {{i(v)} {v^{1/2} }}} \right. \kern-0pt} {v^{1/2} }}$ as a function of $v^{1/2}$. Besides, $k_{1} v^{1/2}$ and $k_{2} v$ represent diffusion-controlled intercalation behavior and surface induced capacitive behavior at a fixed voltage. Based on these, the rate performance of Bacterial cellulose-derived hard carbon is characterized in our previous studies, as shown in Fig. 6d–g.

Given that cellulose-derived carbon materials based the storage sodium mechanism illustrated above, the favorable structure of cellulose-derived carbon includes short range order and suitable carbon layers distance (0.36–0.40 nm) [107, 108], internal micropores (or voids between graphitic domains) [109, 110]. Figure 6h exhibits a big difference in rate capacity caused by different microstructure parameters of carbons derived from various precursors. The connection between scan rates and normalized capacity may diagnose ion-diffusion process whether rate-limiting step [89]. Wang et al. [111] used hemp haulm to synthesize three-dimensional free-standing hard carbon, that exhibits the plateau capacity of 140 mAh g^−1^ through reconstructing carbon surface at high current charging/discharging process in favor of sodium ions insertion. In addition, Chen et al. developed hard carbon via starches fermentation and carbonization, where plateau capacity is contributed to pore filling [112]. Zhou et al. [113] prepared porous carbon derived from rosewood with rich closed pore and proposed the plateau capacity of 189 mAh g^−1^ based pore filling mechanism.

In our opinions, the plateau capacity is very important, even a limiting factor for improving the rate performance of SIBs [84]. In particular, the charging capacity of half-cell at below 0.1 V vs. Na/Na^+^ is considered a standard to certify practical capacity of anode. The two types of higher plateau (0.4–0.7 V) and lower plateau (below 0.1 V) are divided [114]. The higher voltage capacity corresponding to oxygen function groups of carbon surface boost the capacitive storage, and lower voltage capacity through sodium ions insertion. A higher charge potential would reduce work voltage window of practical full cell. The increasing reversible capacity below 1.0 V vs. Na/Na^+^ is an effective method to increase rate capacity. In general, the microstructure parameters of hard carbon, such as *d*_002_, *L*_a_, *L*_c_, defect degree (*I*_D_/*I*_G_, and porous distribution have much influence on the plateau capacity of anode materials [115–117].

Recently, developing fast-charging batteries is a hot-point, to reduce charging time consuming by accelerating reaction kinetics in batteries [118]. The achieving of high-power and fast-charging need anode materials with excellent rate performance. Heubner et al. [119] used experimental procedure based on current controlled EIS with varying amplitudes to verify in terms of applicability of the Butlere-Volmer equation for charge transfer kinetics. The Butlere-Volmer equation describes the relationship between the activation overpotential and the resulting current density. The equation is as follows [119]:

2 $$j = j_{0} \left( {\exp \left[ {\frac{{\alpha_{{\text{a}}} zF}}{RT}\eta_{{{\text{ct}}}} } \right] - \exp \left[ {\frac{{\alpha_{{\text{x}}} zF}}{RT}\eta_{{{\text{ct}}}} } \right]} \right)$$

where *j*_0_ is the exchange current density and *η*_ct_ is the activation overpotential, and *z*, *F*, *R*, and *T* are the valance, Faraday’s constant, the universal gas constant, and the absolute temperature. The anodic charge transfer coefficient *α*_a_ is set to 0.5 most of the time in battery modeling and simulation assuming *α*_a_ + *α*_c_ = 1.

Both the charge transport and ion diffusion are limited in terms of quantity and quality at high current densities, in which the quantity refers to the transport/diffusion total number of charge and ions and the quality represents the transport rate and diffusion accessible path/space of ion and charge [120]. Generally speaking, electron transfer need higher order and ion diffusion need more space [121]. There are so much strategies on improving the charge transport and ion diffusion by modifying microstructure [122–124], introducing defects and vacancy, designing pore structure, regulating graphite degree, and optimized precursors [125, 126] at cellulose-based carbon materials level. Unfortunately, a serious of measures are accompanied by low initial coulombic efficiency (ICE [5] and reducing plateau capacity [127]. Therefore, it is important to find the equilibrium relationship between ion diffusion and electron transfer in order to enhance rate performance of SIBs.

## Limitations of Cellulose-Derived Carbon Materials at Rate Performance Level

Rate performance for SIBs is actually closely related to power density. Nowadays, the popularization of high-power equipment and portable equipment is becoming more and more important for the rate performance. Currently, the cellulose-based hard carbon materials are a good candidate for SIBs anode, but its rate is really unsatisfied the requirements of higher power density of SIBs. Table 3 illustrates the electrochemical performance of cellulose-based carbon materials as SIBs anode. Good electrode materials need high reversible storage capacity and rapid ions and electrons transport [114, 128]. However, natural properties of cellulose-based materials bring some roadblocks in carbonization process. To our knowledge, the electronic conductivity and sodium ion diffusion coefficient of cellulose-based hard carbon are ~ 10^2^ S cm^−1^ [129] and ~ 10^–9^–10^–13^ cm^2^ s^−1^ [84], respectively, that are much smaller than that of alloy anode materials (such as Na_3_Sb and Na_15_Sn_4_) of ~ 10^4^ S cm^−1^ [130].

**Table 3:** Comparison of diffusion coefficients, capacity, and ion diffusion lengths of different cellulose-derived carbon materials

Precursors	*T* (℃)	Charge transfer impedance (Ω)	Ion diffusion coefficient (cm^2^ s^−1^)	Ion diffusion length (mm)	Rate performance		References
					Current density (A g^−1^)	Capacity (mAh g^−1^)	
Bacterial cellulose	1050	–	3.5 × 10^–12^	5.25 × 10^–8^	0.03	222.9	[76]
					1	68.9	
Cotton	1300	–	1.35 × 10^–9^	2.43 × 10^–5^	0.015	275	[81]
					0.3	180	
Bacterial cellulose	800	325.7	–	–	0.05	355	[63]
					10	255	
Fire wood	1300	26.6	4.2 × 10^–8^	2.52 × 10^–4^	0.05	276	[98]
					1	108	
Bacterial cellulose	1300	270*	–	–	0.2	271	[89]
					20	128	
Cellulose	800	454.6	4.77 × 10^–10^	2.86 × 10^–6^	0.02	308	[131]
					1	120	
Cellulose nanocrystals/polyethylene oxide	1300	80	3.0 × 10^–10^	5.4 × 10^–6^	0.03	290	[132]
					0.6	105	
Oxide bacterial cellulose	1000	250*	–	–	0.05	276	[133]
					5	81	
Microcrystalline cellulose	1000	107.2	–	–	0.05	293.5	[70]
					2	86.1	
Kraft Lignin/Cellulose Acetate	1000	180	–	–	0.05	326	[67]
					0.5	143	
Cellulose nanocrystals	1000	–	–	–	0.02	375	[72]
					0.2	220	
Filter/pitch	1000	–	2.2 × 10^–10^	3.96 × 10^–5^	0.03	282	[134]
					1.2	92*	
rGO/CNF	300	48	–	–	0.02	320	[135]
					3.2	100	
Lotus leaves	800	10.2	1.2 × 10^–9^*	2.16 × 10^–5^	0.04	250	[136]
					2.0	78	
Cellulose	1300	–	–	–	0.025	353	[16]
					2.0	240*	
Microcrystalline cellulose	1200	200	–	–	0.02	302	[137]
					0.5	261	
Bass-wood	500	35.78	2 × 10^–12^	2.4 × 10^–8^	0.1	371.4	
					2.0	187.4	[138]

The * refers estimated value according to the related literature

The key parameters for high-rate capacity of cellulose-based materials are the electron conductivity and ion diffusion. The former is the number of *sp*^3^ hybrid orbitals between two carbon atoms, and the latter is caused by disorder structure of cellulose with various directions [139]. To some extent, SIBs can be polarized at high current rate, resulting the potential safety and low kinetic problems. The poor rate capacity of hard carbon in half-cells is attributed to the low-potential plateau behavior of hard carbon and the polarized Na counter electrode at high rates [140]. Defects, nanopores, and microcrystals are the three crucial structural parameters determining the performance of sodium storage in cellulose-derived carbon. The interaction between the defective carbon layer and sodium ions significantly influences the charge storage state of sodium ions in cellulose-derived carbon, meanwhile the size distribution of nanopores affects the characteristics of clusters formed by sodium within these pores. The order structure of cellulose-based materials provides more electron percolation paths in the bulk volume and reduces near surface resistance [141]. Therefore, meticulous attention should be given to the design of cellulose-based materials and cellulose-derived carbon on building robust charge transport network and optimizing valid ion transport paths.

### Electronic Conductivity of Cellulose-Derived Carbon Materials

Electronic conductivity is a behavior happening in internal transportation of electrode materials and interface between current collector and electrode. The electronic conductivity closely relates to the charge-transfer resistance, such as specific resistance of electrode, contact resistance between electrode and electrolyte, ion-diffusion resistance in the bulk electrolyte [120]. The charge transfer during electrochemical intercalation is commonly understood as the movement of ions across the interface between the material and electrolyte. It is often assumed that the activation energy associated with this step determines the rate capability of batteries based on intercalation [142]. Generally speaking, electrode resistance is smaller, and electric conductivity is larger. Cellulose-based carbon exhibits lower electronic conductivity due to its disorder structure. The enhanced electric conductivity is an important approach to boost the Na^+^ storage reversibility and rate-performance by accelerating the charge transfer kinetics. Besides, the interfacial ion conduction would be facilitated in the reduced space‐charge regions, and the less accumulated electrons at interfacial anode could decrease the SEI‐induced interfacial resistance.

In addition, electrical conductivity depends on both the concentration and mobility of charge carriers. According to energy band theory, electrons of valence band across the forbidden band and enter to conduction band. Here, the relationship between carrier concentration and electronic conductivity follows the below equation [143–145]:

3 $$\sigma = n|e|\mu_{{\text{e}}}$$

where *σ* is the electronic conductivity, *n* is the carrier concentration, and *μ*_e_ is the carrier mobility. *|e|* is charge absolute value.

There are holes and free electrons in anode materials, and *σ* could be further described as shown in the following equation [144]:

4 $$\sigma = n_{{\text{i}}} e\mu_{{\text{e}}} + p_{{\text{i}}} e\mu_{{\text{h}}}$$

where *n*_i_ and *p*_i_ are the concentrations of electrons and holes, respectively, *μ*_e_ and *μ*_h_ represent the mobility of the electrons and the holes, respectively. Based on Eq. (4), the increased *n*_i_ and *p*_i_ are beneficial to enhance the conductivity of anode materials. For example, doping heteroatoms could offer more electrons or vacancy and strength electronic local structure in the manner of introducing non-intrinsic carriers, that benefit for increasing electron conductivity of carbon materials [63, 66, 80]. Of course, adding conductivity agent or coating could also boost electrons diffusion of electrode materials by introduced intrinsic carriers to achieve improved conductivity.

It is well-known that cellulose is not inherently electrically conductive. Therefore, the cellulose-based carbon materials only have a good electric property by carbonization at higher temperature. With the temperature increasing, the proportion of C–C of *sp*^2^ and *sp*^3^ hybrid orbitals can change, and form the unregular and disorder microcrystalline carbon structure. That determines on the electric property of cellulose-derived carbon structure [13]. Here, *sp*^2^ orbitals carbon generally represents honeycomb graphene layer, and *sp*^3^ orbitals carbon refers defect areas. Yu’s group [146] prepared cellulose-based aerogels via pyrolysis at 1300 °C that exhibits good electrical conductivity of 0.35 S cm^−1^. In comparison to commercial graphite with a conductivity of 6 × 10^4^ S m^−1^, it is evident that the cellulose-derived carbon obtained through direct carbonization exhibits certain limitations.

In general, the graphitization degree increases and the conductivity becomes better with an enhancement of carbonized temperature. However, the improvement of graphitization degree results in more order carbon microcrystalline structure, and less interlayer distance, and defects, which is unfavorable to the improving plateau capacity. Besides, both inter- and intra-molecular strong hydrogen bonds of the cellulose chain bring some difficulties for further processing of cellulose. On the one hand, the inter- and intra-molecular strong hydrogen bonding limited the carbon layer rearrangement, ascribing to molecular chain cannot extend. A significant number of crystalline regions occurs directly crosslinking to form carbon microcrystalline structure, to some extent limiting optimization of crystal cell structure parameters during carbonized process. On the other hand, intermolecular chains have strong hydrogen bonds lead to the harder modification and more complex preparation process. It is difficult to adjust its crystal structure, thus affecting the optimization of carbon microcrystals, further to limit the improving for conductivity. Bacterial cellulose, methyl cellulose, hydroxypropyl cellulose and carboxymethyl cellulose were in situ polymerized with aniline and obtained the four conductive cellulose fabrics. The electrical conductivity value of the four fabrics were 1.990 × 10^–2^, 2.840 × 10^–2^, 2.080 × 10^–2^, and 0.962 × 10^–2^ S cm^−1^, respectively [147]. Therefore, cellulose type has influence on the electrical conductivity of cellulose.

### Ion Diffusivity of Cellulose-Derived Carbon Materials

The ion diffusion in anode materials as an essential parameter to estimate rate performance and power density of metal-ion batteries, and expressed as diffusion coefficient (*D*_i_. The ions diffusion behavior of carbon electrode is evaluated by performing coulometric titration measurement, electrochemical impedance spectra (EIS), cyclic voltammetry (CV), and galvanostatic intermittent titration technique (GITT). Both EIS and GITT methods can obtain the ion diffusion in electrode materials, and the diffusion behaviors can be observed from the spectrum at low-frequency region of EIS. However, all these calculation methods are suitable to qualitative analysis due to uncertainty electrode area. Note that the diffusion coefficient calculated by above methods is apparent coefficient. It actually represents that the ion diffusion pathway between the solid-state anode materials and liquid electrolyte.

In here, ion diffusion can be calculated by coulometric titration technique [148] and GITT method [132] as follows:

5 $$D_{{\text{i}}} = \frac{4}{\pi }\left( {\frac{{V_{{\text{M}}} }}{{SF_{{z_{i} }} }}} \right)^{2} \left[ {I_{0} \left( {\frac{{{\text{d}}E}}{{{\text{d}}\delta }}} \right)/\left( {\frac{{{\text{d}}E}}{{{\text{d}}\sqrt t }}} \right)} \right]^{2}$$

6 $$D_{{\text{i}}} = \frac{4}{\pi \tau }\left( {\frac{{m_{{\text{B}}} V_{{\text{M}}} }}{{M_{{\text{B}}} S}}} \right)^{2} \left( {\frac{{\Delta E_{{\text{S}}} }}{{\Delta E_{\tau } }}} \right)^{2}$$

In Eq. (5), d_E_/d_*δ*_ is the slope of the coulometric titration curve, *V*_M_ is the molar volume of the sample, *I*_0_ is the constant current, and *F* is Faraday’s constant, and d*E*/d*t*^1/2^ is the short-time instantaneous voltage change slope. In Eq. (6), *τ* is for the pulse duration, *m*_B_ (g and *M*_B_ are the active mass and molar mass of carbon, *V*_M_ is the molar volume, and S (cm^2^) is the active area of the electrodes. The parameters of *∆E*_S_ and *∆E*_*τ*_ are the potential changes at τ and relaxation stages per GITT step. It should be guaranteed the time *τ* of the current flux is small compared to *L*^2^/*D*_i_. Just as importantly, the CV and EIS [149, 150] are used by some researchers, and the detailed process is calculated as shown follow:

7 $$i_{{\text{p}}} = \left( {2.69 \times 10^{5} } \right)n^{3/2} AD_{{\text{i}}}^{1/2} v^{1/2} C$$

8 $$i_{{\text{p}}} = \left( {2.99 \times 10^{5} } \right)n\left( {\alpha n_{\alpha } } \right)^{1/2} ACD_{{\text{i}}}^{1/2} v^{1/2}$$

9 $$j_{0} = \frac{{i_{0} }}{A} = \frac{RT}{{nFR_{{{\text{ct}}}} A}}$$

10 $$D_{{\text{i}}} = \frac{{R^{2} T^{2} }}{{2A^{2} n^{4} F^{4} C^{2} \sigma^{2} }}$$

11 $$Z^{\prime } = R_{{\text{s}}} + R_{{{\text{ct}}}} + \sigma w^{ - 1/2}$$

In Eqs. (7) and (8), *i*_p_ is the current, *n* is the number of transferred electrons, *A* is the electrode area, *C* is the metal ion concentration, *D*_i_ is the diffusion coefficient, *v* is the scan rate of cyclic voltammetry, *σ* is the exchange coefficient for a completely irreversible reaction *σ* = 0.5, and *n*_*α*_ is the number of reaction electrons in the rate-controlling step (*n*_*α*_ = *n* when approximating the treatment). In Eqs. (9–11), *j*_0_ refers exchange current density, the σ and *w* are the Warburg coefficient and angular frequency, *A* is the electrode surface area, *n* is transferred electrons numbers, *F* is the Faraday constant, *C* is the concentration of Na^+^, *T* is the absolute temperature, and *R* is the gas constant. According to Eq. (10), it could be seen that the lower the value of *σ* (Warburg impedance coefficient), the higher diffusion coefficient. The value of *σ* is calculated by the linear fitting slope [17].

The lattices of cellulose-derived carbon materials change with different synthesis methods that affects the migrating ions. The understanding and optimizing ion mobility for the development of advanced and efficient battery materials is very important [151]. A shortened ion diffusion path facilitates the enhanced mobility of ions within both the electrode and electrolyte, thereby resulting in an elevated ion diffusion coefficient. Through considering factors such as ion diffusion coefficients, capacity, and ion diffusion lengths, the rate performance of carbon materials would be improved. The rate of ion diffusion is highly dependent on the diffusion coefficient (*D*_i_ and the length (*λ*) of materials, which can be mathematically expressed using Einstein's formula [120]:

12 $$\tau = \frac{\lambda }{{D_{{\text{i}}} }}$$

where *τ* is the diffusion time; *λ* is the diffusion length, which is up to the geometric size of the active material; *D*_i_ is the diffusion coefficient depending on the architecture of the active material, which is increased by designing the most appropriate architecture providing spacious diffusion pathways. Undoubtedly, the diffusion kinetics (the migration rate, *τ*) relies on the crucial factors of ionic diffusivity and diffusion length. Hence, it is imperative to enhance ion diffusivity (*D*_i_ in both bulk and surface domains while simultaneously reducing diffusion length (*λ*). In this regard, nanoscale materials have gained significant popularity owing to their small particle size, which effectively narrows down the diffusion length (*λ*) and enhances charge transfer efficiency [152].

According the paths of ions transportation, pore structure is the most important parameters should be taken account for improving rate performance of SIBs. Generally, cellulose-based materials with three-dimensional network and rich oxygen groups are beneficial to produce cellulose-derived carbon materials with abundant porous structure. However, the interconnected intermediate state forms due to the breaking of intermolecular chains bonds, resulting the porosity decreases in the carbonized process. Besides, the cellulose-based carbon materials are easier to form stacking carbon sheets due to hydrogen bonds and Van der Waals’ force of cellulose chains. This structure is unfavorable for enlarging the distance of carbon layers, inhibiting sodium ion diffusion kinetics in the carbon derived from cellulose-based materials.

Moreover, there are types of pores occurring in the carbon materials, such as closed pore, open pore, micropore, mesopore, and so on. These pores have a different effect on improving ions diffusion. For examples, open pores are conducive to stabilize carbon structure at discharging/charging process. The rich accessible closed pores exhibit good diffusion ability [153] that is helpful to improve plateau capacity by expanding pores filling. In addition, the ultra-micropore is deemed to hinder contact between carbon surface and liquid electrolyte, which can achieve high rate performance [114]. Our group has done a lot of work on controlling pore structure of cellulose-based carbon materials for SIBs anode [76, 82, 133]. However, the high crosslinking density of cellulose makes the degradation and rearrangement of carbon framework more difficulties, even at the higher carbonized temperature [153]. Especially for the carbon derived from natural cellulose materials with high crystalline degree, it has not enough defects and vacancy sites to support ion diffusion in carbon interlayers.

A whole electrode includes current collector, active materials, conductive agents and binders, and its ion transport is influenced by the electrode architectures, such as porosity, conductivity, tortuosity, and spatial heterogeneity [154]. The limited rate capability is often attributed to solid-state diffusion limitations. Commercial electrode typically possess a thickness ranging from 50 to 100 μm and a areal mass loading about 10 mg cm^−2^ [155]. The sluggish kinetics of ion transport, resulting from the enhanced active materials loading, hinder ions penetrate into the active sites within the electrode material matrix. The compaction density influences the electrochemical reaction induced by electrolyte diffusion. Solid-state electrolytes are highly anticipated in alkali metal batteries due to their exceptional ionic conductivity, wide electrochemical stability window, low electrical conductivity, and remarkable chemical stability. However, one major challenge lies in achieving superior interfacial contact between the solid-state electrolytes and solid electrodes because affected electrics/ions transport [156]. The electrode’s stability must be considered to improve the battery’s rate performance, as exposure to air leads to reactions between water, oxygen, and carbon dioxide with the electrode components [157]. In the past decades, nanoengineering has been extensively employed to reduce the solid-state diffusion length and enable both rate performance and capacity utilization.

### Solid Electrolyte Interphase on Cellulose-Derived Carbon Materials

Both ions diffusion and charge transport are passing through interface between cathode/anode and electrolyte. Thus, the profound impact of “external” factor– solid electrolyte interphase (SEI on the functionality of electrodes attracts widely attention) [158–160]. Due to the solvation ability of polar solvents in electrolytes, the cations readily coordinate with the solvent, leading to the formation of diverse solvation configurations. Upon reaching the electrode surface, these solvated cations undergo de-solvation and decompose their solvation shells to generate a SEI layer. The transfer impedance of Na^+^ at the electrode/electrolyte interface is reduced by maintaining a low ion-electrolyte bonding energy during the insertion/extraction process of Na^+^ [161]. The surface free energy of carbon materials is determined by factors including the distribution of carbon atoms in inner layers and surface edges, polar functional groups, heteroatom doping, and defects on surfaces and pores of carbon materials. These factors collectively limit the stability, robustness, and integrity of the SEI layer formed on carbon electrodes while also restricting improvements in rate performance for SIBs.

**Fig. 7:**
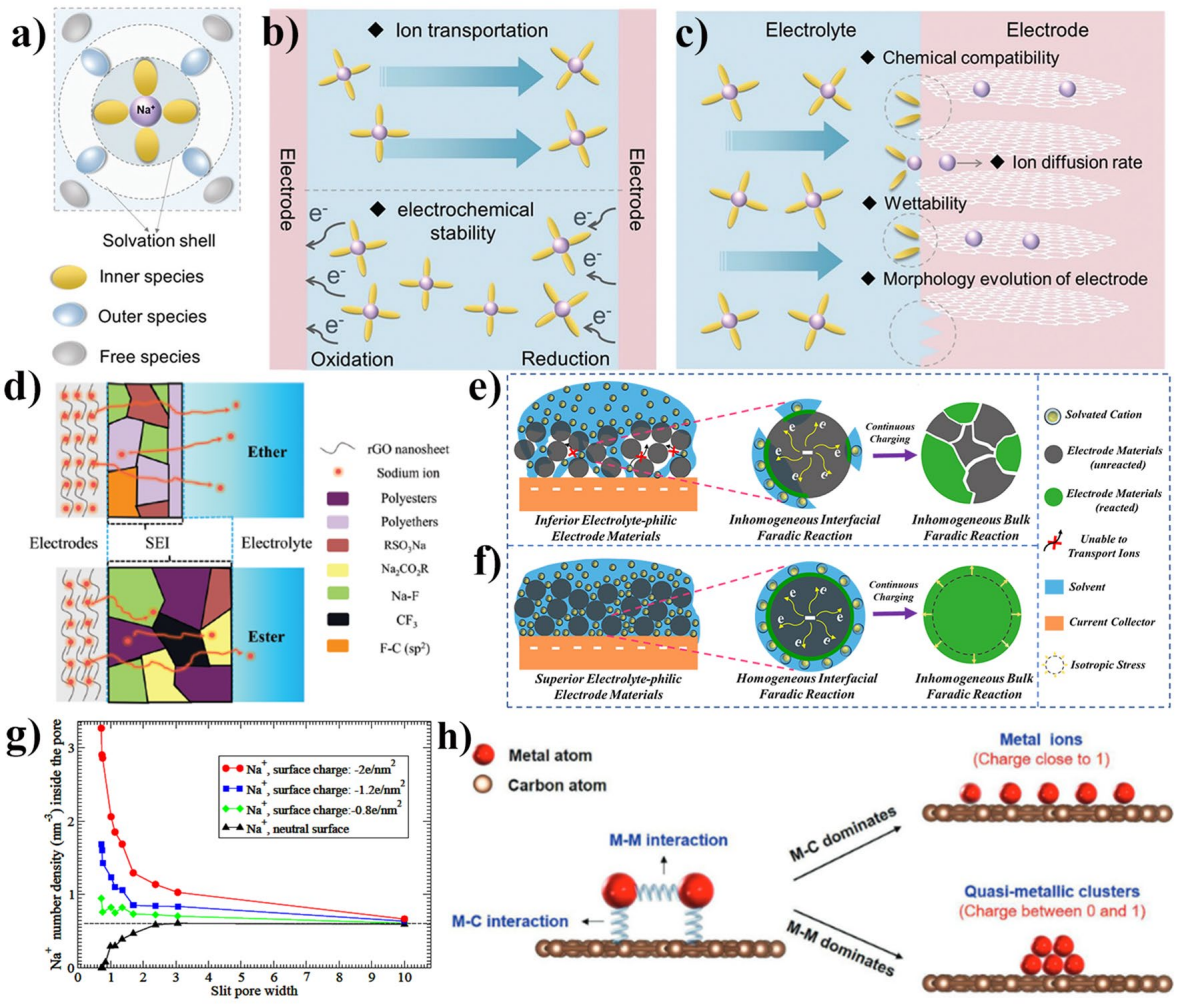
**a** The solvation structure; **b** requirement of high ionic conductivity and fast ion transportation between the electrode and electrolytes; **c** requirements for the electrode surface [160]; **d** different SEI components in different electrolytes [164]; **e** inferior electrolyte-philic electrode materials; **f** superior electrolyte-philic electrode materials [165]; **g** Na^+^ ions number density inside nanopores [166]; **h** alkali-metal storage states on the carbon layer due to the M–M (mental–mental) and M–C (mental–carbon) interactions [167]. **a**–**c** Adapted with permissioned [160], Copyright 2022, the Royal Society of Chemistry. **d** Adapted with permissioned [164], Copyright 2022, the Royal Society of Chemistry. **e**–**f** Adapted with permissioned [165], Copyright 2023, the Royal Society of Chemistry. **g** Adapted with permissioned [166], The Royal Society of Chemistry. **h** Adapted with permissioned [167], Copyright 2020, Elsevier B.V

Because of the violent reaction of alkali metals with water, a sodium-based battery would require an organic-liquid Na^+^ electrolyte [162]. Electrolyte of a sodium-ion battery typically comprise carbonates based and ethers based, corresponding to varying types of sodium salts. The electrolyte ions act as charge carriers exist in form of solvation structure, as is depicted in Fig. 7a. Additionally, the plating of metallic sodium from an organic-liquid electrolyte during charging leads to the formation of anode whiskers (dendrites). These dendrites can grow across a thin separator towards the positive electrode, causing internal short-circuits and the flammable electrolyte igniting. To address the safety concern, significant efforts have been dedicated to in-situ formation of a stable SEI film and construction of artificial SEI layers on electrode surfaces, serving as an effective strategy for mitigating dendrite formation or impeding dendrite penetration [156, 163].

From electrode perspective, the fundamental requirements including chemical compatibility, wettability, morphology evolution of electrode have important influences on the SEI stability, as are illustrated in Fig. 7b, c. The cellulose-based carbon exhibits a network structure with functional groups and defects, which remains intact even after high temperature treatment. The abundant groups and defects on the surface accelerates electrolyte decomposition and easily leads to excessive activity, which makes to form a thicker and more heterogeneous SEI [168]. In our group studies, we conducted a comprehensive analysis of the impact of interface properties on energy storage behavior, with a specific focus on investigating the electrochemical reaction occurring at the interface between electrodes and electrolytes [165, 169, 170]. Although the cellulose precursor is easily modifiable due to its abundant hydroxy groups, the type and contents of oxygen functional groups still a limit for optimizing electrochemical performance of SIBs [171]. Precisely introducing oxygen functional groups into cellulose or cellulose-derived carbon structure to form more stable SEI layer remains a challenge.

It is reported that oxygen function groups introduced into carbon crystalline structure would boost sodium storage kinetics behaviors that relates to SEI composition dominated inorganic or organic components. As shown in Fig. 7d, the constituents of SEI typically consist of organic and inorganic compounds, which is generated through the decomposition of polar solvents and sodium salts [172, 173], respectively. Obviously, the formation of an unstable and thick SEI leads to the successive loss of limited charge carriers, resulting in a rapid drop-off in capacity and limiting the achievable rate capacity. Figure 7e, f illustrates the inferior electrolyte-philic and superior electrolyte-philic electrode materials from interfacial faradaic reaction to bulk faradaic reaction in metal ion batteries. Optimal compatibility between the electrolyte and electrode is essential in preventing undesired side reactions and rapid capacity decay, while superior electrolyte wettability can mitigate battery reaction polarization.

Cellulose-based carbon are composed of small, oriented graphene fragments, which randomly form a network of interconnected micropores and mesopores through the numerous voids created by their haphazard arrangement [174]. Considering that storage sodium mechanism of Na^+^ filling in the pore of carbon anode, Na^+^ concentration after entering into nanopore effect carbon surface charge density due to pore size, as shown in Fig. 7g. Na^+^ can easily enter inner pore with the pore surface density increasing [166]. Besides, the natural and charged pore produces various influence due to surface charge density except uniform pore and slit pore. However, the deposition sodium mental in the pore is considered an unfavorable situation in the charging/discharging process. In Heather’s work, ex-situ ^23^Na solid-state NMR was chosen to investigate the formed metallic sodium clusters. It is found that Na^+^ ions are absorbed at carbon edge and pore surface, forming quasi-metallic sodium in nanopore with increasing of discharging depth [115]. The size of Na metallic clusters increase with nanopore diameter increase, resulting a declined capacity of SIBs and safety problem caused by sodium dendrite in the long-time cycling [175].

Designing a cellulose-derived carbon anode materials with an appropriate pore structure can effectively prevent the deposition of metal sodium inside. Sodium metal exhibits distinctive lattice energy and de-solvation energy and would dissolves in some concentration electrolyte. Specially, the atomic interactions, in Fig. 7h, that is, the interplay of metal–metal (M–M) and metal–carbon (M–C, between the carbon layers and Na ions decide their varying charge storage states in hard carbon anodes [176]. When the dominant interaction is M–C, alkali-metal atoms tend to exist as metal ions with charges close to 1. Conversely, alkali-metal atoms tend to form quasi-metallic clusters with charges between 0 and 1 when the dominant interaction is M–M. The quasi-metallic clusters are notably different from bulk metal formed by metal plating, varying significantly in charge states, sizes, and morphology. The various parameters have a profound impact on the plateau capacity of hard carbon anodes in the low potential region, which indirectly limits the improvement of rate capacity in SIBs.

## Strategies for Improving Rate Performance from Perspective of Cellulose-based Materials

**Fig. 8:**
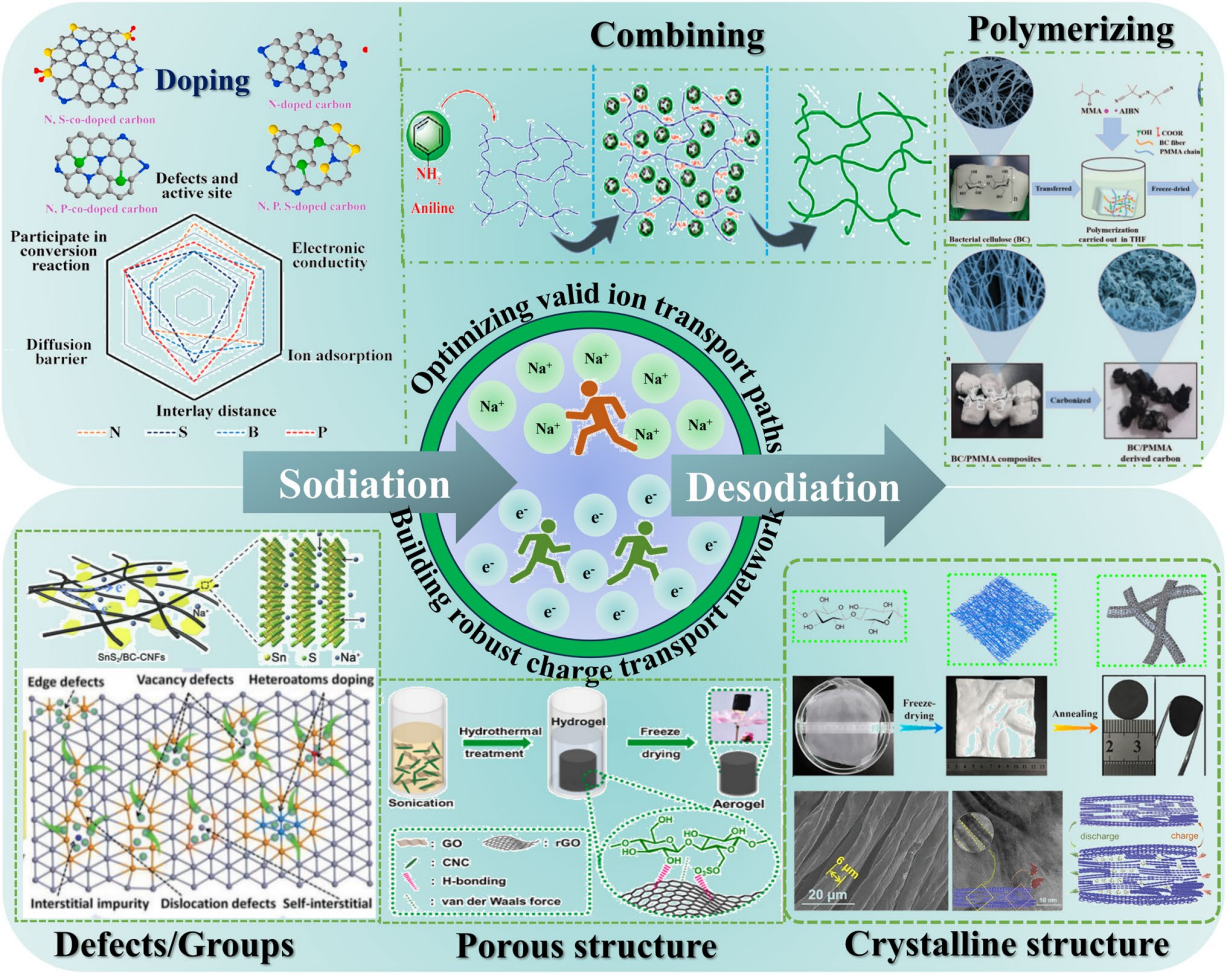
The strategies of improving rate capacity of cellulose-derived carbon materials at cellulose materials level. Optimizing valid ion transport paths strategies on the above line are doping in cellulose precursors [178, 179], combining in cellulose precursors [180], and polymerizing in cellulose precursors [76]; building robust charge transport network strategies on the below line are introducing defects and groups [181], improving the crystalline structure [73, 89], and regulating porous structure [182]. Doping: Adapted with permission [178, 179], Copyright 2020, Elsevier B.V. and Copyright 2021, Royal Society of Chemistry. Combing: Adapted with permission [180]. Copyright 2012, American Chemical Society. Polymering: Adapted with permission [76], Copyright 2022, the Royal Society of Chemistry. Defects/groups: Adapted with permission [181], Copyright 2020, WILEY‐VCH Verlag GmbH & Co. KGaA. Porous structure: Adapted with permission [182], Copyright 2022, Elsevier Limited. Crystalline structure: Adapted with permissioned [73, 89], Copyright 2022, Wiley‐VCH GmbH and Copyright 2019, Elsevier B.V

According to the limited factors that influence on rate performance mentioned in Sect. 4, the strategies for improving rate performance of cellulose-based carbon materials are summarized at the cellulose materials level. Our group discussed the inherent limitations in cellulose-based materials, which encompass impurities within components, a singular chemical structure, uncontrolled crystalline texture, and irregular porous architecture, and further provided an opinion that a pretreatment strategy is necessary to fabricate cellulose-derived materials [177]. Here, two strategies including building robust charge transport network and optimizing valid ion transport pathways, have been developed to obtain the higher rate capacity of cellulose-derived carbon materials at cellulose materials level. Figure 8 is a schematic diagram of optimizing strategies on enhancing the SIB’s rate performance.

### Building Robust Charge Transport Network

A good cellulose-derived carbon electrode requires a well-established charge transport network to provide excellent electronic conductivity. The cellulose’s natural structural feature is an abundant intertwining fiber network that can provide a rich electronic transport channel for cellulose-based anodes materials. Besides, the exceptional crystallinity and mechanical strength of cellulose can establish a stable electrode framework favoring to enhance electronic transfer. For instance, the rate capacity and reversible capacity of cellulose-based carbon are superior to that of lignin-based carbon [13], because single molecule-oriented microcrystalline of cellulose promotes the radial growth of graphite microcrystalline, which facilitates the rearrangement of aromatic rings. The rate performance is determined by the number of electronic carriers, thus doping heteroatoms, combining with conductive materials, and polymerizing in cellulose precursors can alter electron cloud distribution on surface of carbon materials.

#### Pre-doping in Cellulose Precursors

Some methods about heteroatom doping into carbon materials or precursors are various, whose methods include in-situ doping in cellulose-based precursors [66, 183] and doping in cellulose-derived carbons at carbonization process [63], i.e., pre-doping and doping, respectively. There are a large amount of atoms that have been reported to be used as dopant, such as N (nitrogen), S (sulfur), P (phosphorous), B (boron), and F (fluorine, and so on, which could change microstructure and enhance physicochemical properties of obtained carbon materials [63, 66, 75, 80]. Although many researchers have reported on the relationship between doped structure and electrochemical performance, there is currently no evidence detailing how does heteroatom doping influence structure and performance when work as electrode materials.

**Fig. 9:**
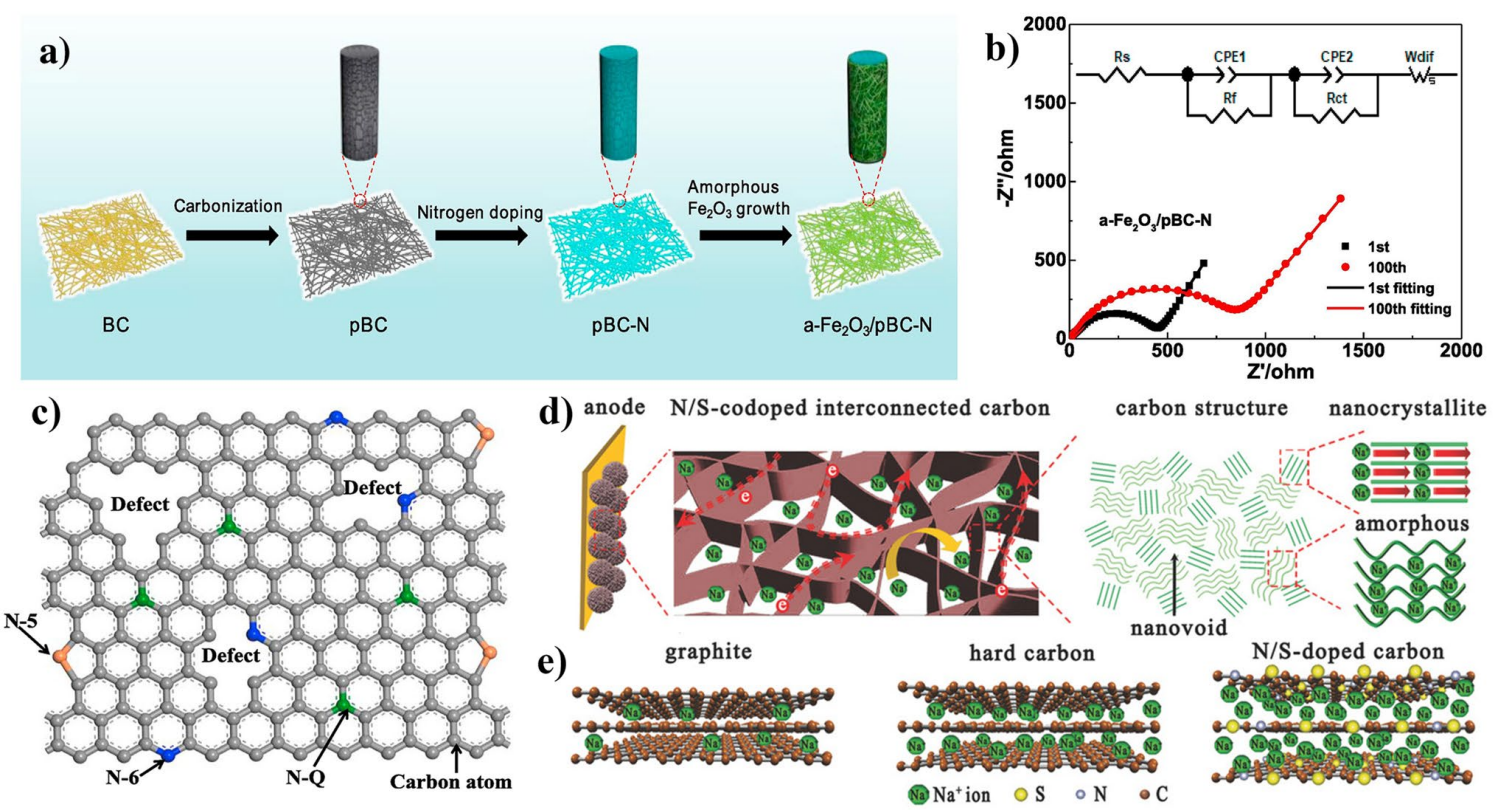
N-doping [185]: **a** fabrication of N-doping composite electrode; **b** EIS spectra of N-doping electrodes at charging state after cycles and the inset images of equivalent circuits; and **c** illustration of N-doping carbon with different doping sites. N/S co-doping [66]: **d** the sodium storage behavior occurring in the N/S-doped carbon; and **e** graphical illustration of the carbon structures in common graphite, undoped hard carbon, and N/S co-doped hard carbon with different interlayer distances and their influences on the Na-storage capabilities. N-doping: **a**–**c** Adapted with permission [185], Copyright 2018, Elsevier B.V. N/S co-doping: **d**, **e** adapted with permission [66],Copyright 2016, WILEY‐VCH Verlag GmbH & Co. KGaA, Weinheim

Among these heteroatoms, N atom is more likely to form chemical bonds with atoms in the adjacent vicinity. Generally, N atom is always from NH_3_, polyacrylonitrile (PAN), polypyrrole (PPy), polyaniline (PANI, and so on. N bond are divided into pyrrolic N, pyridinic N, and quaternary N according to bonding environments [184]. Surface conjugate structure and electron distribution density of carbon material is changed after doping N, leading to increased electrical conductivity and wettability. In Fig. 9a, the N-doped carbon nanofibers obtained by pyrolyzed bacterial cellulose in-situ growth amorphous Fe_2_O_3_, which shows good rate performance, and a rate capability of 408 and 183 mAh g^−1^ at 0.1 A g^−1^ and 3 A g^−1^ [185]. High N content of 10% offers more active site for Na^+^, facilitating electronic transport, as can be seen in Fig. 9b, c. The rate performance exhibits smaller capacity fluctuation, suggesting N-doping forming pyridinic nitrogen adsorption ion by its outermost shell free electrons boost storage sodium, and pyridinic nitrogen would be created defects to increase conductivity of carbon materials.

Apart from N-doping, N/S-doping presents an alternative approach to enhance conductivity and electrochemical performance. Via a “green” and low-cost route Xu et al. prepared hierarchical N/S co-doped carbon microspheres (NSC-SP) by pyrolyzing the cellulose/PANI composite microspheres [66]. As shown in Fig. 9d, the microsphere morphology increase adhesion between collector current and active materials. Meanwhile, the nanosized interconnected nano-walls could provide not only connected pathways for electrons but reduced distance for Na^+^ ion diffusion. It is not ignored that the charge accumulation at the composite interface between the two phases of composite may hinder the efficient charge transfer. Figure 9e describes the carbon structures of common graphite, undoped hard carbon, and N/S co-doped hard carbon with different interlayer distances and their influences on the Na-storage capabilities. The conclusion can be drawn that N/S co-doping expands the distance between carbon interlayers and elevates the Fermi level, resulting in electron-rich carbon materials. This enhancement of electronic conductivity facilitates rapid electron transport, thereby contributing to superior sodium storage performance and excellent rate capability [66].

**Fig. 10:**
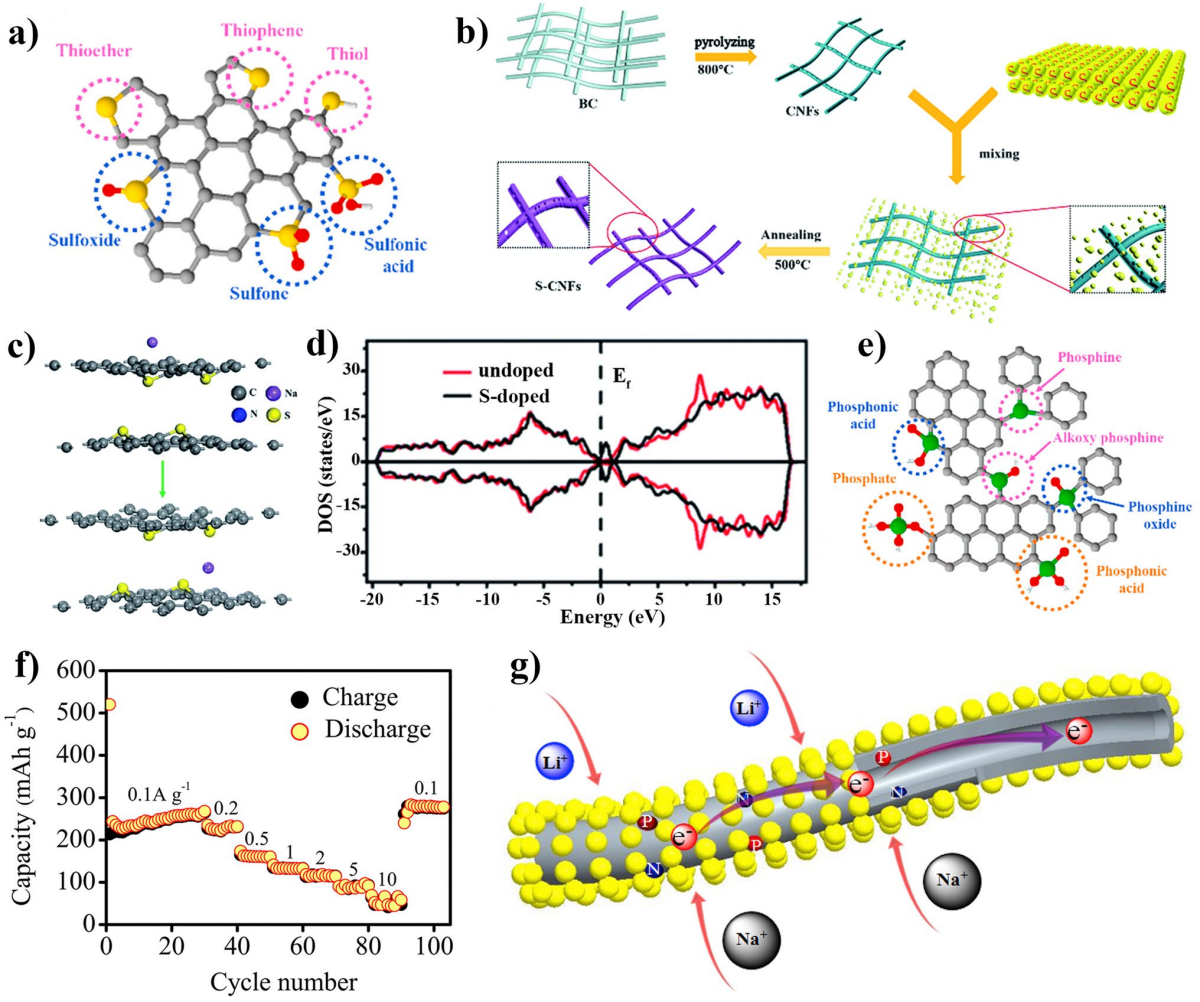
**a** Types of sulfur groups in carbon matrix [178]. S-doping [63]: **b** preparation of S-doped carbon nanofibers; **c** the diffusion path of Na^+^; and **d** the undoped and S-doped carbon density of states. **e** Types of phosphonic groups in the carbon matrix [178]. Double atoms doping [186]: **f** rate performance of *hu*CP/g-C_3_N_4_ as anode for SIBs; and **g** electron transfer and Na^+^ diffusion of *hu*CP/g-C_3_N_4_ electrode. **a** Adapted with permission [178], Copyright 2020, Elsevier B.V. **b**–**e** Adapted with permission [63] Copyright 2018, The Royal Society of Chemistry. **f**, **g** Adapted with permission [186] Copyright 2017, Elsevier Limited

S-doping is a proper choose to develop high-rate capacity carbon anode materials and promote the electronegativity as well as electrochemical activity [80]. Introducing S atoms into *sp*^2^ carbon framework leads to the formation of various sulfur bonding species, including thiol, thiophene (aromatic sulfur), thioether/sulfides (aliphatic sulfur), sulfoxide, sulfone and sulfonic acid [178], as illustrated in Fig. 10a. According to Jin’s research, bacterial cellulose was pyrolyzed at 800 °C and then mixed with sulfur powder at 500 °C in an Ar_2_ atmosphere for S-doping. The synthesis process is illustrated in Fig. 10b [63]. The content of S is up to 15 wt%, enough covalent bonds –C–S–C–) to be linked with carbon nanofibers. Sulfur-dope carbon nanofibers (shorten as S-CNF obey capacitive-like storage sodium and reveal high-rate capability of 257 mAh g^−1^ at 8 A g^−1^, attributed to the surface adsorption of Na^+^ in the nanovoids, defects, and S-doping covalent bonds. Figure 10c is the diffusion routes for Na^+^ transfer, and the enlarger interlayer distance has a lower diffusion barrier and better Na^+^ mobility. From the density of states (DOS) maps in Fig. 10d, the bandgap of the S-doped carbon material is reduced compared to that of the undoped configuration due to defect formation, thereby enhancing the electrical conductivity and demonstrating exceptional rate capability [63].

Compare to N and S, the atom radius of P is larger and donates electron to form chemical bonding in carbons [187]. P doping could improve the charge transfer between sodium and carbon atom bonded with P, strengthening the adsorption of sodium ions. Long bond length of P–C results in a pyramidal-like bonding configuration, as the P atom is pushed away from the carbon plane. This distortion is due to the *sp*^3^ configuration often exhibited by P atoms and leads to an open edge morphology [178, 188]. The introduction of phosphorus doping into carbon initially results in the presence of unstable reduced states, which are gradually oxidized by oxygen groups and humidity to form various oxidized P-containing functional groups [189], as illustrated in Fig. 10e. Tao et al*.* used H_3_PO_4_, hydrazine hydrate, and urea to synthesis N, P dual-doped hollow carbon fibers/graphitic carbon nitride (*hu*CP/*g*-C_3_N_4_) using the common filter paper as a precursor [186]. In Fig. 10f, *hu*CP/*g*-C_3_N_4_ exhibits outstanding rate performance of 93 mAh g^−1^ at 5 A g^−1^, and the reversible capacity still keep 280 mAh g^−1^ when the current density is back to 0.1 A g^−1^. As can be shown in Fig. 10g, the formed conductive network of *g*-C_3_N_4_ nanoparticles and N, P dual-doped hollow carbon fiber facilitate the electronic transport and ionic diffusion.

The Boron atom exhibits a higher affinity for accepting electrons from Na atoms compared to P atom, resulting in enhanced ionic interaction and binding energy. This is attributed to the incorporation of B into C lattice, leading to substitution of certain C sites by B which acts as an electron acceptor [190]. When B is doped on the carbon layer edge, binding energy of heteroatom and Na^+^ would be increased due to B bond with oxygen [191]. B-doped carbon anode promotes the diffusion dynamics of Na^+^ between microcrystalline interlayers, hence facilitating a diffusion-controlled Na^+^ insertion process [192]. Fluorine is an electronegativity atom and endow it ability to reduce repulsion for insertion and extraction of Na^+^, that is, energy barrier of Na^+^ insertion is impaired, and more storage site for Na^+^ binding [193]. Similar to B-doping, single atom doped of F-based cellulose precursors carbon has no literature published.

#### Combining High-Conductive Materials into Cellulose Precursors

Cellulose-based materials can maintain their natural structure after pyrolysis and provide anchoring sites to immobilize active nanoparticles, but their electron conductivity is poor. To address the issue of weakened charge transfer pathways in cellulose-derived carbon materials, a common strategy is to combing with a high conductive material. The internal electronic structure and composition of carbon electrode materials can determine the reaction rate and transfer process, and the electrochemical properties can be changed by adjusting the structure of electrode materials [181].

**Fig. 11:**
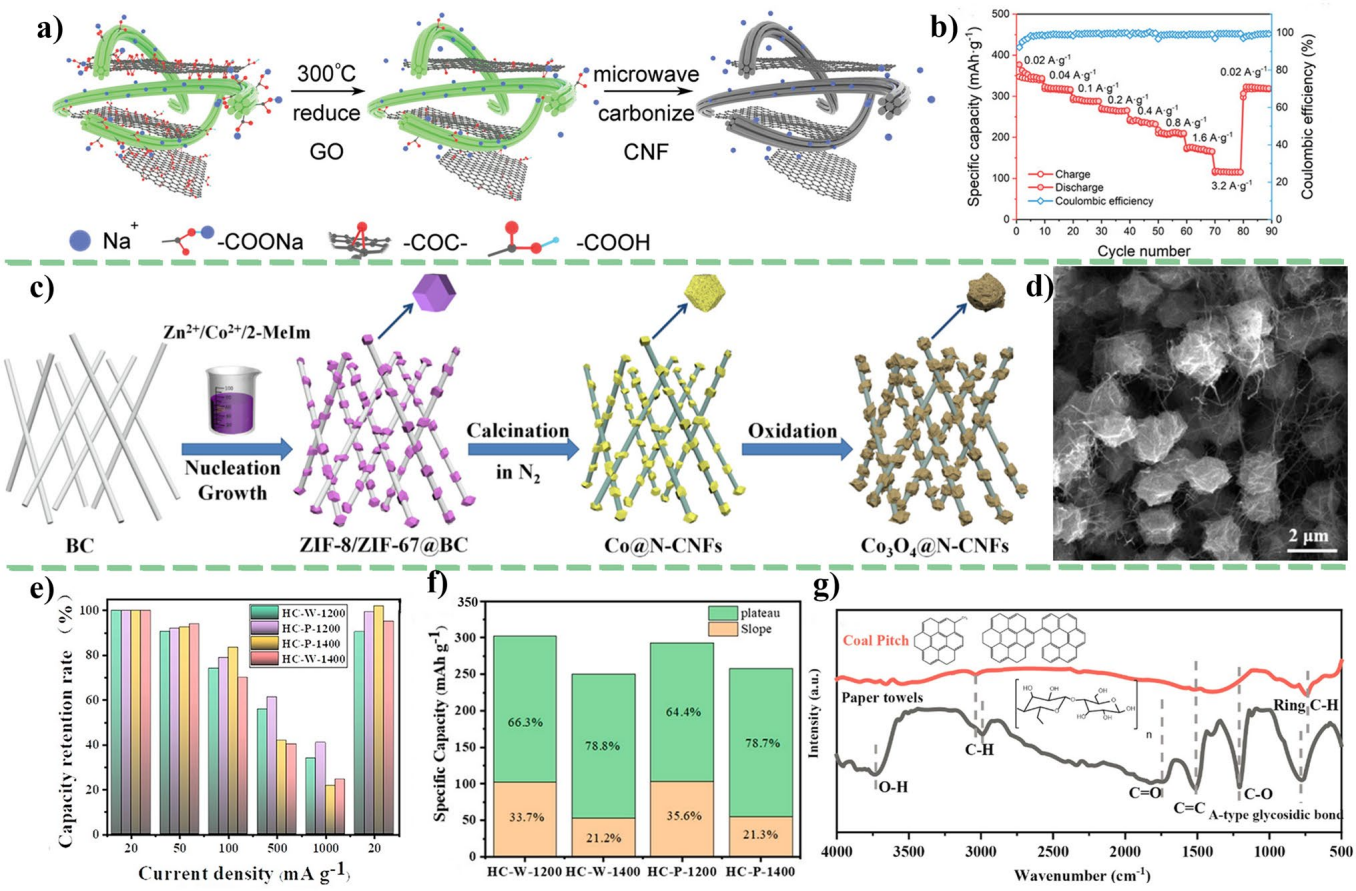
**a** Schematic reduction and microwaving of GO-CNF; **b** rate performance of MrGO-CNF at various current density [135]; **c** schematic illustration of the prepared porous Co_3_O_4_@N-CNFs composite; **d** SEM image of the Co_3_O_4_@N-CNFs [184]; **e** rate performance and capacity retention rates of various carbons; **f** the contribution ratios of slope capacity and plateau capacity; and **g** FTIR spectra of commercial paper towels and coal pitch [195]. **a**, **b** Adapted with permission [135], Copyright 2019, WILEY‐VCH Verlag GmbH & Co. KGaA. **c**–**d** Adapted with permission [184], Copyright 2020, Elsevier B.V. **e**–**g** Adapted with permission [195], Copyright 2021, American Chemical Society

Liu and co-workers prepared bacterial cellulose @polypyrrole composites materials (C-BC@PPy) with a porous carbon network structure and short-range ordered carbon [194]. The anode demonstrates a high capacity of 248 mAh g^−1^ after 100 cycles at 0.05 A g^−1^, and maintains a capacity retention of 176 mAh g^−1^ even after 2000 cycles at 0.5 A g^−1^. Tang et al. [180] synthesized nanocomposite with BC nanofiber-supported PANI using in-situ polymerization, and its electronic conductivity is up to 5.1 S cm^−1^. In Shi and co-workers’ work, they used graphene as an initiator accelerated carbonized cellulose nanofiber (CNFs) with the help of a microwave. The sodium in the carbonaceous materials, introduced from the carbonization of CNFs containing sodium-ion carboxyl, offers favorable spaces for sodiation/de-sodiation, and still maintains in cellulose-based carbon matrix suffering heat treatment to provide an active site for Na^+^ transportation and intercalation [135]. The microwaving graphene oxide/cellulose nanofiber (MrGO-CN**F** chemical variations are shown in Fig. 11a, where the reduction of graphene oxide (GO) occurs upon the removal of oxygen-containing functional groups during the 300 °C treatment, while simultaneous removal of partial functional groups takes place in CNFs. The first discharging capacity is 558 mAh g^−1^, including the capacities of 226 mAh g^−1^ from the sloping region and 332 mAh g^−1^ from plateau region. Besides, MrGO-CNF displays great rate performance of 100 mAh g^−1^ at 3.2 A g^−1^ and return to 0.02 A g^−1^ with 320 mAh g^−1^ discharging capacity, as can be seen in Fig. 11b.

Combining the cellulose precursors with mental organic framework (MOFs) establish a continuous charge transfer mechanism represents a promising approach for constructing a robust network facilitating charge transport. As Fig. 11c, Li and colleagues synthesized a unique three-dimensional porous electrode material by utilizing an in-situ growth method to decorate bacterial cellulose nanofiber networks with Co_3_O_4_ nanoparticles, followed by controlled pyrolysis to obtain Co_3_O_4_@N-CNFs [184]. The Co_3_O_4_@N-CNFs exhibited a porous morphology as revealed by scanning electron microscopy (SEM) in Fig. 11d, which was attributed to the nanofiber network structure derived from bacterial cellulose. Within this carbon framework, uniformly interconnected Co_3_O_4_ nanoparticles were embedded. This unique porous architecture not only provided ample space for accommodating the Co_3_O_4_ particles but also facilitated efficient electron transport. Thus, the Co_3_O_4_@N-CNFs exhibit a rate capability with current densities of 3,200 mA g^−1^ and an average discharging capacity of 205 mAh g^−1^.

The incorporation of metal nanoparticles into cellulose-based composite materials has the potential to enhance the performance of carbon materials derived from cellulose. The cellulose-based materials can be effectively utilized in conjunction with various metal and metal oxide counterparts, for example, Co_3_O_4_, Fe_3_O_4_, CuO, and ZnO [184, 185, 196, 197]. Through the coordination of copper ions (Cu^2+^) with one-dimensional cellulose nanofibrils, Hu et al. discovered that the opening of molecular channels within the typically ion-insulating cellulose allows for rapid transport of ions with high ion conductivity (1.5 × 10^–3^ in the direction of molecular chains). This enables Cu^2+^ ions to move along the polymer chains, resulting in elevated conductivities [196]. Furthermore, the localized carbon structure can be readily modulated by introducing M–N_*x*_–C coordination (M, N, and C denoted as metal, nitrogen, and carbon atoms, respectively) to achieve fast bulk Na^+^ transportation [198]. Lu et al. have successfully synthesized a hard carbon material by incorporating zinc single atoms at the atomic level. This incorporation allows for modulation of both the bulk and surface structure of the hard carbon. The resulting Zn-N_4_-C architecture exhibits catalytic activity in rapidly decomposing NaPF_6_ and facilitating fast SEI interfacial Na^+^ storage kinetics. Additionally, it induces a local electric field within the graphitic domains, which provides an advantageous Coulombic force to accelerate bulk Na^+^ storage kinetics by reducing diffusion barriers [108]. Furthermore, this work suggests potential strategies for further enhancing the rate performance of hard carbon materials and extending these improvements to cellulose-derived carbons.

Soft carbon has a higher degree of order, better conductivity, smaller layer spacing, and excellent rate capacity [199]. The structure combination hard and soft carbon enables to form a novel carbonaceous material, thereby optimizing the electrochemical performance as anode materials [200]. Qiu et al. prepared soft carbon coated on hard carbon surface through with tissue soaking in pitch and then high temperature carbonization [195]. Figure 11e exhibited capacity maintain ratio of carbon paper at different current density. The carbon paper (HC-P-1200) shows good rate capacity due to the long-range-ordered soft carbon terminates the graphene edges of hard carbon. It leads to that reduces SEI formation and the subsequent consumption of sodium-related active substances. Besides, the change trend of plateau and slope capacity display that soft hard coating is a strategy for improve plateau capacity as shown in Fig. 11f. As FTIR of Fig. 11g shows that the soft carbon obtained by direct heat treatment at the same temperature has fewer oxygen-containing functional groups than cellulose-based hard carbon. The slope capacity is reduced by less oxygen-containing defects inhibiting the irreversible capture of sodium ions. Hence, integrating hard carbon with soft carbon represents an effective strategy for obtaining carbon materials with high capacity and excellent rate performance.

#### Introducing Macromolecules into Cellulose Precursors Via In-Situ Polymerization

In 2021, a conductive cellulose nanofiber with graphitized carbon shell extracted directly at lower temperature and pressure via confined chemical transitions method is firstly proposed [201]. The conductive carbon nanofibers exhibit outstanding electron conductivity of 1.099 S cm^−1^. This safe and simple method provide a guideline for cellulose-based high conductivity carbon preparation, but its production process enhances technical costs and yield lower. In this regard, a few researchers studied polymerizations in cellulose precursors for introducing macromolecular components to not only increase carbon yield, but well control cellulose-derived carbon structure via in-situ polymerization, emulsion polymerization, and heat treatment*.*

**Fig. 12:**
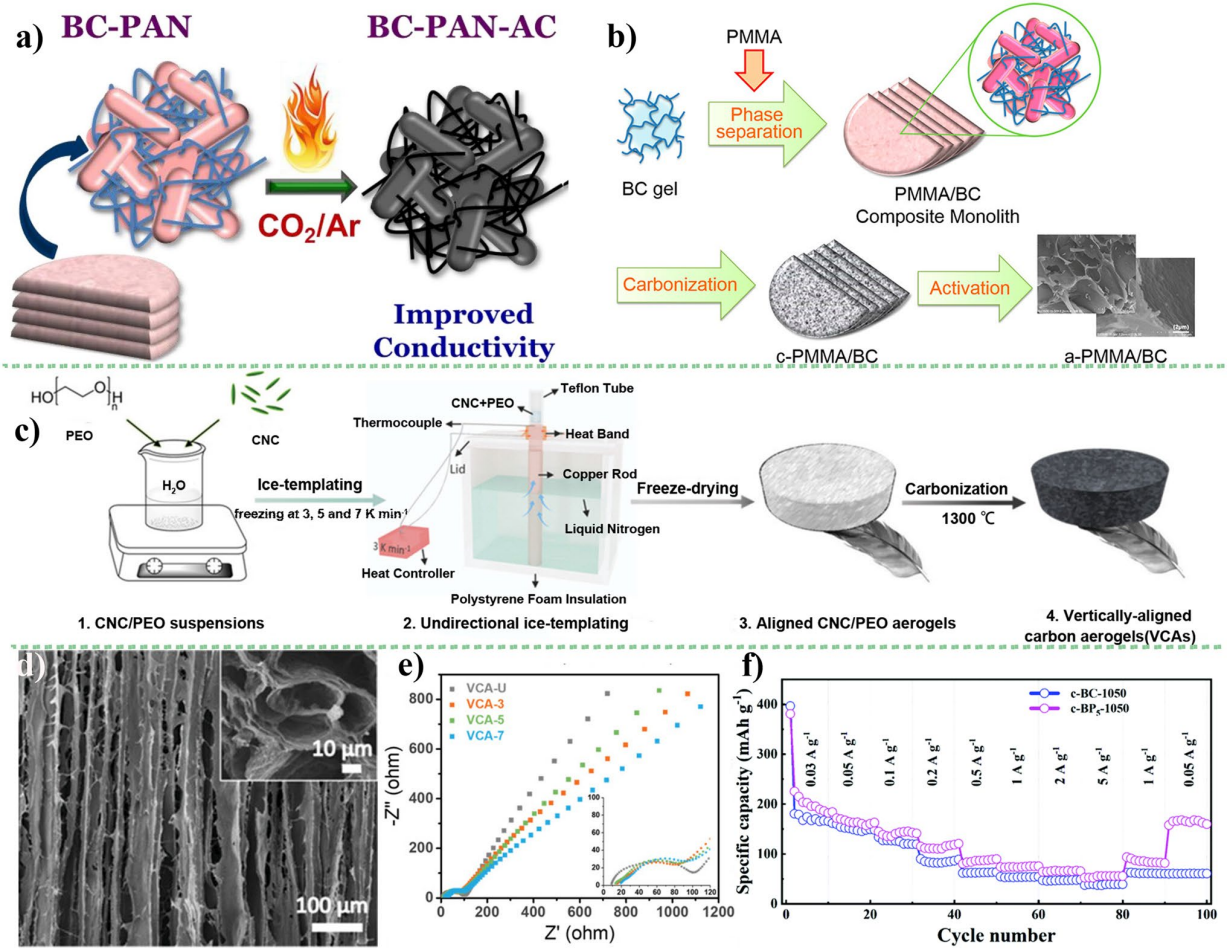
**a** Preparation of activated carbon monolith with microscale layer shape and nanoscale 3D-3D cross-linked structure, BC-PAN-AC [203]; **b** schematic illustration of fabrication route of PMMA/BC composite monolith and its conversion to functional activated carbon [204]; **c** schematic of the synthesis process of CNC/PEO-derived VCAs anodes; and **d** typical SEM images of a cross-sectional and top-view (inset) of the CNC/PEO aerogels. **e** EIS analysis of various cool rate [132]; and **f** rate performance of BC/PMMA derived carbon anode for SIBs [76]. **a** Adapted with permission [203] Copyright 2018, Elsevier Limited. **b** Adapted with permission [204] Copyright 2018, Elsevier Limited. **c**–**e** Adapted with permission [132] Copyright 2022 Advanced Functional Materials published by Wiley‐VCH GmbH. **f** Adapted with permission [76] Copyright 2022, the Royal Society of Chemistry

Several polymer macromolecules, such phenolic resin, epoxy resin, glucose, and dopamine are also employed as hard carbon precursors due to their convenient morphology design and absence of impurities. Because of strong hydrogen bonding, cellulose-based materials are easy to aggregate. Therefore, introducing macromolecules into cellulose precursors via in-situ polymerization can prevent the aggregating and achieve preferred structure of hard carbon, thereby enhancing electrochemical performance [202]. Cellulose was selected by Dobashi et al. to regulate the discontinuity of the polymer carbon network after grinding the powder during electrode preparation, BC-PAN composite materials were synthesized through the incorporation of BC gel into PAN monomer (Fig. 12a). The composites followed by carbonization at 1000 °C to yield cellulose-derived carbon with a continuous porous network structure [203]. Cellulose-derived carbon structures exhibit a cross-linked BC network structure within a PAN porous framework, featuring microscale layered and nanoscale 3D-3D structures that confer advantages as electrode materials. Furthermore, incorporation of BC nanofibers in BC-PAN composites can regulate particle morphology and shape of derived carbon, thereby reducing grain boundary impedance and improving electrical conductivity.

Due to its easy processing and adjustable porosity, PMMA is also a typical polymer that can be used in the preparation of cellulose-derived carbon. Bai et al. utilized a combination of BC and PMMA to synthesize composites featuring a 3D inter-connected porous network through thermally induced phase separation [204]. The schematic illustration of fabrication route of PMMA/BC composite monolith and its conversion to functional activated carbon as show in Fig. 12b. Our group was devoting to modify cellulose precursors structure for improving electrochemical of SIBs. Taking advantage of the abundant micro/mesopores that PMMA pyrolysis formed, the optimized carbon anodes with a high specific surface area display an enhanced Na^+^ diffusion rate with a high capacity of 380.66 mAh g^−1^ at a current density of 0.03 A g^−1^ [76]. What’ more, the stable network structure of BC-based carbon gives continuing conductivity pathway for electron, and display a better rate capacity as can be seen in Fig. 12f.

The microstructure of cellulose carbon materials with a directional charge transfer channel exhibits enhanced rate performance compared to the randomly oriented charge transfer pathway mentioned above. In Wang’s work, a controllable unidirectional ice templating method (Fig. 12c was used to prepare cellulose nanocrystalline (CNC/polyethylene oxide (PEO) composites, and pyrolysis them to obtain vertically aligned carbon aerogels (VCAs) by controlling cool speed [132]. From the cross-section (Fig. 12d and top-view SEMs) insert image of VCAs could see vertically aligned, channeled and honeycomb microstructure. Benefitted to the unique structure and oxygen groups introduced, VCAs-3 exhibits the lowest charge transfer resistance as shown in Fig. 12e. Mixed cellulose and polymer not only provide stable carbon conductive network, but hierarchically tailored and vertically aligned channels acting as ion reservoirs to boost electrolyte percolation. Based on the above, some polymers, as template or reinforcer, can tailor microstructure of cellulose precursor through changing their derived carbon nanodomains feature. Fabricating porous polymer monoliths, which plays a crucial role in tailoring the carbon porous structure, can be achieved through either polymerization or phase separation methods. However, this process is intricate and controlling the fine structure poses challenges [76, 204].

### Optimizing Valid Ion Transport Paths

Historically, decreasing thermodynamic energy barrier and increasing transport efficiency are the two most strategies for improving ion diffusion of anodes in SIBs. In order to decrease energy barrier, some studies stand on the view of defects and functional groups and link the simulation and theory calculated methods to analysis energy barriers change. Another way of ion diffusion to improve is constructing carbon microcrystalline and regulating porous structure. It cannot be overlooked is carbon microcrystalline particle and tortuosity ratio of electrode. Bigger microcrystalline particle is favorable to reversible capacity, but it leads to Na^+^ diffusion limited due to a more quasi graphite sites will grow a bigger graphite domain. As well as tortuosity ratio, which is an essential factor to influence ion diffusion and electronic transfer [205]. It is certain that lower tortuosity shows better transfer kinetic of ions. This section just discusses cellulose-based materials as carbon precursors to achieve fast ion diffusion and optimize the electrochemical performance of SIBs anode with the two strategies as above-mentioned.

#### Introducing Defects and Groups in Cellulose-Derived Carbon

The strategies of design defect carbon materials and function group are proposed to be efficient routes to achieve high Na^+^ storage performance carbon-based materials. On the one hand, the presence of defects can enhance reaction kinetics due to the generation of a significant quantity of active sites. The introducing of defects on the electrode is advantageous for providing additional active sites and ion storage sites, thereby enhancing the insertion of metal ions and facilitating ion diffusion within the material. On the other hand, carboxylic anhydrides and quinone groups own two C=O bonds as a Na^+^ reaction center, show stable configurations and good electronic conductivity, as well as strong Na^+^ adsorption capability [206]. In addition, oxygen functional groups can also improve the wettability of carbon anode toward electrolyte and thereby enhance the rate capability.

Defects can be divided into two classes: intrinsic defect and non-intrinsic defect. The intrinsic defects in crystals of carbon materials include Schottky and Frenkel defects. Schottky defect occurs when atoms or ions leave vacancies in the lattice due to thermal vibration, while Frenkel defect happens when atoms or ions occupy interstitial sites. The electrochemical performance of carbonaceous materials is predominantly influenced by their local structures, particularly the pervasive intrinsic defects. The presence of intrinsic defects can lead to an enhanced capacitive process, and a distinct linear correlation has been observed between the defects and electrochemical performance within a specific range [207, 208].

Introducing defects can impact the intercalation and deintercalation of metal ions within the material layer by reducing stress and electrostatic repulsion between adjacent layers. This directly overcomes migration and diffusion barriers, promoting ion diffusion and charge transfer during the process of metal ion intercalation [181]. It is well known that over-developed defects would break C–C *sp*^2^ conjugated structure and therefore deteriorate the electric conductivity [209]. C–C *sp*^3^ defects enable efficient ion diffusion pathways. Moreover, defects can enhance the surface energy and facilitate electrochemical phase transitions. The strategy of constructing storage sites, which are defect sites, in electrode materials has gradually garnered researchers’ attention due to their role as storage/adsorption/active sites for anchoring additional foreign ions or intermediate species during the reaction process to improve storage capacity [181].

**Fig. 13:**
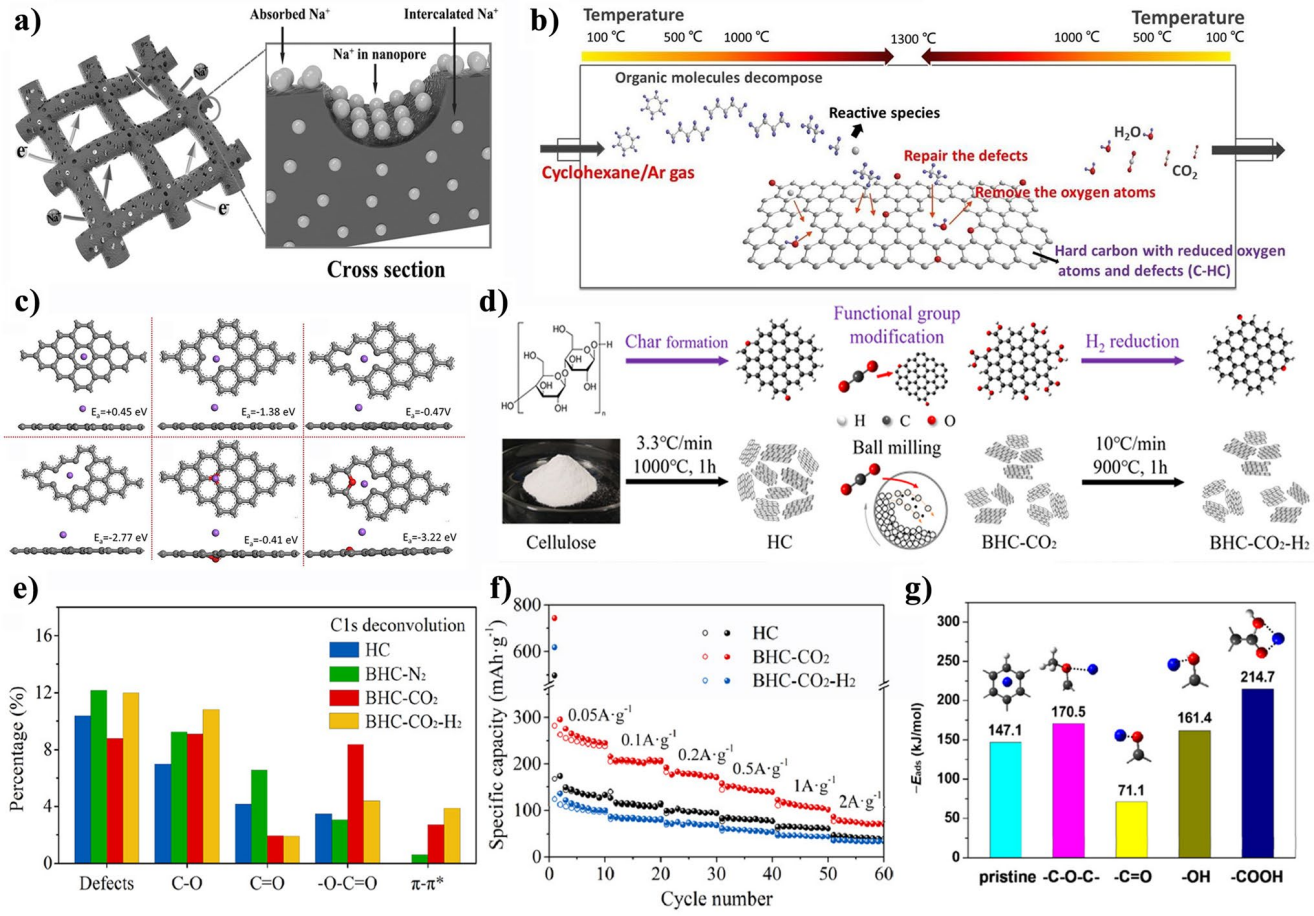
**a** Transportation process of Na^+^ and electron in the carbon network [210]; **b** the innovative in-situ engineering process for residual group control; and **c** atomic structure of Na^+^ absorbed on pure graphene, and different defect graphene [211]. **d** Fabrication process from HC to BHC–CO_2_ and BHC–CO_2_–H_2_; **e** distributions in C1s pattern of O-containing groups for HC, BHC–CO_2_, and BHC–CO_2_–H_2_; **f** rate performances of HC, BHC–CO_2_, and BHC–CO_2_–H_2_ anodes at various current densities; and g adsorption energy of adsorption energy of Na on the pristine carbon surface and near ether, ketone, hydroxyl, as well as carboxyl [70]. **a** adapted with permission [210], Copyright 2016, WILEY‐VCH Verlag GmbH & Co. KGaA; **b**, **c** adapted with permission [211], Copyright 2019, Published by Elsevier Limited. **d**–**g** Adapted with permission [70], Copyright 2019 American Chemical Society

The characterization of defects always used *D* band (disorder or defective structures) and *G* band (graphite structure of Raman spectra to illustration. The intensity ratio of *D* band and *G* band (*I*_D_/*I*_G_ to analysis carbon structure disorder or defective degree. Yu and co-workers synthesized 3D interconnected with N/O-doped carbon (abbreviation NOC network composites, then through carbonized and active with KOH to obtain its derived carbon [210]. Its Raman pattern exhibits higher *I*_D_/*I*_G_ (1.12), and certifies that abundant defects and edge plane exposure exist in NOC. Due to higher specific surface area, the interface of electrode and electrolyte contact well and shortens the diffusion path for Na^+^ (Fig. 13a). The disorder degree decreases with carbonization temperature increasing, therefore, introducing defects to improve ion diffusion speed need to suitably reduce temperature to carbonization. Sun et al*.* designed a new class of hard carbon material with controllable heteroatom doping and defects by an *in-situ* engineering approach, in which certain organic molecular vapor was mixed with Ar_2_ carrier gas during the annealing process [211]. Figure 13b illustrates the *in-situ* engineering process of hard carbon. In Fig. 13c, Na atom demonstrates a binding energy of + 0.45 eV on the pristine graphene sheet, while the adsorption energy for mono-defect, bi-defect, tri-defect, O doped and O&mono-defect doped graphene are − 1.38, − 0.47, − 2.77, − 0.41, and − 3.32 eV, respectively, much lower than that of pristine graphene. Introducing defects into hard carbon would reduce adsorption energy and favorable to ion transfer and diffusion.

Recently, introduction of surface functional groups like oxygen-groups, on the carbon is a promising strategy to improve specific capacity by offering more ion adsorption sites to supply ion valid transport [212]. Exception these advantages, a number of oxygen group on cellulose surface offer active sites for ions, and these ions with generated electronic form a well closed electronic circuit. The various functional groups in cellulose precursors directly influence the cross-linking process during heat treatment, which determine the defect microstructures of cellulose-derived carbon anode. Hu’s group proposed that the rich oxygen groups and unsaturated bond are favorable to keep oxygen atoms cross-linking in the carbon precursors’ structure to form C–O–C groups [213]. To improve the structural diversity of cellulose-derived carbon materials for providing ion diffusion pathways, physical and chemical methods [214], polymer grafting [76], and functional group modification [215] has been used.

A simple mechanochemical strategy is used to introduce oxygen groups in the cellulose precursor [70]. In Fig. 13d, the schematic of cellulose-derived carbon (shorted BHC-CO_2_) prepared via dry ice-assisted ball milling method to introduce oxygen atoms, and certified by the distribution of different types oxygen-containing groups in C 1*s* pattern (Fig. 13e. It is obviously that BHC-CO_2_ possesses the highest carboxyl group percentage of 8.38 at% (that is atomic percent), and exhibits good rate capacity of 293.5 and 122.3 mAh g^−1^ at current densities of 0.05 and 1 A g^−1^ (Fig. 13f. It is because that the surface-dominated Na^+^ adsorption by selectively introducing carboxyl groups is enhanced. According to the interactions between Na and carboxyl-functionalized carbon surface in Fig. 13g, the adsorption energy of Na near carboxyl (− 214.7 kJ mol^−1^) is about 70 kJ mol^−1^, higher than that on the pristine carbon surface (− 147.1 kJ mol^−1^), which indicates that the interaction of Na with carboxyl is much stronger than that with other oxygen-containing functional groups. The result ascribes to strong surface polarization and electron redistribution induced by the carboxyl functional group.

#### Improving the Crystalline Structure of Cellulose-Derived Carbon

The cellulose-derived carbon with a parallel hydrogen bond network exhibits exceptional crystallinity structure, which is beneficial to enhance ions diffusion, electrical conductivity and reduce contact resistance between interfaces. The crystalline structure of cellulose-based carbon materials primarily encompasses the graphitization degree, the thickness (*L*_**c**_, length (*L*_a_), interlayer distance (*d*_002_) of graphite crystallite. The research on controlling interlayer distance is more focused than that on other crystalline parameters. According to the layer spacing, the microcrystalline structure of hard carbon can be classified into three types: highly disordered phases (*d*_002_ > 0.4 nm), quasi-graphite phase (*d*_002_ = 0.36–0.40 nm), and graphite-like phase (*d*_002_ < 0.36 nm) [9].

**Fig. 14:**
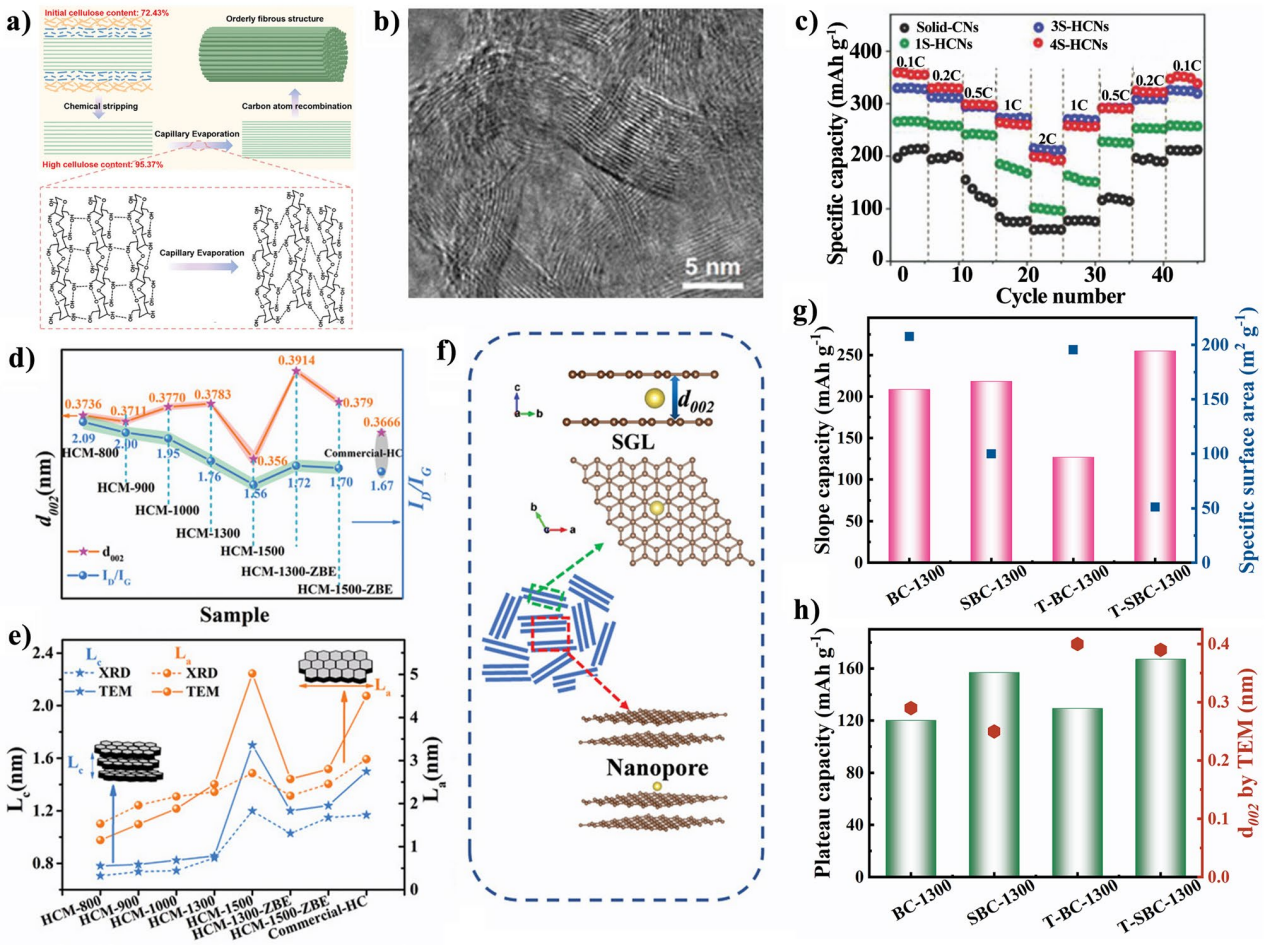
**a** Strategy of preparing high-dense conductive ramie carbon (hd-CRC by pyrolysis after chemical stripping and capillary evaporation on dense cellulose molecules, **b** TEM image of hd-CRC revealing the highly graphitized microcrystals (hGMCs) [217]; **c** The rate performance for four carbon samples with different shell number [218]; **d** Comparison of *d*_002_, *I*_D_/*I*_G_, and **e** *L*_a_ and *L*_c_ of different samples; **f** Model of short graphitic layer and nanopore about DFT calculation [84]; the correction of **g** the specific surface area and slope capacity, **h** the values of *d*_002_ and plateau capacity [82]. **a**, **b** Adapted with permission [217]. Copyright 2023, Wiley‐VCH GmbH. c Adapted with permission [218]. Copyright 2018, WILEY‐VCH Verlag GmbH & Co. KGaA, **d**–**f** Adapted with permission [84], Copyright 2022, Wiley‐VCH GmbH. **g**, **h** Adapted with permission [82] Copyright 2022, Elsevier Limited

High-temperature annealing is proved effective in facilitating atom rearrangement and enhancing graphitization degree, thereby augmenting the conductivity of carbon materials while allowing for tunable carbonaceous framework. Additionally, the graphitic domains of carbon materials may be influenced by the crystallinity of cellulose. The cellulose-derived carbon with lower crystallinity exhibits a highly disordered structure characterized by random chain orientation, which results in the higher rate capacity due to the adsorption of carrier ions on defects, edges, and micropores. Alvin et al. picked sulfur acid with various concentrations to treat microcrystalline cellulose powder, and its derived carbon as anode delivered a high reversible capacity of 322 mAh g^−1^ at 50 mA g^−1^ [216]. Wang and his colleagues prepared highly dense conductive ramie carbon using a capillary evaporation strategy [217], as illustrated in Fig. 14a. The TEM images in Fig. 14b vividly reveal highly graphitized microcrystals (*h*GMCs) and a randomly oriented pseudo-graphite structure, which can provide numerous storage sites for electrolyte ions, benefiting both electron transmission and ion diffusion.

Recently, it is reported that reducing carbon crystalline size could reduce the ion diffusion length, bring high packing density, and improve structural stability [66, 219]. The reason that is the design of nanostructure can address issues in conventional battery materials, such as poor transport properties, significant volume changes at the particle and electrode level, instability of the electrode–electrolyte interface, and structural damage [220]. The precise control of parameters such as size, shape, and morphology associated with Na^+^ transport can lead to varying rate performances, which are important factors among all kinetic processes. In this context, our primary focus lies on optimizing the ion transport properties of nanomaterials in order to enhance ionic and electronic conduction pathways for improving rate performance. Nanofibers could generate interconnected network for Na^+^-ion diffusion channels and its interconnected porosity allows fast access of ionic species to the electrode surfaces [29, 63]. Another shell-core structure carbon also used to study storage sodium performance, because the hollow structure is benefited to boost ion transfer kinetics [218]. With the number of shells increasing, the interface quantity between carbon and electrolyte are increasing and particle inner pores volume are also increasing. Figure 14c shows the rate performance of carbon materials with different number of shells, the clearly specific capacity increasing and charge transfer impendence reducing suggest that the unique structure is more favorable to higher reversible capacity and fast sodium ion transportation kinetics. The density of carbon is an essential factor to influence ions diffusion rate. A thick electrode with lower density carbon leads to a larger volume, but its active mass utilization is limited due to the longer ion diffusion pathway, which hinders volumetric capacitance [221].

Expanding the carbon interlayer distance (*d*_002_) is usually used to improve ion transfer, the suitable distance of Na^+^ insertion/extraction is 0.36 nm. The enlarged lattice distance and large specific surface area lead to excellent Na^+^ storage performance [222]. Exception for *d*_002_, the *L*_a_ and *L*_c_ of graphite crystallite are two important factors to determine ions transportation. Yin et al. synthesized hard carbon with the crystalline feature of larger *L*_a_ and *d*_002_, and smaller *L*_c_ via a ZnO-assisted bulk etching strategy [84]. Besides, the ion diffusion coefficient highest *D*_Na+_ of 10^–6.88^ cm^2^ s^−1^ at the slope region, which slightly drops to 10^–7.48^ cm^2^ s^−1^ at the plateau region. The hard carbon with large *d*_002_, moderate *L*_a_, and *L*_c_, as well as high micropore volume, present higher reversible capacity and plateau capacity, Fig. 14d, e. As shown in Fig. 14f model, density functional theory (DFT) calculations are carried out to further explain that the short graphitic layer structures are beneficial for the adsorption of Na^+^ and have minimal effect on the diffusion of sodium amidst the carbon layers. In addition, the relationship of between cellulose-based carbon structure and storage sodium performance is the focus of our group. In our work, in term of slope and plateau capacity suffer from the crystalline structure change whose change due to H_2_SO_4_ pretreatment cellulose precursors [82]. The two parameters of surface area and *d*_*002*_ did not create positive influence on slope and plateau capacity, and depended on other factors, like pore structure (pore size and pore volum**e**.

#### Regulating Porous Structure of Cellulose-Derived Carbon

Porous carbon materials not only provide efficient ion transport channels and a large number of available storage sites, but also significantly reduce volume expansion and promote disorder degree. Consequently, constructing a porous structure for hard carbon materials is an intensive studying strategy to improve rate performance and plateau capacity. To our knowledge, surface area, pore size distribution, and pore volume are typical parameters to describer properties of porous materials. Plateau region storage sodium capacity is related to nanopores (especially closed pores) [223]. Pore size distribution and surface charge of hard carbon affect whether sodium ions enter into the hard carbon or remain on the hard carbon surface [166]. According to the IPUAC Technical report, macropore refers to a pore diameter of above 50 nm, mesopore refers to a pore diameter between 2 and 50 nm, and micropore refers to a pore diameter of below 2 nm. Moreover, micropore is classified as super-micropore and ultra-micropore (pore diameter < 0.7 nm) [224].

**Fig. 15:**
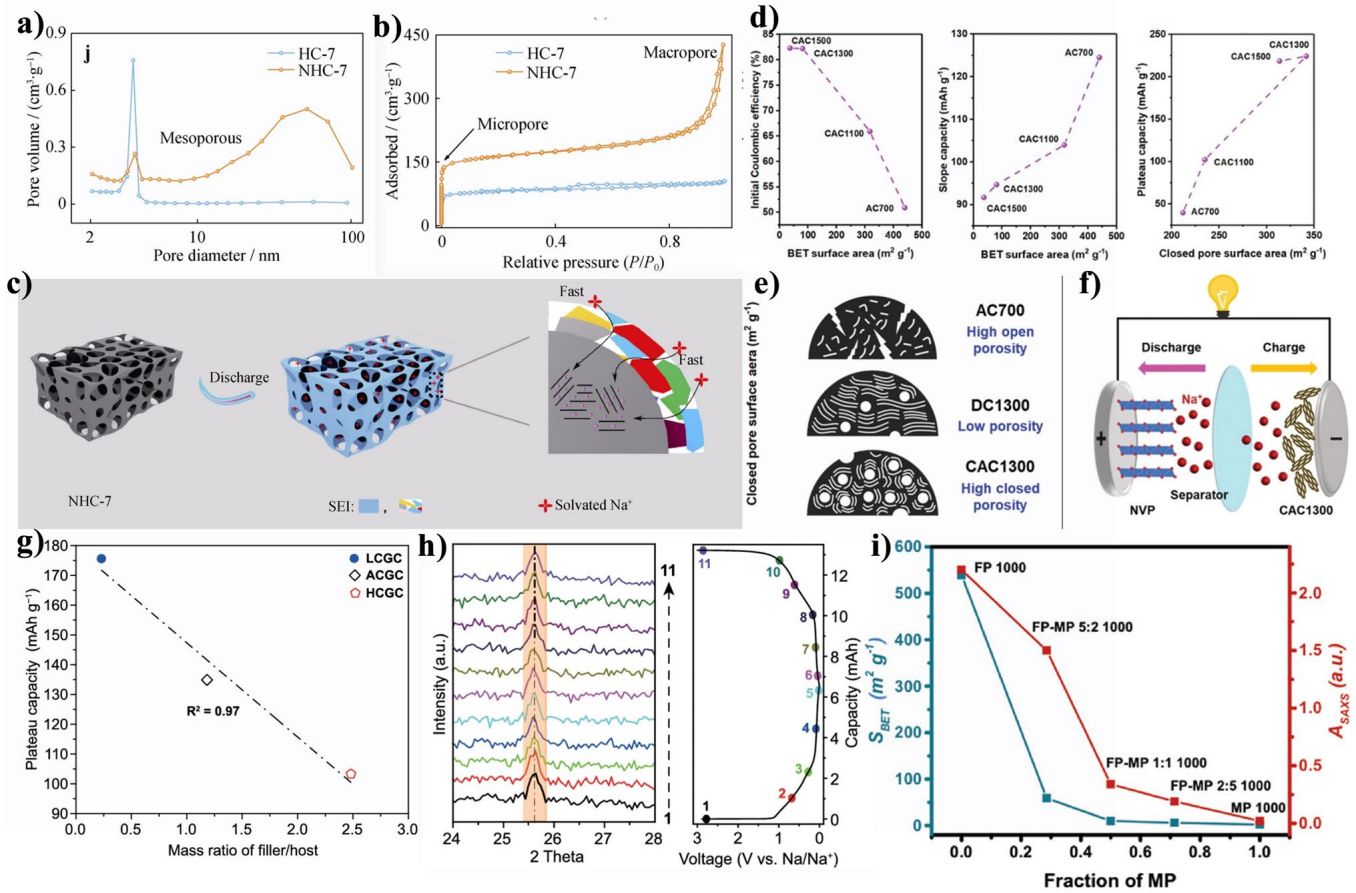
Pore structure: **a** pore size distribution and **b** N_2_ adsorption/desorption isotherms of different hard carbon samples, and **c** schematic diagram of Na^+^ diffusion path in NHC-7 during operation [225]; **d** the relationship of pore structure and ICE, slope capacity, and plateau capacity; **e** schematic illustration of the pore structure evolution during various thermal treatment processes; and **f** sodium-ion battery based on CAC-1300 anode and NVP cathode [226]. **g** The relationship between the plateau capacity and the mass ratio of the filler/host, ACGC, LCGC, and HCGC are corresponding active carbon, lower surface area, and higher surface area after carbonization obtained carbon, and **h** the capacity‑potential curve [227]; and **i** comparison of the A parameters from SAXS in the Porod equation with BET surface area against the amount of MP [134]. **a**–**c** Adapted with permission[225] Copyright 2020, The Nonferrous Metals Society of China and Springer-Verlag GmbH Germany. **d**–**f** Adapted with permission [226] Copyright 2022 Wiley‐VCH GmbH. **g**, **h** Adapted with permission [227] Copyright 2022, Springer Nature. **i** Adapted with permission [134] Copyright 2019, WILEY‐VCH Verlag GmbH & Co. KGaA

Based on the above-mentioned content, porous structure plays an essential role to accelerate rate capacity. The cellulose unique structural feature, ultrafine nanoscale 3D fibrous and entangled network structure, resulting in a higher specific surface area and increased porosity [120]. However, the intrinsic spatial structure at the molecular level imposes limitations on the specific surface area and pore structure of cellulose. For example, Wang et al. synthesized hierarchical porous loose sponge-like hard carbon (NHC-7) with a highly disordered phase prepared at a low temperature of 700 °C [225]. It mainly had a pore diameter of about 4 nm and mesopores at around 50 nm, with a specific surface area of 602.1 m^2^ g^−1^ (Fig. 15a, b. Enhancing electrolyte and electrode contact area and shorting ion diffusion pathways can increase the availability of surface Na^+^-ions, which results from the NHC-7 mesopores structure.

In addition, the hierarchically porous structure enables the construction of continuous connecting channels that accelerate electrolyte transport, resulting in the rapid formation of a complete SEI film (as shown in Fig. 15c) and facilitates its reaction kinetics. The porous structure of nitrogen-rich layered porous carbon materials derived from cellulose can also be effectively changed by boron doping [131]. The porous structure in the interconnecting internal network has thinner pore walls and shorter carbon microcrystal length, and the positive effect of ion diffusion is faster under the weakened diffusion barrier, thus shortening the diffusion time.

Another approach aims to enhance the efficient utilization of materials and optimize the rational distribution of space for optimizing the ion transport process. Qiu’s work prepared hollow hard-soft carbon spheres (HSCSs) by integrated melting-induced impregnation and mesopore-confined carbonization [200]. Meanwhile, combining with surface area and micropore reducing of BET analysis results, HSCSs deliver a relatively higher rate capacity of 210 and 179 mAh g^−1^ at the current densities of 0.1 and 1 A g^−1^. This good conclusion is as follows: the soft carbon can enter into the pores and defects of hard carbon substances to tailor pore structure, which achieves transformation from mesopore to micropore and closed pore with the help of high temperature. In addition, hollow cavity can act as an electrolyte reservoir to decrease the diffusion distance from the bulk electrolyte to the electrode surface. Wang and co-workers used the altered thermal transformation pathway of chemical activation followed by high-temperature carbonization is adopted to achieve the conversion of open nanopores and ordered carbon crystallite into closed pores surrounded by short-range carbon structures and their carbon-marked CAC-1300 [226]. They discussed in details that pore structure (surface area and closed pore created an impact on ICE, slope capacity, and plateau capacity (Fig. 15d. Figure 15e describes the process for successfully obtained change from open pore and closed pore. With Na_3_V_2_(PO_4_)_3_ (NVP) as a cathode and CAC-1300 as an anode to assemble full-cell, it shows good rate performance of 170.7 mAh g^−1^ at 1 °C and energy density up to 231.2 Wh kg^−1^, as is shown in Fig. 15f.

Excepted among micropore, mesopore, and closed pore transformation, ultra-micropore structure is benefitted from ion diffusion and boosting rate capacity. Ultra-micropores (< 0.5 nm) of hard carbon can function as ionic sieves to reduce the diffusion of solvated Na^+^ but allow the entrance of naked Na^+^ into the pores, which can reduce the interface contact between the electrolyte and the inner pores without losing the fast diffusion kinetics [227]. A lower filler/host ratio corresponding to more ultra-micropores can ensure a larger plateau capacity from Fig. 15g of quantitative relationship. And the rated capacity keeps 346 and 125 mAh g^−1^ at 0.3 and 2 A g^−1^. From Fig. 15h, one can know without peak change in XRD pattern during SIBs operation, which suggests no intercalation/deintercalation into/from the graphitic interlayers in both the sloping region and plateau region. Therefore, the existence of an ultra-micropore can provide a more active site for ion storage and expand ion diffusion accessibility. The previously mentioned combination of hard and soft carbon to tailor closed pore numbers by changing the mass ratio of hard and soft carbon [134]. Small-angle X-ray scattering (SAXS) and N_2_ adsorption methods are used to investigate the pore structure and surface area variation (Fig. 15i), which express that the soft carbon limits access to the pore surface resulting in a higher fraction of closed pores. Meanwhile, hard-soft carbon, as an anode for SIBs, shows excellent electrochemical performance, especially rate, due to soft carbon precursors blocking open pores of hard carbon and keeping a stable structure for ion diffusion.

The self-supporting carbon electrodes with advantageous porous structure for valid ion transportation have reported studies [72, 89, 195]. Hu’s group focused on the conversion of wood-based materials into carbon electrodes through carbonization. The resulting carbon exhibits a unique mesoporous structure with a high percentage of short-range ordered lattice and a large amount of carbon nanofibers [72]. By controlling thickness of the cut wood, they were able to achieve various thicknesses for the free-standing carbon electrode [205]. The development of free-standing electrodes has promoted research on thick electrodes. In addition to wood, bacterial cellulose has been studied by direct carbonization to prepare self-supporting materials [89]. Under the carbonization condition of 1300 °C, the resulting carbon films exhibit excellent rate capability, with values of 147 and 128 mAh g^−1^ at 10 and 20 A g^−1^, respectively. The limited applications of the flexible electrode are due to its inability to load sufficient mass of active materials. Guo et al. developed a self-supporting electrode (NVP@C-CC using carbon cloth (CC as a support material for loading high mass Na_3_V_2_(PO_4_)_3_@carbon (NVP@C [228]. The NVP@C-CC exhibits a higher rate capacity of 96.8 mAh g^−1^ at 100 °C and 69.9 mAh g^−1^ at 200 °C, while also providing the cell with high energy and power densities of 396 Wh kg^−1^ and 97 kW kg^−1^.

There are some questions for influence on ions diffusion of pore structure. Whether closed pore or open pore? micropore or mesopore? Which of them is more favorable to sodium ion diffusion? More and more attentions have been paid to the fact that closed pore make real effect on storage sodium ion behaviors. For an example, with temperature increasing, waste cork-derived hard porous carbon with rich closed pore is transformed open pore [117]. Zhao et al. synthesized carbon materials via altering thermal transformation pathway of chemical activation followed by high-temperature carbonization [226]. Carbon with micro/mesoporous could offer more contacts between the sodium ions and the host materials, shortening the ion transport path lengths and accelerating the electrochemical reactions [113, 229]. Compared with open pores, in which de-solvated Na^+^ ions enter through these openings, diffuse and gather inside the pore, the storage sodium behaviors of closed pore structures are tended to near metallic sodium deposition.

## Summary and Perspectives

Raising the fast-charging capability of sodium ions batteries is a key requirement for the adoption of current battery-based electric vehicles and consumer electronics. However, current sodium-ion batteries are still unable to support ultrafast power input due to the larger ionic radium, resulting the capacity fade at high current densities, that is also a key technological barrier for hard carbon anodes. In this review, we systematically summarize the structural characteristics of cellulose materials and cellulose-derived carbon materials. Taking into account the intrinsic structural limitation of cellulose materials, we discuss two strategies for enhancing the rate-capability of cellulose-derived carbon materials from the perspective of ion diffusion and charge transfer. Specific methods include but not limited to, constructing an interconnected transportation network, regulating molecular structure of cellulose precursors, increasing the graphitization degree, and tailoring crystalline structures of cellulose-derived carbons. Despite a series of cellulose-derived hard carbon have been applied in sodium ions batteries, there are some key points that need to be addressed on developing high-rate performance of anode materials.

(i) Increasing the Purity of Cellulose-Based Materials
  It is well-known that a large amount of composition such as cellulose, hemicellulose, lignin, starch, and ash exist in natural cellulose-based materials. When cellulose-based materials are carbonized at high temperature, the structure of cellulose-derived carbon is determined through the interaction of these components. Among these components, a few of impurities have negative effect on the electronic conductivity of cellulose-derived carbons, thus affecting the rate performance of SIBs. Since residual impurities in chemical components can negatively affect the performance and also lead to a serious increase in side reaction, it is highly desirable to develop a suitable purification process to decrease the amounts of impurities in cellulose-based precursors.
(ii) Optimizing the Crystalline Structure at Molecular Level
  A large number of hydroxyl groups exist on the cellulose chain that can form internally strong hydrogen bonds, which make other active groups or molecular hard to access. The most commonly employed chemical modification of cellulose involves substituting the alcoholic hydroxyl group at the C6 position in cellulose chains, which preserves precursors’ inherent morphology and a high carbon yield. At present, the mechanism on thermochemical changes and the microstructure of hard carbon during cellulose materials pyrolysis remains unclear, while further investigation is required to determine how the reactivity of other alcohol hydroxyl groups (such as C_2_-OH affects porous structure. The systematic research will help to accurately predict the properties of carbon materials upon formation. Therefore, molecule-level functionalization or modification is beneficial to optimize the crystalline of cellulose-based materials, enabling regulate graphite and disorder structure of cellulose-derived carbon materials.
(iii) Valuing Pre-treatment Process Before High-Temperature Pyrolysis
  Due to their varying compositions, irregular porous structure, and complex carbonization, cellulose-based precursors have an uncontrolled crystalline structure. Besides, the pyrolysis behavior of cellulose-based materials is influenced by variations in chemical molecular, functional groups, and aggregation states of precursors. Therefore, pretreatment strategies are necessary to optimize the structure of cellulose-derived hard carbon for sodium ion batteries. It includes not only purification component of precursors using chemical/physical methods, but also pre-carbonized process aiming at intermediate carbon products. Through pre-treatment process, cellulose-derived carbon materials show a higher plateau and slope capacity, exhibiting a better rate performance.
(iv) Regulating Carbon Edge Structure and Pore Engineering
  Advanced cellulose-derived anodes with high-rate performance range by employing appropriate carbon edge structure and pore engineering are critical, since the both dictates the ion transport and interfaces for a given battery chemistry. Carbon structures with reduced open pores, abundant closed pores, and appropriate carbon interlayer spacing present a formidable challenge. High-temperature treatment can effectively decrease the specific surface area of cellulose-derived carbons and convert their open pores into closed ones, enhancing plateau capacity and initial coulombic efficiency. Nevertheless, significantly decreased interlayer spacing caused by high-temperature treatment may hinder the transport of sodium ions. Therefore, these critical parameters should be carefully balanced through optimizing precursors or pyrolysis processes to explore the relationship between carbon microstructure and performance in different electrolytes for designing promising carbon materials for SIBs.

Huge advances in designing cellulose-derived anode materials with excellent rate performances have been achieved, however, there are many challenges to further improve rate performances, especially how to better combine each strategy and balance their advantage and shortcomings. We believe that the guidelines presented will provide an avenue for designing high-rate anodes for next-generation sodium-ion batteries.

## References

[1] S. Chu, A. Majumdar, Opportunities and challenges for a sustainable energy future. Nature **488**, 294–303 (2012). 10.1038/nature11475 22895334 10.1038/nature11475

[2] T. He, X. Kang, F. Wang, J. Zhang, T. Zhang et al., Capacitive contribution matters in facilitating high power battery materials toward fast-charging alkali metal ion batteries. Mater. Sci. Eng. R. Rep. **154**, 100737 (2023). 10.1016/j.mser.2023.100737

[3] C. Zhao, Q. Wang, Z. Yao, J. Wang, B. Sánchez-Lengeling et al., Rational design of layered oxide materials for sodium-ion batteries. Science **370**, 708–711 (2020). 10.1126/science.aay9972 33154140 10.1126/science.aay9972

[4] B. Dunn, H. Kamath, J.-M. Tarascon, Electrical energy storage for the grid: a battery of choices. Science **334**, 928–935 (2011). 10.1126/science.1212741 22096188 10.1126/science.1212741

[5] Y. Wan, Y. Liu, D. Chao, W. Li, D. Zhao, Recent advances in hard carbon anodes with high initial Coulombic efficiency for sodium-ion batteries. Nano Mater. Sci. **5**, 189–201 (2023). 10.1016/j.nanoms.2022.02.001

[6] F. Song, J. Hu, G. Li, J. Wang, S. Chen et al., Room-temperature assembled MXene-based aerogels for high mass-loading sodium-ion storage. Nano Micro Lett. **14**, 37 (2021). 10.1007/s40820-021-00781-6 10.1007/s40820-021-00781-6 PMC8683516 34919180

[7] M. Wang, Q. Wang, X. Ding, Y. Wang, Y. Xin et al., The prospect and challenges of sodium-ion batteries for low-temperature conditions. Interdiscip. Mater. **1**, 373–395 (2022). 10.1002/idm2.12040

[8] S. Iijima, Helical microtubules of graphitic carbon. Nature **354**, 56–58 (1991). 10.1038/354056a0

[9] N. Sun, Z. Guan, Y. Liu, Y. Cao, Q. Zhu et al., Extended “adsorption–insertion” model: a new insight into the sodium storage mechanism of hard carbons. Adv. Energy Mater. **9**, 1901351 (2019). 10.1002/aenm.201901351

[10] D. Saurel, B. Orayech, B. Xiao, D. Carriazo, X. Li et al., From charge storage mechanism to performance: a roadmap toward high specific energy sodium-ion batteries through carbon anode optimization. Adv. Energy Mater. **8**, 1703268 (2018). 10.1002/aenm.201703268

[11] Y. Chu, J. Zhang, Y. Zhang, Q. Li, Y. Jia et al., Reconfiguring hard carbons with emerging sodium-ion batteries: a perspective. Adv. Mater. **35**, e2212186 (2023). 10.1002/adma.202212186 36806260 10.1002/adma.202212186

[12] A. Siddiqa, Z. Yhobu, D.H. Nagaraju, M. Padaki, S. Budagumpi et al., Review and perspectives of sustainable lignin, cellulose, and lignocellulosic carbon special structures for energy storage. Energy Fuels **37**, 2498–2519 (2023). 10.1021/acs.energyfuels.2c03557

[13] X.-S. Wu, X.-L. Dong, B.-Y. Wang, J.-L. Xia, W.-C. Li, Revealing the sodium storage behavior of biomass-derived hard carbon by using pure lignin and cellulose as model precursors. Renew. Energy **189**, 630–638 (2022). 10.1016/j.renene.2022.03.023

[14] H. Yang, B. Huan, Y. Chen, Y. Gao, J. Li et al., Biomass-based pyrolytic polygeneration system for bamboo industry waste: evolution of the char structure and the pyrolysis mechanism. Energy Fuels **30**, 6430–6439 (2016). 10.1021/acs.energyfuels.6b00732

[15] D. Zhao, Y. Zhu, W. Cheng, W. Chen, Y. Wu et al., Cellulose-based flexible functional materials for emerging intelligent electronics. Adv. Mater. **33**, e2000619 (2021). 10.1002/adma.202000619 32310313 10.1002/adma.202000619

[16] H. Yamamoto, S. Muratsubaki, K. Kubota, M. Fukunishi, H. Watanabe et al., Synthesizing higher-capacity hard-carbons from cellulose for Na- and K-ion batteries. J. Mater. Chem. A **6**, 16844–16848 (2018). 10.1039/c8ta05203d

[17] W. Tao, J. Chen, C. Xu, S. Liu, S. Fakudze et al., Nanostructured MoS_2_ with interlayer controllably regulated by ionic liquids/cellulose for high-capacity and durable sodium storage properties. Small **19**, e2207397 (2023). 10.1002/smll.202207397 36693782 10.1002/smll.202207397

[18] H. Seddiqi, E. Oliaei, H. Honarkar, J. Jin, L.C. Geonzon et al., Cellulose and its derivatives: towards biomedical applications. Cellulose **28**, 1893–1931 (2021). 10.1007/s10570-020-03674-w

[19] Y. Zhao, Y. Zhang, M.E. Lindström, J. Li, Tunicate cellulose nanocrystals: preparation, neat films and nanocomposite films with glucomannans. Carbohydr. Polym. **117**, 286–296 (2015). 10.1016/j.carbpol.2014.09.020 25498637 10.1016/j.carbpol.2014.09.020

[20] R.M. Brown Jr., J.H. Willison, C.L. Richardson, Cellulose biosynthesis in *Acetobacter xylinum*: visualization of the site of synthesis and direct measurement of the in vivo process. Proc. Natl. Acad. Sci. U.S.A. **73**, 4565–4569 (1976). 10.1073/pnas.73.12.4565 1070005 10.1073/pnas.73.12.4565 PMC431544

[21] L. Ma, Z. Bi, Y. Xue, W. Zhang, Q. Huang et al., Bacterial cellulose: an encouraging eco-friendly nano-candidate for energy storage and energy conversion. J. Mater. Chem. A **8**, 5812–5842 (2020). 10.1039/c9ta12536a

[22] N.I. Tkacheva, S.V. Morozov, I.A. Grigor’ev, D.M. Mognonov, N.A. Kolchanov, Modification of cellulose as a promising direction in the design of new materials. Polym. Sci. Ser. B **55**, 409–429 (2013). 10.1134/s1560090413070063

[23] J. Prachayawarakorn, S. Chaiwatyothin, S. Mueangta, A. Hanchana, Effect of jute and kapok fibers on properties of thermoplastic cassava starch composites. Mater. Des. **47**, 309–315 (2013). 10.1016/j.matdes.2012.12.012

[24] H. Krässig, J. Schurz, R.G. Steadman, K. Schliefer, W. Albrecht et al., Cellulose, in *Ullmann’s Encyclopedia of Industrial Chemistry*. (Wiley, USA, 2004), pp.11–95

[25] B. Puangsin, H. Soeta, T. Saito, A. Isogai, Characterization of cellulose nanofibrils prepared by direct TEMPO-mediated oxidation of hemp bast. Cellulose **24**, 3767–3775 (2017). 10.1007/s10570-017-1390-y

[26] B.J.C. Duchemin, Structure, property and processing relationships of all-cellulose composites. PhD thesis, Université du Havre (2008). https://www.researchgate.net/publication/29488814

[27] S. Rongpipi, D. Ye, E.D. Gomez, E.W. Gomez, Progress and opportunities in the characterization of cellulose—an important regulator of cell wall growth and mechanics. Front. Plant Sci. **9**, 1894 (2019). 10.3389/fpls.2018.01894 30881371 10.3389/fpls.2018.01894 PMC6405478

[28] G. Zheng, Y. Cui, E. Karabulut, L. Wågberg, H. Zhu et al., Nanostructured paper for flexible energy and electronic devices. MRS Bull. **38**, 320–325 (2013). 10.1557/mrs.2013.59

[29] Y. Luo, J. Zhang, X. Li, C. Liao, X. Li, The cellulose nanofibers for optoelectronic conversion and energy storage. J. Nanomater. **2014**, 654512 (2014). 10.1155/2014/654512

[30] T. Li, X. Zhang, S.D. Lacey, R. Mi, X. Zhao et al., Cellulose ionic conductors with high differential thermal voltage for low-grade heat harvesting. Nat. Mater. **18**, 608–613 (2019). 10.1038/s41563-019-0315-6 30911121 10.1038/s41563-019-0315-6

[31] A. Isogai, T. Saito, H. Fukuzumi, TEMPO-oxidized cellulose nanofibers. Nanoscale **3**, 71–85 (2011). 10.1039/c0nr00583e 20957280 10.1039/c0nr00583e

[32] H.-P. Fink, B. Philipp, D. Paul, R. Serimaa, T. Paakkari, The structure of amorphous cellulose as revealed by wide-angle X-ray scattering. Polymer **28**, 1265–1270 (1987). 10.1016/0032-3861(87)90435-6

[33] M. Wada, M. Ike, K. Tokuyasu, Enzymatic hydrolysis of cellulose I is greatly accelerated via its conversion to the cellulose II hydrate form. Polym. Degrad. Stab. **95**, 543–548 (2010). 10.1016/j.polymdegradstab.2009.12.014

[34] Q. Wu, J. Xu, Z. Wu, S. Zhu, Y. Gao et al., The effect of surface modification on chemical and crystalline structure of the cellulose III nanocrystals. Carbohydr. Polym. **235**, 115962 (2020). 10.1016/j.carbpol.2020.115962 32122497 10.1016/j.carbpol.2020.115962

[35] H. Zhang, Q. Li, K.J. Edgar, G. Yang, H. Shao, Structure and properties of flax vs. lyocell fiber-reinforced polylactide stereo complex composites. Cellulose **28**, 9297–9308 (2021). 10.1007/s10570-021-04105-0

[36] J. Sugiyama, R. Vuong, H. Chanzy, Electron diffraction study on the two crystalline phases occurring in native cellulose from an algal cell wall. Macromolecules **24**, 4168–4175 (1991). 10.1021/ma00014a033

[37] P. Langan, Y. Nishiyama, H. Chanzy, X-ray structure of mercerized cellulose II at 1 a resolution. Biomacromol **2**, 410–416 (2001). 10.1021/bm005612q 10.1021/bm005612q 11749200

[38] M. Wada, L. Heux, A. Isogai, Y. Nishiyama, H. Chanzy et al., Improved structural data of cellulose III_I_ prepared in supercritical ammonia. Macromolecules **34**, 1237–1243 (2001). 10.1021/ma001406z

[39] P. Zugenmaier, Conformation and packing of various crystalline cellulose fibers. Prog. Polym. Sci. **26**, 1341–1417 (2001). 10.1016/s0079-6700(01)00019-3

[40] T.L. Bluhm, A. Sarko, Packing analysis of carbohydrates and polysaccharides. V. Crystal structures of two polymorphs of pachyman triacetate. Biopolymers **16**, 2067–2089 (1977). 10.1002/bip.1977.360160917 901927 10.1002/bip.1977.360160917

[41] M. Wada, L. Heux, J. Sugiyama, Polymorphism of cellulose I family: reinvestigation of cellulose IV_I_. Biomacromol **5**, 1385–1391 (2004). 10.1021/bm0345357 10.1021/bm0345357 15244455

[42] Y. Liu, H. Fu, W. Zhang, H. Liu, Effect of crystalline structure on the catalytic hydrolysis of cellulose in subcritical water. ACS Sustain. Chem. Eng. **10**, 5859–5866 (2022). 10.1021/acssuschemeng.1c08703

[43] J.C. Arthur, *Chemical Modification of Cellulose and its Derivatives* (Springer, 1989), pp.49–80

[44] P. Trivedi, P. Fardim, Recent Advances in Cellulose Chemistry and Potential Applications, in *Production of Materials from Sustainable Biomass Resources*. (Springer, Singapore, 2019), pp.99–115

[45] D.K. Shen, S. Gu, A.V. Bridgwater, The thermal performance of the polysaccharides extracted from hardwood: cellulose and hemicellulose. Carbohydr. Polym. **82**, 39–45 (2010). 10.1016/j.carbpol.2010.04.018

[46] S. Yu, L. Wang, Q. Li, Y. Zhang, H. Zhou, Sustainable carbon materials from the pyrolysis of lignocellulosic biomass. Mater. Today Sustain. **19**, 100209 (2022). 10.1016/j.mtsust.2022.100209

[47] C. Mukarakate, A. Mittal, P.N. Ciesielski, S. Budhi, L. Thompson et al., Influence of crystal allomorph and crystallinity on the products and behavior of cellulose during fast pyrolysis. ACS Sustain. Chem. Eng. **4**, 4662–4674 (2016). 10.1021/acssuschemeng.6b00812

[48] J. Deng, T. Xiong, H. Wang, A. Zheng, Y. Wang, Effects of cellulose, hemicellulose, and lignin on the structure and morphology of porous carbons. ACS Sustain. Chem. Eng. **4**, 3750–3756 (2016). 10.1021/acssuschemeng.6b00388

[49] W.-J. Liu, H. Jiang, H.-Q. Yu, Development of biochar-based functional materials: toward a sustainable platform carbon material. Chem. Rev. **115**, 12251–12285 (2015). 10.1021/acs.chemrev.5b00195 26495747 10.1021/acs.chemrev.5b00195

[50] B. Zhang, C.M. Ghimbeu, C. Laberty, C. Vix-Guterl, J.-M. Tarascon, Correlation between microstructure and Na storage behavior in hard carbon. Adv. Energy Mater. **6**, 1501588 (2016). 10.1002/aenm.201501588

[51] V. Simone, A. Boulineau, A. de Geyer, D. Rouchon, L. Simonin et al., Hard carbon derived from cellulose as anode for sodium ion batteries: dependence of electrochemical properties on structure. J. Energy Chem. **25**, 761–768 (2016). 10.1016/j.jechem.2016.04.016

[52] Q. Lu, H.-Y. Tian, B. Hu, X.-Y. Jiang, C.-Q. Dong et al., Pyrolysis mechanism of holocellulose-based monosaccharides: the formation of hydroxyacetaldehyde. J. Anal. Appl. Pyrolysis **120**, 15–26 (2016). 10.1016/j.jaap.2016.04.003

[53] X. Zhang, W. Yang, C. Dong, Levoglucosan formation mechanisms during cellulose pyrolysis. J. Anal. Appl. Pyrolysis **104**, 19–27 (2013). 10.1016/j.jaap.2013.09.015

[54] H.B. Mayes, L.J. Broadbelt, Unraveling the reactions that unravel cellulose. J. Phys. Chem. A **116**, 7098–7106 (2012). 10.1021/jp300405x 22686569 10.1021/jp300405x

[55] X. Bai, P. Johnston, S. Sadula, R.C. Brown, Role of levoglucosan physiochemistry in cellulose pyrolysis. J. Anal. Appl. Pyrolysis **99**, 58–65 (2013). 10.1016/j.jaap.2012.10.028

[56] M.J. Antal, Biomass Pyrolysis: A Review of the Literature Part 2—Lignocellulose Pyrolysis, in *Advances in Solar Energy*. (Springer, Boston, 1985), pp.175–255

[57] A.R. Teixeira, K.G. Mooney, J.S. Kruger, C.L. Williams, W.J. Suszynski et al., Aerosol generation by reactive boiling ejection of molten cellulose. Energy Environ. Sci. **4**, 4306 (2011). 10.1039/c1ee01876k

[58] Z. Wang, A.G. McDonald, R.J.M. Westerhof, S.R.A. Kersten, C.M. Cuba-Torres et al., Effect of cellulose crystallinity on the formation of a liquid intermediate and on product distribution during pyrolysis. J. Anal. Appl. Pyrolysis **100**, 56–66 (2013). 10.1016/j.jaap.2012.11.017

[59] D. Liu, Y. Yu, H. Wu, Differences in water-soluble intermediates from slow pyrolysis of amorphous and crystalline cellulose. Energy Fuels **27**, 1371–1380 (2013). 10.1021/ef301823g

[60] Z. Wang, B. Pecha, R.J.M. Westerhof, S.R.A. Kersten, C.-Z. Li et al., Effect of cellulose crystallinity on solid/liquid phase reactions responsible for the formation of carbonaceous residues during pyrolysis. Ind. Eng. Chem. Res. **53**, 2940–2955 (2014). 10.1021/ie4014259

[61] M. Zhang, Z. Geng, Y. Yu, Density functional theory (DFT) study on the dehydration of cellulose. Energy Fuels **25**, 2664–2670 (2011). 10.1021/ef101619e

[62] D. Alvira, D. Antorán, J.J. Manyà, Plant-derived hard carbon as anode for sodium-ion batteries: a comprehensive review to guide interdisciplinary research. Chem. Eng. J. **447**, 137468 (2022). 10.1016/j.cej.2022.137468

[63] Q. Jin, W. Li, K. Wang, P. Feng, H. Li et al., Experimental design and theoretical calculation for sulfur-doped carbon nanofibers as a high performance sodium-ion battery anode. J. Mater. Chem. A **7**, 10239–10245 (2019). 10.1039/c9ta02107h

[64] L. Li, L. Hou, J. Cheng, T. Simmons, F. Zhang et al., A flexible carbon/sulfur-cellulose core-shell structure for advanced lithium–sulfur batteries. Energy Storage Mater. **15**, 388–395 (2018). 10.1016/j.ensm.2018.08.019

[65] W. Lei, D. Jin, H. Liu, Z. Tong, H. Zhang, An overview of bacterial cellulose in flexible electrochemical energy storage. Chemsuschem **13**, 3731–3753 (2020). 10.1002/cssc.202001019 32394542 10.1002/cssc.202001019

[66] D. Xu, C. Chen, J. Xie, B. Zhang, L. Miao et al., A hierarchical N/S-codoped carbon anode fabricated facilely from cellulose/polyaniline microspheres for high-performance sodium-ion batteries. Adv. Energy Mater. **6**, 1501929 (2016). 10.1002/aenm.201501929

[67] H. Jia, N. Sun, M. Dirican, Y. Li, C. Chen et al., Electrospun kraft lignin/cellulose acetate-derived nanocarbon network as an anode for high-performance sodium-ion batteries. ACS Appl. Mater. Interfaces **10**, 44368–44375 (2018). 10.1021/acsami.8b13033 30507154 10.1021/acsami.8b13033

[68] W. Zhang, B. Liu, M. Yang, Y. Liu, H. Li et al., Biowaste derived porous carbon sponge for high performance supercapacitors. J. Mater. Sci. Technol. **95**, 105–113 (2021). 10.1016/j.jmst.2021.03.066

[69] B. Yan, L. Feng, J. Zheng, Q. Zhang, Y. Dong et al., Nitrogen-doped carbon layer on cellulose derived free-standing carbon paper for high-rate supercapacitors. Appl. Surf. Sci. **608**, 155144 (2023). 10.1016/j.apsusc.2022.155144

[70] H. Wang, F. Sun, Z. Qu, K. Wang, L. Wang et al., Oxygen functional group modification of cellulose-derived hard carbon for enhanced sodium ion storage. ACS Sustain. Chem. Eng. **7**, 18554–18565 (2019). 10.1021/acssuschemeng.9b04676

[71] J.J. Manyà, Pyrolysis for biochar purposes: a review to establish current knowledge gaps and research needs. Environ. Sci. Technol. **46**, 7939–7954 (2012). 10.1021/es301029g 22775244 10.1021/es301029g

[72] H. Zhu, F. Shen, W. Luo, S. Zhb, M. Zhao et al., Low temperature carbonization of cellulose nanocrystals for high performance carbon anode of sodium-ion batteries. Nano Energy **33**, 37–44 (2017). 10.1016/j.nanoen.2017.01.021

[73] X. Liu, T. Wang, T. Zhang, Z. Sun, T. Ji et al., Solvated sodium storage via a coadsorptive mechanism in microcrystalline graphite fiber. Adv. Energy Mater. **12**, 2202388 (2022). 10.1002/aenm.202202388

[74] J. Jiang, J. Zhu, W. Ai, Z. Fan, X. Shen et al., Evolution of disposable bamboo chopsticks into uniform carbon fibers: a smart strategy to fabricate sustainable anodes for Li-ion batteries. Energy Environ. Sci. **7**, 2670–2679 (2014). 10.1039/C4EE00602J

[75] T. Zhang, L. Yang, X. Yan, X. Ding, Recent advances of cellulose-based materials and their promising application in sodium-ion batteries and capacitors. Small **14**, e1802444 (2018). 10.1002/smll.201802444 30198091 10.1002/smll.201802444

[76] F. Wang, X. Shi, J. Zhang, T. He, L. Yang et al., Bacterial cellulose-derived micro/mesoporous carbon anode materials controlled by poly(methyl methacrylat**e** for fast sodium ion transport. Nanoscale **14**, 3609–3617 (2022). 10.1039/D1NR07879H 35188164 10.1039/d1nr07879h

[77] V. Agarwal, G.W. Huber, W.C. Conner Jr., S.M. Auerbach, Simulating infrared spectra and hydrogen bonding in cellulose Iβ at elevated temperatures. J. Chem. Phys. **135**, 134506 (2011). 10.1063/1.3646306 21992323 10.1063/1.3646306

[78] L. Xie, G. Sun, F. Su, X. Guo, Q. Kong et al., Hierarchical porous carbon microtubes derived from willow catkins for supercapacitor applications. J. Mater. Chem. A **4**(5), 1637–1646 (2016). 10.1039/c5ta09043a

[79] Z.-E. Yu, Y. Lyu, Y. Wang, S. Xu, H. Cheng et al., Hard carbon micro-nano tubes derived from kapok fiber as anode materials for sodium-ion batteries and the sodium-ion storage mechanism. Chem. Commun. **56**, 778–781 (2020). 10.1039/c9cc08221b 10.1039/c9cc08221b 31845678

[80] C. Yang, J. Xiong, X. Ou, C.-F. Wu, X. Xiong et al., A renewable natural cotton derived and nitrogen/sulfur Co-doped carbon as a high-performance sodium ion battery anode. Mater. Today Energy **8**, 37–44 (2018). 10.1016/j.mtener.2018.02.001

[81] Y. Li, Y.-S. Hu, M.-M. Titirici, L. Chen, X. Huang, Hard carbon microtubes made from renewable cotton as high-performance anode material for sodium-ion batteries. Adv. Energy Mater. **6**, 1600659 (2016). 10.1002/aenm.201600659

[82] F. Wang, T. Zhang, F. Ran, Insights into sodium-ion batteries through plateau and slope regions in cyclic voltammetry by tailoring bacterial cellulose precursors. Electrochim. Acta **441**, 141770 (2023). 10.1016/j.electacta.2022.141770

[83] X. Yu, L. Xin, X. Li, Z. Wu, Y. Liu, Completely crystalline carbon containing graphite-like crystal enables 99.5% initial coulombic efficiency for Na-ion batteries. Mater. Today **59**, 25–35 (2022). 10.1016/j.mattod.2022.07.013

[84] X. Yin, Z. Lu, J. Wang, X. Feng, S. Roy et al., Enabling fast Na^+^ transfer kinetics in the whole-voltage-region of hard-carbon anodes for ultrahigh-rate sodium storage. Adv. Mater. **34**, e2109282 (2022). 10.1002/adma.202109282 35075693 10.1002/adma.202109282

[85] X. Han, S. Zhou, H. Liu, H. Leng, S. Li et al., Noncrystalline carbon anodes for advanced sodium-ion storage. Small Methods **7**, e2201508 (2023). 10.1002/smtd.202201508 36710249 10.1002/smtd.202201508

[86] A.G. Pandolfo, A.F. Hollenkamp, Carbon properties and their role in supercapacitors. J. Power. Sources **157**, 11–27 (2006). 10.1016/j.jpowsour.2006.02.065

[87] L. Yang, M. Hu, Q. Lv, H. Zhang, W. Yang et al., Salt and sugar derived high power carbon microspheres anode with excellent low-potential capacity. Carbon **163**, 288–296 (2020). 10.1016/j.carbon.2020.03.021

[88] W. Li, J. Huang, L. Feng, L. Cao, Y. Ren et al., Controlled synthesis of macroscopic three-dimensional hollow reticulate hard carbon as long-life anode materials for Na-ion batteries. J. Alloys Compd. **716**, 210–219 (2017). 10.1016/j.jallcom.2017.05.062

[89] H. Yang, R. Xu, Y. Yu, A facile strategy toward sodium-ion batteries with ultra-long cycle life and high initial Coulombic Efficiency: free-standing porous carbon nanofiber film derived from bacterial cellulose. Energy Storage Mater. **22**, 105–112 (2019). 10.1016/j.ensm.2019.01.003

[90] Z. Tang, R. Zhang, H. Wang, S. Zhou, Z. Pan et al., Revealing the closed pore formation of waste wood-derived hard carbon for advanced sodium-ion battery. Nat. Commun. **14**, 6024 (2023). 10.1038/s41467-023-39637-5 37758706 10.1038/s41467-023-39637-5 PMC10533848

[91] Y. Zhao, Z. Hu, C. Fan, P. Gao, R. Zhang et al., Novel structural design and adsorption/insertion coordinating quasi-metallic Na storage mechanism toward high-performance hard carbon anode derived from carboxymethyl cellulose. Small **19**, e2303296 (2023). 10.1002/smll.202303296 37294167 10.1002/smll.202303296

[92] Y. He, P. Bai, S. Gao, Y. Xu, Marriage of an ether-based electrolyte with hard carbon anodes creates superior sodium-ion batteries with high mass loading. ACS Appl. Mater. Interfaces **10**, 41380–41388 (2018). 10.1021/acsami.8b15274 30403338 10.1021/acsami.8b15274

[93] R. Tian, S.-H. Park, P.J. King, G. Cunningham, J. Coelho et al., Quantifying the factors limiting rate performance in battery electrodes. Nat. Commun. **10**, 1933 (2019). 10.1038/s41467-019-09792-9 31036866 10.1038/s41467-019-09792-9 PMC6488605

[94] R. Tian, M. Breshears, D.V. Horvath, J.N. Coleman, The rate performance of two-dimensional material-based battery electrodes may not be as good as commonly believed. ACS Nano **14**, 3129–3140 (2020). 10.1021/acsnano.9b08304 32027485 10.1021/acsnano.9b08304

[95] L.L. Wong, H. Chen, S. Adams, Design of fast ion conducting cathode materials for grid-scale sodium-ion batteries. Phys. Chem. Chem. Phys. **19**, 7506–7523 (2017). 10.1039/c7cp00037e 28246664 10.1039/c7cp00037e

[96] C. Heubner, J. Seeba, T. Liebmann, A. Nickol, S. Börner et al., Semi-empirical master curve concept describing the rate capability of lithium insertion electrodes. J. Power. Sources **380**, 83–91 (2018). 10.1016/j.jpowsour.2018.01.077

[97] D.V. Horváth, J. Coelho, R. Tian, V. Nicolosi, J.N. Coleman, Quantifying the dependence of battery rate performance on electrode thickness. ACS Appl. Energy Mater. **3**, 10154–10163 (2020). 10.1021/acsaem.0c01865

[98] S. Alvin, D. Yoon, C. Chandra, R.F. Susanti, W. Chang et al., Extended flat voltage profile of hard carbon synthesized using a two-step carbonization approach as an anode in sodium ion batteries. J. Power. Sources **430**, 157–168 (2019). 10.1016/j.jpowsour.2019.05.013

[99] D.A. Stevens, J.R. Dahn, High capacity anode materials for rechargeable sodium-ion batteries. J. Electrochem. Soc. **147**, 1271 (2000). 10.1149/1.1393348

[100] Y. Cao, L. Xiao, M.L. Sushko, W. Wang, B. Schwenzer et al., Sodium ion insertion in hollow carbon nanowires for battery applications. Nano Lett. **12**, 3783–3787 (2012). 10.1021/nl3016957 22686335 10.1021/nl3016957

[101] C. Bommier, T.W. Surta, M. Dolgos, X. Ji, New mechanistic insights on Na-ion storage in nongraphitizable carbon. Nano Lett. **15**, 5888–5892 (2015). 10.1021/acs.nanolett.5b01969 26241159 10.1021/acs.nanolett.5b01969

[102] S. Alvin, D. Yoon, C. Chandra, H.S. Cahyadi, J.-H. Park et al., Revealing sodium ion storage mechanism in hard carbon. Carbon **145**, 67–81 (2019). 10.1016/j.carbon.2018.12.112

[103] X. Dou, I. Hasa, D. Saurel, C. Vaalma, L. Wu et al., Hard carbons for sodium-ion batteries: structure, analysis, sustainability, and electrochemistry. Mater. Today **23**, 87–104 (2019). 10.1016/j.mattod.2018.12.040

[104] T.-C. Liu, W.G. Pell, B.E. Conway, S.L. Roberson, Behavior of molybdenum nitrides as materials for electrochemical capacitors: comparison with ruthenium oxide. J. Electrochem. Soc. **145**, 1882–1888 (1998). 10.1149/1.1838571

[105] B.E. Conway, W.G. Pell, Double-layer and pseudocapacitance types of electrochemical capacitors and their applications to the development of hybrid devices. J. Solid State Electrochem. **7**, 637–644 (2003). 10.1007/s10008-003-0395-7

[106] J. Wang, J. Polleux, J. Lim, B. Dunn, Pseudocapacitive contributions to electrochemical energy storage in TiO_2_ (anatas**e** nanoparticles. J. Phys. Chem. C **111**, 14925–14931 (2007). 10.1021/jp074464w

[107] H. Lu, F. Ai, Y. Jia, C. Tang, X. Zhang et al., Exploring sodium-ion storage mechanism in hard carbons with different microstructure prepared by ball-milling method. Small **14**, e1802694 (2018). 10.1002/smll.201802694 30175558 10.1002/smll.201802694

[108] Z. Lu, J. Wang, W. Feng, X. Yin, X. Feng et al., Zinc single-atom-regulated hard carbons for high-rate and low-temperature sodium-ion batteries. Adv. Mater. **35**, e2211461 (2023). 10.1002/adma.202211461 36946678 10.1002/adma.202211461

[109] M. Anji Reddy, M. Helen, A. Groß, M. Fichtner, H. Euchner, Insight into sodium insertion and the storage mechanism in hard carbon. ACS Energy Lett. **3**, 2851–2857 (2018). 10.1021/acsenergylett.8b01761

[110] Y. Youn, B. Gao, A. Kamiyama, K. Kubota, S. Komaba et al., Nanometer-size Na cluster formation in micropore of hard carbon as origin of higher-capacity Na-ion battery. NPJ Comput. Mater. **7**, 48 (2021). 10.1038/s41524-021-00515-7

[111] P. Wang, K. Zhu, K. Ye, Z. Gong, R. Liu et al., Three-dimensional biomass derived hard carbon with reconstructed surface as a free-standing anode for sodium-ion batteries. J. Colloid Interface Sci. **561**, 203–210 (2020). 10.1016/j.jcis.2019.11.091 31816465 10.1016/j.jcis.2019.11.091

[112] Y. Chen, F. Li, Z. Guo, Z. Song, Y. Lin et al., Sustainable and scalable fabrication of high-performance hard carbon anode for Na-ion battery. J. Power. Sources **557**, 232534 (2023). 10.1016/j.jpowsour.2022.232534

[113] S. Zhou, Z. Tang, Z. Pan, Y. Huang, L. Zhao et al., Regulating closed pore structure enables significantly improved sodium storage for hard carbon pyrolyzing at relatively low temperature. SusMat **2**, 357–367 (2022). 10.1002/sus2.60

[114] J.-L. Xia, D. Yan, L.-P. Guo, X.-L. Dong, W.-C. Li et al., Hard carbon nanosheets with uniform ultramicropores and accessible functional groups showing high realistic capacity and superior rate performance for sodium-ion storage. Adv. Mater. **32**, e2000447 (2020). 10.1002/adma.202000447 32253798 10.1002/adma.202000447

[115] H. Au, H. Alptekin, A.C.S. Jensen, E. Olsson, C.A. O’Keefe et al., A revised mechanistic model for sodium insertion in hard carbons. Energy Environ. Sci. **13**, 3469–3479 (2020). 10.1039/D0EE01363C

[116] Y. Morikawa, S.-I. Nishimura, R.-I. Hashimoto, M. Ohnuma, A. Yamada, Mechanism of sodium storage in hard carbon: an X-ray scattering analysis. Adv. Energy Mater. **10**, 1903176 (2020). 10.1002/aenm.201903176

[117] V. Surendran, R.K. Hema, M.S.O. Hassan, V. Vijayan, M.M. Shaijumon, Open or closed? Elucidating the correlation between micropore nature and sodium storage mechanisms in hard carbon. Batter. Supercaps **5**, 2200316 (2022). 10.1002/batt.202200316

[118] T.G.T.A. Bandara, J.C. Viera, M. González, The next generation of fast charging methods for Lithium-ion batteries: the natural current-absorption methods. Renew. Sustain. Energy Rev. **162**, 112338 (2022). 10.1016/j.rser.2022.112338

[119] C. Heubner, M. Schneider, A. Michaelis, Investigation of charge transfer kinetics of Li-intercalation in LiFePO_4_. J. Power. Sources **288**, 115–120 (2015). 10.1016/j.jpowsour.2015.04.103

[120] T. Zhang, F. Ran, Design strategies of 3D carbon-based electrodes for charge/ion transport in lithium ion battery and sodium ion battery. Adv. Funct. Mater. **31**, 2010041 (2021). 10.1002/adfm.202010041

[121] D.R. Rolison, J.W. Long, J.C. Lytle, A.E. Fischer, C.P. Rhodes et al., Multifunctional 3D nanoarchitectures for energy storage and conversion. Chem. Soc. Rev. **38**, 226–252 (2009). 10.1039/B801151F 19088976 10.1039/b801151f

[122] K. Yu, X. Wang, H. Yang, Y. Bai, C. Wu, Insight to defects regulation on sugarcane waste-derived hard carbon anode for sodium-ion batteries. J. Energy Chem. **55**, 499–508 (2021). 10.1016/j.jechem.2020.07.025

[123] T. Lyu, X. Lan, L. Liang, X. Lin, C. Hao et al., Natural mushroom spores derived hard carbon plates for robust and low-potential sodium ion storage. Electrochim. Acta **365**, 137356 (2021). 10.1016/j.electacta.2020.137356

[124] B. Yin, S. Liang, D. Yu, B. Cheng, I.L. Egun et al., Increasing accessible subsurface to improving rate capability and cycling stability of sodium-ion batteries. Adv. Mater. **33**, e2100808 (2021). 10.1002/adma.202100808 34337787 10.1002/adma.202100808

[125] X. Yin, Y. Zhao, X. Wang, X. Feng, Z. Lu et al., Modulating the graphitic domains of hard carbons derived from mixed pitch and resin to achieve high rate and stable sodium storage. Small **18**, e2105568 (2022). 10.1002/smll.202105568 34850549 10.1002/smll.202105568

[126] M.E. Lee, H.W. Kwak, H.-J. Jin, Y.S. Yun, Waste beverage coffee-induced hard carbon granules for sodium-ion batteries. ACS Sustain. Chem. Eng. **7**, 12734–12740 (2019). 10.1021/acssuschemeng.9b00971

[127] F. Sun, H. Wang, Z. Qu, K. Wang, L. Wang et al., Carboxyl-dominant oxygen rich carbon for improved sodium ion storage: synergistic enhancement of adsorption and intercalation mechanisms. Adv. Energy Mater. **11**, 2002981 (2021). 10.1002/aenm.202002981

[128] K. Kang, Y.S. Meng, J. Bréger, C.P. Grey, G. Ceder, Electrodes with high power and high capacity for rechargeable lithium batteries. Science **311**, 977–980 (2006). 10.1126/science.1122152 16484487 10.1126/science.1122152

[129] B. Marinho, M. Ghislandi, E. Tkalya, C.E. Koning, G. de With, Electrical conductivity of compacts of graphene, multi-wall carbon nanotubes, carbon black, and graphite powder. Powder Technol. **221**, 351–358 (2012). 10.1016/j.powtec.2012.01.024

[130] S.-M. Zheng, Y.-R. Tian, Y.-X. Liu, S. Wang, C.-Q. Hu et al., Alloy anodes for sodium-ion batteries. Rare Met. **40**, 272–289 (2021). 10.1007/s12598-020-01605-z

[131] K. Cui, C. Wang, Y. Luo, L. Li, J. Gao et al., Enhanced sodium storage kinetics of nitrogen rich cellulose-derived hierarchical porous carbon via subsequent boron doping. Appl. Surf. Sci. **531**, 147302 (2020). 10.1016/j.apsusc.2020.147302

[132] J. Wang, Z. Xu, J.-C. Eloi, M.-M. Titirici, S.J. Eichhorn, Ice-templated, sustainable carbon aerogels with hierarchically tailored channels for sodium- and potassium-ion batteries. Adv. Funct. Mater. **32**, 2110862 (2022). 10.1002/adfm.202110862

[133] T. Zhang, J. Chen, B. Yang, H. Li, S. Lei et al., Enhanced capacities of carbon nanosheets derived from functionalized bacterial cellulose as anodes for sodium ion batteries. RSC Adv. **7**, 50336–50342 (2017). 10.1039/C7RA10118J

[134] F. Xie, Z. Xu, A.C.S. Jensen, H. Au, Y. Lu et al., Hard–soft carbon composite anodes with synergistic sodium storage performance. Adv. Funct. Mater. **29**, 1901072 (2019). 10.1002/adfm.201901072

[135] Q. Shi, D. Liu, Y. Wang, Y. Zhao, X. Yang et al., High-performance sodium-ion battery anode via rapid microwave carbonization of natural cellulose nanofibers with graphene initiator. Small **15**, e1901724 (2019). 10.1002/smll.201901724 31460708 10.1002/smll.201901724

[136] C.-C. Wang, W.-L. Su, Understanding acid pretreatment of lotus leaves to prepare hard carbons as anodes for sodium ion batteries. Surf. Coat. Technol. **415**, 127125 (2021). 10.1016/j.surfcoat.2021.127125

[137] K. Kierzek, J. Machnikowski, Cellulose-derived carbons as a high performance anodic material for Na-ion battery. Ionics **24**, 1313–1320 (2018). 10.1007/s11581-017-2298-0

[138] Z. Xu, Y. Huang, L. Ding, J. Huang, H. Gao et al., Highly stable basswood porous carbon anode activated by phosphoric acid for a sodium ion battery. Energy Fuels **34**, 11565–11573 (2020). 10.1021/acs.energyfuels.0c02286

[139] Y. Li, J. Hu, Z. Wang, K. Yang, W. Huang et al., Low-temperature catalytic graphitization to enhance Na-ion transportation in carbon electrodes. ACS Appl. Mater. Interfaces **11**, 24164–24171 (2019). 10.1021/acsami.9b07206 31250632 10.1021/acsami.9b07206

[140] Z. Li, Z. Jian, X. Wang, I.A. Rodríguez-Pérez, C. Bommier et al., Hard carbon anodes of sodium-ion batteries: undervalued rate capability. Chem. Commun. **53**, 2610–2613 (2017). 10.1039/C7CC00301C 10.1039/c7cc00301c 28195296

[141] J. Borowec, V. Selmert, A. Kretzschmar, K. Fries, R. Schierholz et al., Carbonization-temperature-dependent electrical properties of carbon nanofibers-from nanoscale to macroscale. Adv. Mater. **35**, e2300936 (2023). 10.1002/adma.202300936 37104167 10.1002/adma.202300936

[142] C. Heubner, K. Nikolowski, S. Reuber, M. Schneider, M. Wolter et al., Recent insights into rate performance limitations of Li-ion batteries. Batter. Supercaps **4**(2), 268–285 (2021). 10.1002/batt.202000227

[143] J.-S. Lee, K. Otake, S. Kitagawa, Transport properties in porous coordination polymers. Coord. Chem. Rev. **421**, 213447–213458 (2020). 10.1016/j.ccr.2020.213447

[144] S. Li, K. Wang, G. Zhang, S. Li, Y. Xu et al., Fast charging anode materials for lithium-ion batteries: current status and perspectives. Adv. Funct. Mater. **32**, 2200796 (2022). 10.1002/adfm.202200796

[145] F. Yao, D.T. Pham, Y.H. Lee, Carbon-based materials for lithium-ion batteries, electrochemical capacitors, and their hybrid devices. Chemsuschem **8**, 2284–2311 (2015). 10.1002/cssc.201403490 26140707 10.1002/cssc.201403490

[146] H.-W. Liang, Q.-F. Guan, Z. Zhu, L.-T. Song, H.-B. Yao et al., Highly conductive and stretchable conductors fabricated from bacterial cellulose. NPG Asia Mater. **4**, e19 (2012). 10.1038/am.2012.34

[147] H. Kim, J.-Y. Yi, B.-G. Kim, J.E. Song, H.-J. Jeong et al., Development of cellulose-based conductive fabrics with electrical conductivity and flexibility. PLoS ONE **15**, e0233952 (2020). 10.1371/journal.pone.0233952 32498075 10.1371/journal.pone.0233952 PMC7272206

[148] W. Weppner, R.A. Huggins, Determination of the kinetic parameters of mixed-conducting electrodes and application to the system Li_3_Sb. J. Electrochem. Soc. **124**, 1569–1578 (1977). 10.1149/1.2133112

[149] Y. Chen, Z. Zhang, Y. Lai, X. Shi, J. Li et al., Self-assembly of 3D neat porous carbon aerogels with NaCl as template and flux for sodium-ion batteries. J. Power. Sources **359**, 529–538 (2017). 10.1016/j.jpowsour.2017.05.066

[150] L. Wang, J. Zhao, X. He, J. Gao, J. Li et al., Electrochemical impedance spectroscopy (EIS) study of LiNi_1/3_Co_1/3_Mn_1/3_O_2_ for Li-ion batteries. Int. J. Electrochem. Sci. **7**, 345–353 (2012). 10.1016/s1452-3981(23)13343-8

[151] M. Sotoudeh, S. Baumgart, M. Dillenz, J. Döhn, K. Forster-Tonigold et al., Ion mobility in crystalline battery materials. Adv. Energy Mater. (2023). 10.1002/aenm.202302550

[152] R. Jain, A.S. Lakhnot, K. Bhimani, S. Sharma, V. Mahajani et al., Nanostructuring versus microstructuring in battery electrodes. Nat. Rev. Mater. **7**, 736–746 (2022). 10.1038/s41578-022-00454-9

[153] W. Shao, Q. Cao, S. Liu, T. Zhang, Z. Song et al., Replacing “Alkyl” with “Aryl” for inducing accessible channels to closed pores as plateau-dominated sodium-ion battery anode. SusMat **2**, 319–334 (2022). 10.1002/sus2.68

[154] S.N. Lauro, J.N. Burrow, C.B. Mullins, Restructuring the lithium-ion battery: a perspective on electrode architectures. Science **3**, 100152 (2023). 10.1016/j.esci.2023.100152

[155] H. Sun, J. Zhu, D. Baumann, L. Peng, Y. Xu et al., Hierarchical 3D electrodes for electrochemical energy storage. Nat. Rev. Mater. **4**, 45–60 (2019). 10.1038/s41578-018-0069-9

[156] X. Ding, Y. Xin, Y. Wang, M. Wang, T. Song et al., Artificial solid electrolyte interphase engineering toward dendrite-free lithium anodes. ACS Sustain. Chem. Eng. **11**, 6879–6889 (2023). 10.1021/acssuschemeng.2c06146

[157] M. Yang, L. Chen, H. Li, F. Wu, Air/water stability problems and solutions for lithium batteries. Energy Mater. Adv. **2022**, 9842651 (2022). 10.34133/2022/9842651

[158] C. Bommier, X. Ji, Electrolytes, SEI formation, and binders: a review of nonelectrode factors for sodium-ion battery anodes. Small **14**, e1703576 (2018). 10.1002/smll.201703576 29356418 10.1002/smll.201703576

[159] J. Tan, J. Matz, P. Dong, J. Shen, M. Ye, A growing appreciation for the role of LiF in the solid electrolyte interphase. Adv. Energy Mater. **11**(16), 2100046 (2021). 10.1002/aenm.202100046

[160] Y. Li, F. Wu, Y. Li, M. Liu, X. Feng et al., Ether-based electrolytes for sodium ion batteries. Chem. Soc. Rev. **51**, 4484–4536 (2022). 10.1039/d1cs00948f 35543354 10.1039/d1cs00948f

[161] Q. Wang, J. Li, H. Jin, S. Xin, H. Gao, Prussian-blue materials: revealing new opportunities for rechargeable batteries. InfoMat **4**, e12311 (2022). 10.1002/inf2.12311

[162] J.B. Goodenough, H. Gao, A perspective on the Li-ion battery. Sci. China Chem. **62**, 1555–1556 (2019). 10.1007/s11426-019-9610-3

[163] Q. Wang, X. Ding, J. Li, H. Jin, H. Gao, Minimizing the interfacial resistance for a solid-state lithium battery running at room temperature. Chem. Eng. J. **448**, 137740 (2022). 10.1016/j.cej.2022.137740

[164] J. Zhang, D.-W. Wang, W. Lv, S. Zhang, Q. Liang et al., Achieving superb sodium storage performance on carbon anodes through an ether-derived solid electrolyte interphase. Energy Environ. Sci. **10**, 370–376 (2017). 10.1039/c6ee03367a

[165] L. Zhao, F. Ran, Electrolyte-philicity of electrode materials. Chem. Commun. **59**, 6969–6986 (2023). 10.1039/d3cc00412k 10.1039/d3cc00412k 37165689

[166] A. Karatrantos, Q. Cai, Effects of pore size and surface charge on Na ion storage in carbon nanopores. Phys. Chem. Chem. Phys. **18**, 30761–30769 (2016). 10.1039/c6cp04611h 27796383 10.1039/c6cp04611h

[167] N. Ortiz Vitoriano, I. Ruiz de Larramendi, R.L. Sacci, I. Lozano, C.A. Bridges et al., Goldilocks and the three glymes: how Na^+^ solvation controls Na–O_2_ battery cycling. Energy Storage Mater. **29**, 235–245 (2020). 10.1016/j.ensm.2020.04.034

[168] E. Wang, Y. Niu, Y.-X. Yin, Y.-G. Guo, Manipulating electrode/electrolyte interphases of sodium-ion batteries: strategies and perspectives. ACS Mater. Lett. **3**, 18–41 (2021). 10.1021/acsmaterialslett.0c00356

[169] L. Zhao, Y. Peng, F. Ran, Constructing mutual-philic electrode/non-liquid electrolyte interfaces in electrochemical energy storage systems: reasons, progress, and perspectives. Energy Storage Mater. **58**, 48–73 (2023). 10.1016/j.ensm.2023.03.009

[170] L. Zhao, Y. Li, M. Yu, Y. Peng, F. Ran, Electrolyte-wettability issues and challenges of electrode materials in electrochemical energy storage, energy conversion, and beyond. Adv. Sci. **10**, e2300283 (2023). 10.1002/advs.202300283 10.1002/advs.202300283 PMC10265108 37085907

[171] M. Liu, F. Wu, Y. Gong, Y. Li, Y. Li et al., Interfacial-catalysis-enabled layered and inorganic-rich SEI on hard carbon anodes in ester electrolytes for sodium-ion batteries. Adv. Mater. **35**, e2300002 (2023). 10.1002/adma.202300002 37018163 10.1002/adma.202300002

[172] Z. Wang, H. Yang, Y. Liu, Y. Bai, G. Chen et al., Analysis of the stable interphase responsible for the excellent electrochemical performance of graphite electrodes in sodium-ion batteries. Small **16**, e2003268 (2020). 10.1002/smll.202003268 33244854 10.1002/smll.202003268

[173] M.E. Lee, S.M. Lee, J. Choi, D. Jang, S. Lee et al., Electrolyte-dependent sodium ion transport behaviors in hard carbon anode. Small **16**, e2001053 (2020). 10.1002/smll.202001053 32761802 10.1002/smll.202001053

[174] Y. Meng, C.I. Contescu, P. Liu, S. Wang, S.-H. Lee et al., Understanding the local structure of disordered carbons from cellulose and lignin. Wood Sci. Technol. **55**, 587–606 (2021). 10.1007/s00226-021-01286-6

[175] L. Fan, X. Li, Recent advances in effective protection of sodium metal anode. Nano Energy **53**, 630–642 (2018). 10.1016/j.nanoen.2018.09.017

[176] Q. Li, J. Zhang, L. Zhong, F. Geng, Y. Tao et al., Unraveling the key atomic interactions in determining the varying Li/Na/K storage mechanism of hard carbon anodes. Adv. Energy Mater. **12**, 2201734 (2022). 10.1002/aenm.202201734

[177] T. Zhang, T. Zhang, F. Wang, F. Ran, Pretreatment process before heat pyrolysis of plant-based precursors paving way for fabricating high-performance hard carbon for sodium-ion batteries. ChemElectroChem **10**, 2300442 (2023). 10.1002/celc.202300442

[178] A. Gopalakrishnan, S. Badhulika, Effect of self-doped heteroatoms on the performance of biomass-derived carbon for supercapacitor applications. J. Power. Sources **480**, 228830 (2020). 10.1016/j.jpowsour.2020.228830

[179] X. Feng, Y. Bai, M. Liu, Y. Li, H. Yang et al., Untangling the respective effects of heteroatom-doped carbon materials in batteries, supercapacitors and the ORR to design high performance materials. Energy Environ. Sci. **14**, 2036–2089 (2021). 10.1039/d1ee00166c

[180] H. Wang, E. Zhu, J. Yang, P. Zhou, D. Sun et al., Bacterial cellulose nanofiber-supported polyaniline nanocomposites with flake-shaped morphology as supercapacitor electrodes. J. Phys. Chem. C **116**, 13013–13019 (2012). 10.1021/jp301099r

[181] Y. Zhang, L. Tao, C. Xie, D. Wang, Y. Zou et al., Defect engineering on electrode materials for rechargeable batteries. Adv. Mater. **32**, e1905923 (2020). 10.1002/adma.201905923 31930593 10.1002/adma.201905923

[182] M. Wang, H. Wu, S. Xu, P. Dong, A. Long et al., Cellulose nanocrystal regulated ultra-loose, lightweight, and hierarchical porous reduced graphene oxide hybrid aerogel for capturing and determining organic pollutants from water. Carbon **204**, 94–101 (2023). 10.1016/j.carbon.2022.12.058

[183] Z. Li, C. Bommier, Z.S. Chong, Z. Jian, T.W. Surta et al., Mechanism of Na-ion storage in hard carbon anodes revealed by heteroatom doping. Adv. Energy Mater. **7**, 1602894 (2017). 10.1002/aenm.201602894

[184] L. Li, Q. Wang, X. Zhang, L. Fang, X. Li et al., Unique three-dimensional Co_3_O_4_@N-CNFs derived from ZIFs and bacterial cellulose as advanced anode for sodium-ion batteries. Appl. Surf. Sci. **508**, 145295 (2020). 10.1016/j.apsusc.2020.145295

[185] L. Shi, Y. Li, F. Zeng, S. Ran, C. Dong et al., *In situ* growth of amorphous Fe_2_O_3_ on 3D interconnected nitrogen-doped carbon nanofibers as high-performance anode materials for sodium-ion batteries. Chem. Eng. J. **356**, 107–116 (2019). 10.1016/j.cej.2018.09.018

[186] H. Tao, L. Xiong, S. Du, Y. Zhang, X. Yang et al., Interwoven N and P dual-doped hollow carbon fibers/graphitic carbon nitride: an ultrahigh capacity and rate anode for Li and Na ion batteries. Carbon **122**, 54–63 (2017). 10.1016/j.carbon.2017.06.040

[187] H.-M. Wang, H.-X. Wang, Y. Chen, Y.-J. Liu, J.-X. Zhao et al., Phosphorus-doped graphene and (8, 0) carbon nanotube: structural, electronic, magnetic properties, and chemical reactivity. Appl. Surf. Sci. **273**, 302–309 (2013). 10.1016/j.apsusc.2013.02.035

[188] K.C. Wasalathilake, G.A. Ayoko, C. Yan, Effects of heteroatom doping on the performance of graphene in sodium-ion batteries: a density functional theory investigation. Carbon **140**, 276–285 (2018). 10.1016/j.carbon.2018.08.071

[189] P.A. Denis, Band gap opening of monolayer and bilayer graphene doped with aluminium, silicon, phosphorus, and sulfur. Chem. Phys. Lett. **492**, 251–257 (2010). 10.1016/j.cplett.2010.04.038

[190] A.K. Thakur, K. Kurtyka, M. Majumder, X. Yang, H.Q. Ta et al., Recent advances in boron- and nitrogen-doped carbon-based materials and their various applications. Adv. Mater. Interfaces **9**, 2101964 (2022). 10.1002/admi.202101964

[191] Y. Li, M. Chen, B. Liu, Y. Zhang, X. Liang et al., Heteroatom doping: an effective way to boost sodium ion storage. Adv. Energy Mater. **10**, 2000927 (2020). 10.1002/aenm.202000927

[192] D. Wu, F. Sun, Z. Qu, H. Wang, Z. Lou et al., Multi-scale structure optimization of boron-doped hard carbon nanospheres boosting the plateau capacity for high performance sodium ion batteries. J. Mater. Chem. A **10**, 17225–17236 (2022). 10.1039/D2TA04194D

[193] P. Wang, B. Qiao, Y. Du, Y. Li, X. Zhou et al., Fluorine-doped carbon particles derived from *Lotus* petioles as high-performance anode materials for sodium-ion batteries. J. Phys. Chem. C **119**, 21336–21344 (2015). 10.1021/acs.jpcc.5b05443

[194] Z. Liu, L. Yue, C. Wang, D. Li, L. Tang et al., Free-standing carbon nanofiber composite networks derived from bacterial cellulose and polypyrrole for ultrastable potassium-ion batteries. ACS Appl. Mater. Interfaces **15**(11), 14865–14873 (2023). 10.1021/acsami.3c01401 10.1021/acsami.3c01401 36913555

[195] X.-X. He, J.-H. Zhao, W.-H. Lai, R. Li, Z. Yang et al., Soft-carbon-coated, free-standing, low-defect, hard-carbon anode to achieve a 94% initial coulombic efficiency for sodium-ion batteries. ACS Appl. Mater. Interfaces **13**, 44358–44368 (2021). 10.1021/acsami.1c12171 34506123 10.1021/acsami.1c12171

[196] C. Yang, Q. Wu, W. Xie, X. Zhang, A. Brozena et al., Copper-coordinated cellulose ion conductors for solid-state batteries. Nature **598**, 590–596 (2021). 10.1038/s41586-021-03885-6 34671167 10.1038/s41586-021-03885-6

[197] A. Farooq, M.K. Patoary, M. Zhang, H. Mussana, M. Li et al., Cellulose from sources to nanocellulose and an overview of synthesis and properties of nanocellulose/zinc oxide nanocomposite materials. Int. J. Biol. Macromol. **154**, 1050–1073 (2020). 10.1016/j.ijbiomac.2020.03.163 32201207 10.1016/j.ijbiomac.2020.03.163

[198] Z. Lu, B. Wang, Y. Hu, W. Liu, Y. Zhao et al., An isolated zinc–cobalt atomic pair for highly active and durable oxygen reduction. Angew. Chem. Int. Ed. **58**, 2622–2626 (2019). 10.1002/anie.201810175 10.1002/anie.201810175 30600864

[199] X. Yao, Y. Ke, W. Ren, X. Wang, F. Xiong et al., Defect-rich soft carbon porous nanosheets for fast and high-capacity sodium-ion storage. Adv. Energy Mater. **9**(6), 1803260 (2018). 10.1002/aenm.201803260

[200] G. Qiu, M. Ning, M. Zhang, J. Hu, Z. Duan et al., Flexible hard–soft carbon heterostructure based on mesopore confined carbonization for ultrafast and highly durable sodium storage. Carbon **205**, 310–320 (2023). 10.1016/j.carbon.2023.01.018

[201] D.-C. Wang, H.-Y. Yu, D. Qi, Y. Wu, L. Chen et al., Confined chemical transitions for direct extraction of conductive cellulose nanofibers with graphitized carbon shell at low temperature and pressure. J. Am. Chem. Soc. **143**, 11620–11630 (2021). 10.1021/jacs.1c04710 34286968 10.1021/jacs.1c04710

[202] H. Wang, Y. Shao, S. Mei, Y. Lu, M. Zhang et al., Polymer-derived heteroatom-doped porous carbon materials. Chem. Rev. **120**, 9363–9419 (2020). 10.1021/acs.chemrev.0c00080 32786418 10.1021/acs.chemrev.0c00080

[203] A. Dobashi, J. Maruyama, Y. Shen, M. Nandi, H. Uyama, Activated carbon monoliths derived from bacterial cellulose/polyacrylonitrile composite as new generation electrode materials in EDLC. Carbohydr. Polym. **200**, 381–390 (2018). 10.1016/j.carbpol.2018.08.016 30177178 10.1016/j.carbpol.2018.08.016

[204] Q. Bai, Q. Xiong, C. Li, Y. Shen, H. Uyama, Hierarchical porous carbons from poly(methyl methacrylat**e**/bacterial cellulose composite monolith for high-performance supercapacitor electrodes. ACS Sustain. Chem. Eng. **5**, 9390–9401 (2017). 10.1021/acssuschemeng.7b02488

[205] F. Shen, W. Luo, J. Dai, Y. Yao, M. Zhu et al., Ultra-thick, low-tortuosity, and mesoporous wood carbon anode for high-performance sodium-ion batteries. Adv. Energy Mater. **6**, 1600377 (2016). 10.1002/aenm.201600377

[206] H. Zhao, J. Ye, W. Song, D. Zhao, M. Kang et al., Insights into the surface oxygen functional group-driven fast and stable sodium adsorption on carbon. ACS Appl. Mater. Interfaces **12**, 6991–7000 (2020). 10.1021/acsami.9b11627 31957428 10.1021/acsami.9b11627

[207] R. Guo, C. Lv, W. Xu, J. Sun, Y. Zhu et al., Effect of intrinsic defects of carbon materials on the sodium storage performance. Adv. Energy Mater. **10**, 1903652 (2020). 10.1002/aenm.201903652

[208] L. Xiao, H. Lu, Y. Fang, M.L. Sushko, Y. Cao et al., Low-defect and low-porosity hard carbon with high coulombic efficiency and high capacity for practical sodium ion battery anode. Adv. Energy Mater. **8**, 1703238 (2018). 10.1002/aenm.201703238

[209] Y. Chen, B. Xi, M. Huang, L. Shi, S. Huang et al., Defect-selectivity and “order-in-disorder” engineering in carbon for durable and fast potassium storage. Adv. Mater. **34**, e2108621 (2022). 10.1002/adma.202108621 34850465 10.1002/adma.202108621

[210] M. Wang, Z. Yang, W. Li, L. Gu, Y. Yu, Superior sodium storage in 3D interconnected nitrogen and oxygen dual-doped carbon network. Small **12**, 2559–2566 (2016). 10.1002/smll.201600101 27028729 10.1002/smll.201600101

[211] D. Sun, B. Luo, H. Wang, Y. Tang, X. Ji et al., Engineering the trap effect of residual oxygen atoms and defects in hard carbon anode towards high initial Coulombic efficiency. Nano Energy **64**, 103937 (2019). 10.1016/j.nanoen.2019.103937

[212] C. Matei Ghimbeu, J. Górka, V. Simone, L. Simonin, S. Martinet et al., Insights on the Na^+^ ion storage mechanism in hard carbon: discrimination between the porosity, surface functional groups and defects. Nano Energy **44**, 327–335 (2018). 10.1016/j.nanoen.2017.12.013

[213] X. Tang, F. Xie, Y. Lu, Z. Chen, X. Li et al., Intrinsic effects of precursor functional groups on the Na storage performance in carbon anodes. Nano Res. **16**, 12579–12586 (2023). 10.1007/s12274-023-5643-9

[214] K.-Y. Lee, H. Qian, F.H. Tay, J.J. Blaker, S.G. Kazarian et al., Bacterial cellulose as source for activated nanosized carbon for electric double layer capacitors. J. Mater. Sci. **48**, 367–376 (2013). 10.1007/s10853-012-6754-y

[215] T. Zhang, J. Lang, L. Liu, L. Liu, H. Li et al., Effect of carboxylic acid groups on the supercapacitive performance of functional carbon frameworks derived from bacterial cellulose. Chin. Chem. Lett. **28**, 2212–2218 (2017). 10.1016/j.cclet.2017.08.013

[216] S. Alvin, C. Chandra, J. Kim, Controlling intercalation sites of hard carbon for enhancing Na and K storage performance. Chem. Eng. J. **411**, 128490 (2021). 10.1016/j.cej.2021.128490

[217] Q. Wang, Z. Chen, Q. Luo, H. Li, J. Li et al., Capillary evaporation on high-dense conductive ramie carbon for assisting highly volumetric-performance supercapacitors. Small **19**, e2303349 (2023). 10.1002/smll.202303349 37312646 10.1002/smll.202303349

[218] D.-S. Bin, Y. Li, Y.-G. Sun, S.-Y. Duan, Y. Lu et al., Structural engineering of multishelled hollow carbon nanostructures for high-performance Na-ion battery anode. Adv. Energy Mater. **8**, 1800855 (2018). 10.1002/aenm.201800855

[219] Y. Zhang, Y. Zhu, J. Zhang, S. Sun, C. Wang et al., Optimizing the crystallite structure of lignin-based nanospheres by resinification for high-performance sodium-ion battery anodes. Energy Technol. **8**, 1900694 (2020). 10.1002/ente.201900694

[220] M. Yuan, H. Liu, F. Ran, Fast-charging cathode materials for lithium & sodium ion batteries. Mater. Today **63**, 360–379 (2023). 10.1016/j.mattod.2023.02.007

[221] H. Li, C. Qi, Y. Tao, H. Liu, D.-W. Wang et al., Quantifying the volumetric performance metrics of supercapacitors. Adv. Energy Mater. **9**, 1900079 (2019). 10.1002/aenm.201900079

[222] Q. Li, Y.-N. Zhang, S. Feng, D. Liu, G. Wang et al., N, S self-doped porous carbon with enlarged interlayer distance as anode for high performance sodium ion batteries. Int. J. Energy Res. **45**, 7082–7092 (2021). 10.1002/er.6294

[223] Q. Meng, Y. Lu, F. Ding, Q. Zhang, L. Chen et al., Tuning the closed pore structure of hard carbons with the highest Na storage capacity. ACS Energy Lett. **4**, 2608–2612 (2019). 10.1021/acsenergylett.9b01900

[224] M. Thommes, K. Kaneko, A.V. Neimark, J.P. Olivier, F. Rodriguez-Reinoso et al., Physisorption of gases, with special reference to the evaluation of surface area and pore size distribution (IUPAC Technical Report). Pure Appl. Chem. **87**, 1051–1069 (2015). 10.1515/pac-2014-1117

[225] X.-K. Wang, J. Shi, L.-W. Mi, Y.-P. Zhai, J.-Y. Zhang et al., Hierarchical porous hard carbon enables integral solid electrolyte interphase as robust anode for sodium-ion batteries. Rare Met. **39**, 1053–1062 (2020). 10.1007/s12598-020-01469-3

[226] K. Wang, F. Sun, H. Wang, D. Wu, Y. Chao et al., Altering thermal transformation pathway to create closed pores in coal-derived hard carbon and boosting of Na^+^ plateau storage for high-performance sodium-ion battery and sodium-ion capacitor. Adv. Funct. Mater. **32**, 2203725 (2022). 10.1002/adfm.202203725

[227] J. Yang, X. Wang, W. Dai, X. Lian, X. Cui et al., From micropores to ultra-micropores inside hard carbon: toward enhanced capacity in room-/low-temperature sodium-ion storage. Nano Micro Lett. **13**, 98 (2021). 10.1007/s40820-020-00587-y 10.1007/s40820-020-00587-y PMC8010088 34138264

[228] D. Guo, J. Qin, Z. Yin, J. Bai, Y.-K. Sun et al., Achieving high mass loading of Na_3_V2(PO4)_3_@carbon on carbon cloth by constructing three-dimensional network between carbon fibers for ultralong cycle-life and ultrahigh rate sodium-ion batteries. Nano Energy **45**, 136–147 (2018). 10.1016/j.nanoen.2017.12.038

[229] Y. Li, Y. Lu, Q. Meng, A.C.S. Jensen, Q. Zhang et al., Regulating pore structure of hierarchical porous waste cork-derived hard carbon anode for enhanced Na storage performance. Adv. Energy Mater. **9**, 1902852 (2019). 10.1002/aenm.201902852

